# Supplementary material for: Simple phosphinate ligands access zinc clusters identified in the synthesis of zinc oxide nanoparticles
Source: Nat Commun. 2016 Oct 13;7:13008. doi: 10.1038/ncomms13008 (PMC5065624; doi:10.1038/ncomms13008)
Supplement: Supplementary Information — Supplementary Figures 1-73, Supplementary Tables 1-3, Supplementary Note 1, Supplementary Methods and Supplementary References. [file ncomms13008-s1.pdf]

Spectra and crystal structures of compounds 1A, 2A/B, 3A/B

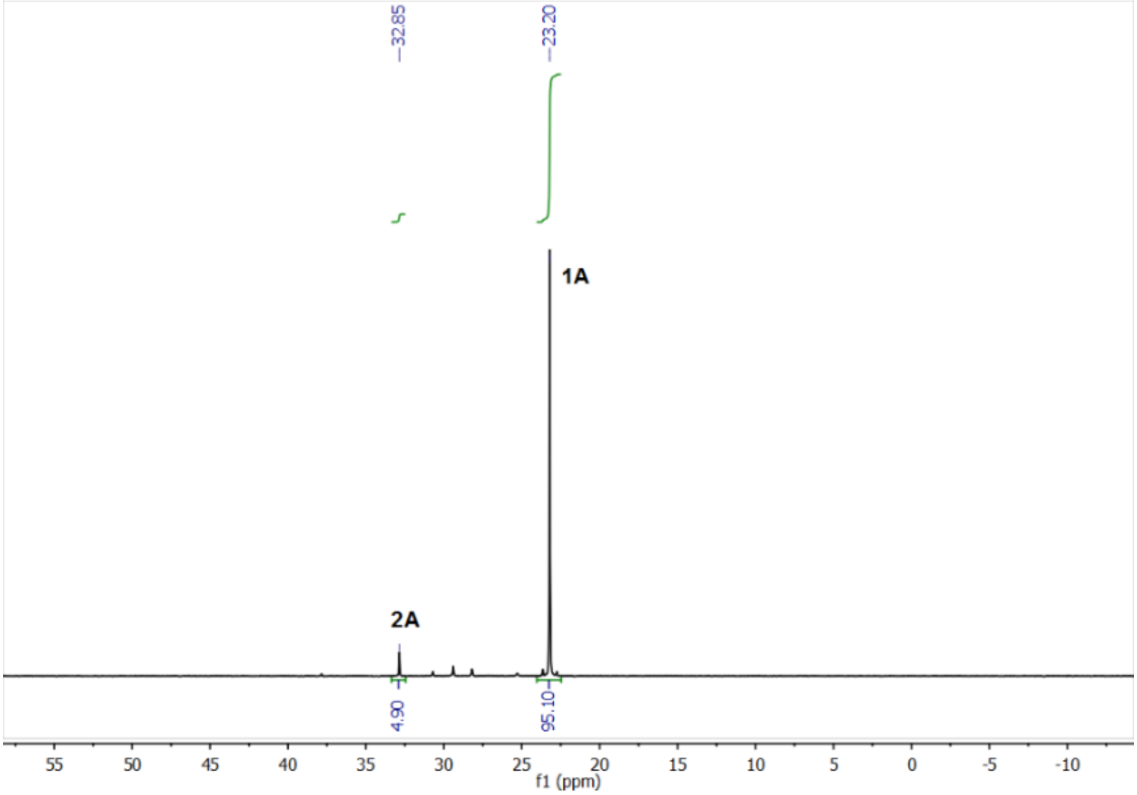

Supplementary Figure 1: $^{31}P\{^{1}H\}$ NMR spectrum of **1A**

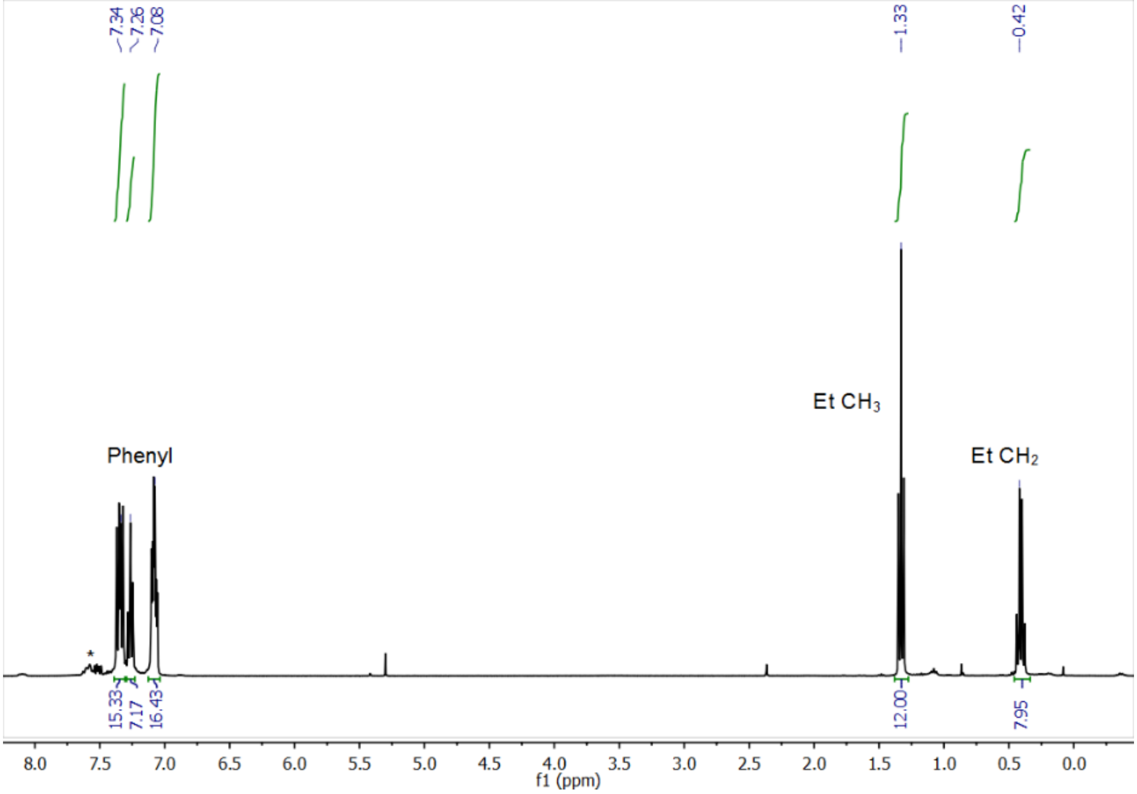

Supplementary Figure 2: ^{1}H NMR spectrum of **1A**, (* refers to a trace of **2A**).

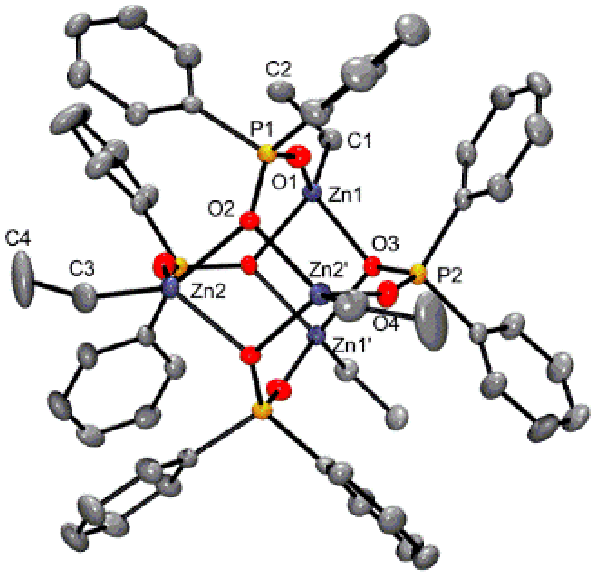

Supplementary Figure 3: Solid state structure of **1A**: Displacement ellipsoids at the 50% probability level, hydrogen atoms omitted for clarity. Selected bond lengths (Å) and angles (°): Zn1-C1, 1.973(2); Zn2-C3, 1.968(3); Zn1-O1, 1.970(2); Zn2’-O4, 1.870(2); Zn1-O3, 2.094(2); Zn2-O2, 2.087(2); P1-O1, 1.492(2); P2-O4, 1.497(2); P1-O2, 1.531(2); P2-O3, 1.534(2); O3-Zn1-O3; 82.78(7); O2-Zn2-O2, 79.85(6); O1-Zn1-C1, 121.46(9); O1-P1-O2, 118.11(9); O3-P2-O4, 116.16(10).

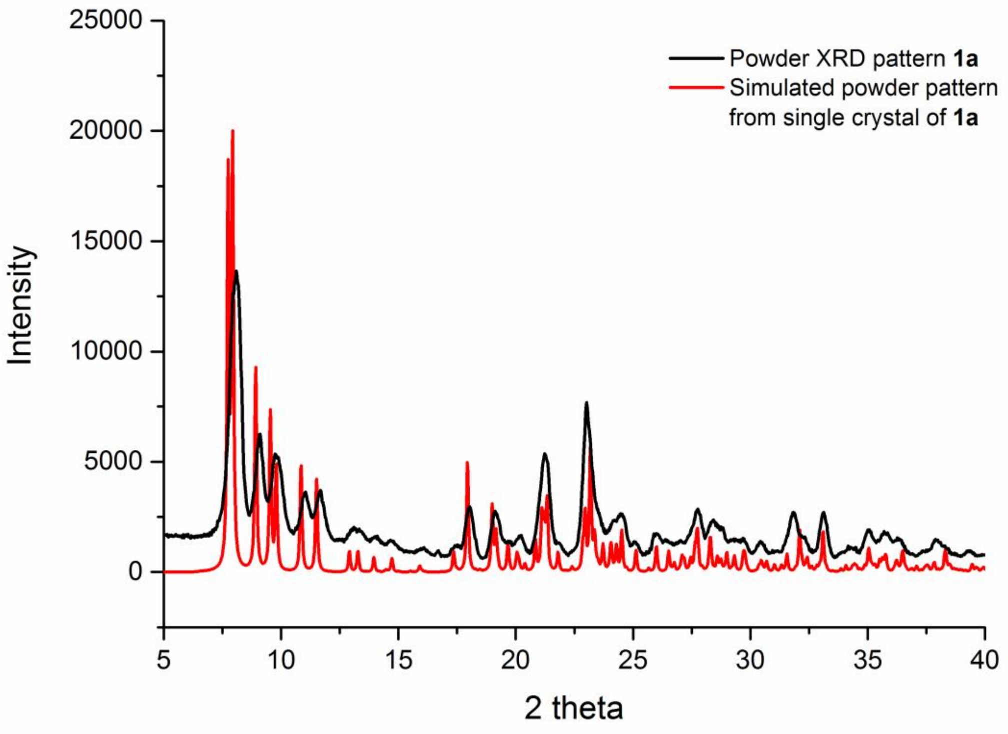

Supplementary Figure 4: Powder X-ray diffraction pattern of a bulk sample of **1A** compared to the simulated powder pattern extracted from single crystal diffraction data.

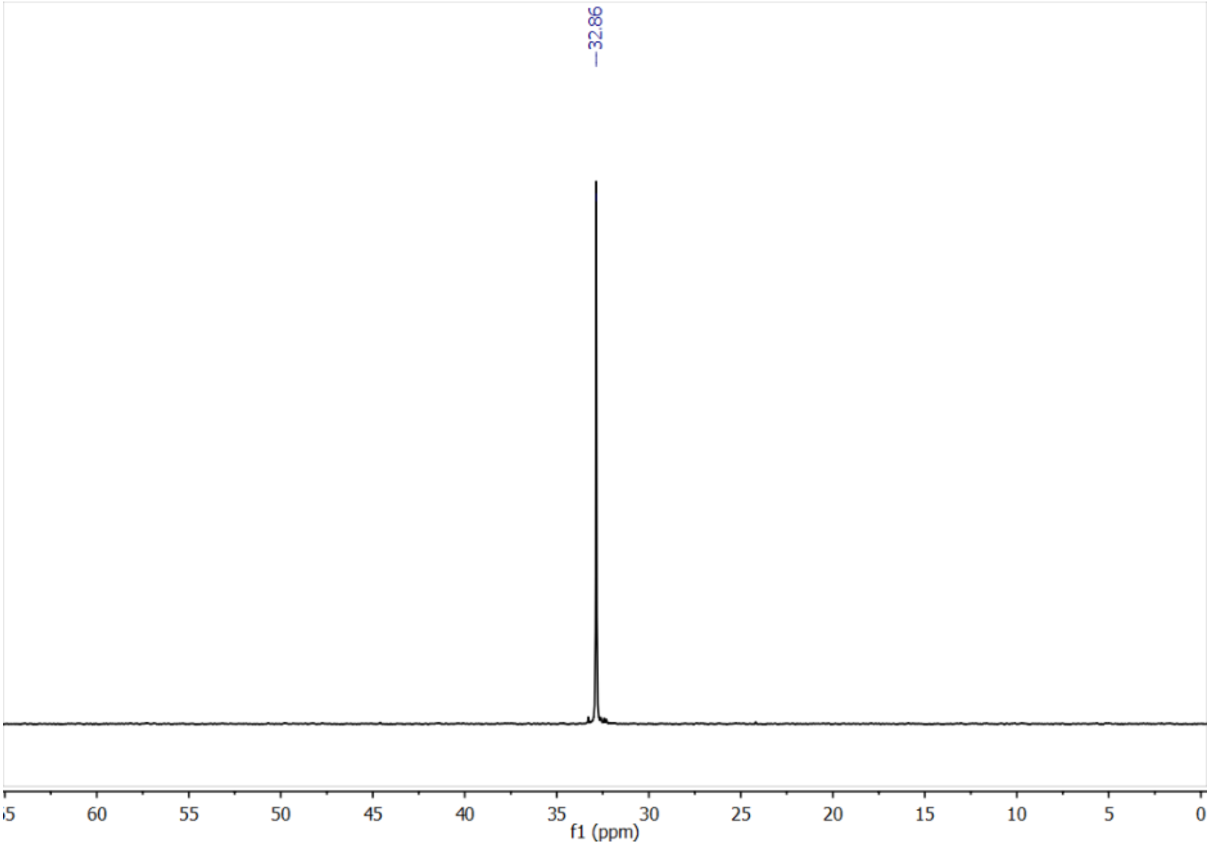

Supplementary Figure 5: $^{31}P\{^{1}H\}$ NMR spectrum of **2A**

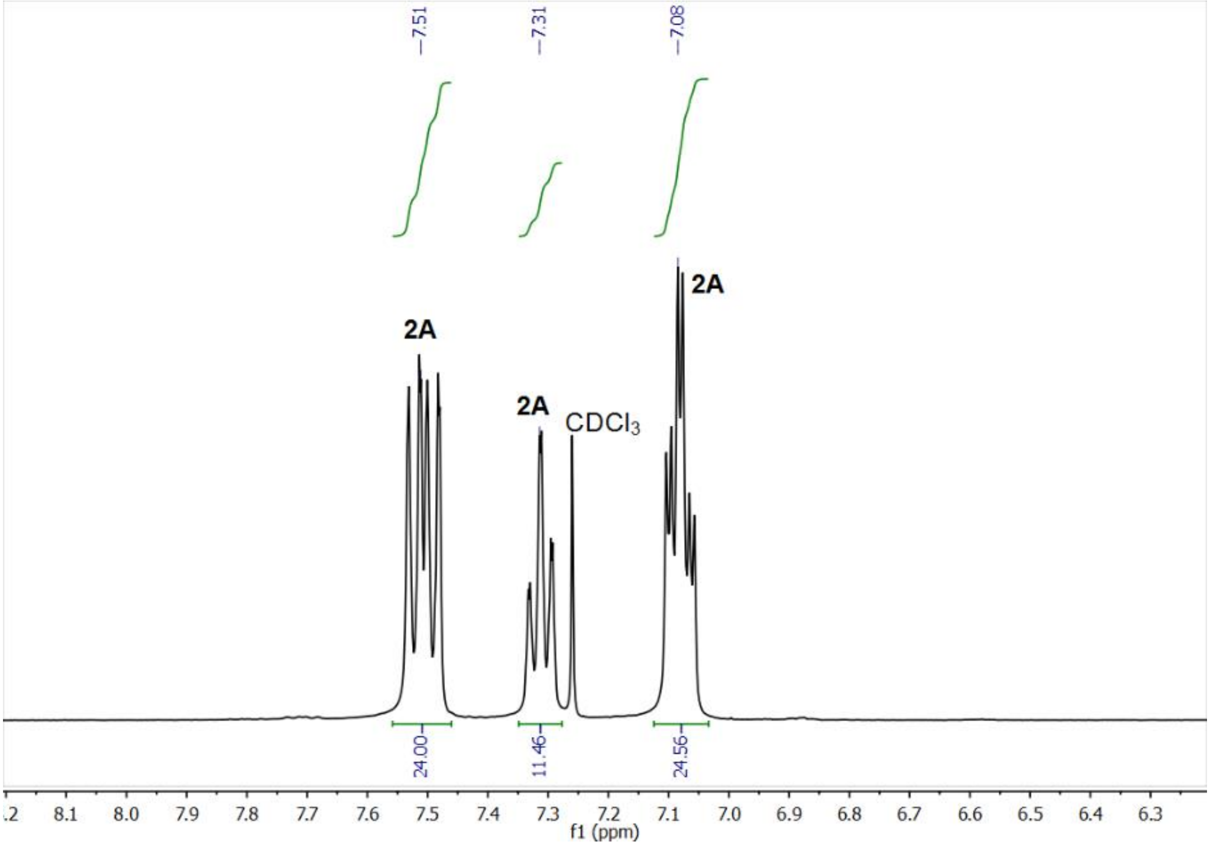

Supplementary Figure 6: ^{1}H NMR spectrum of **2A**

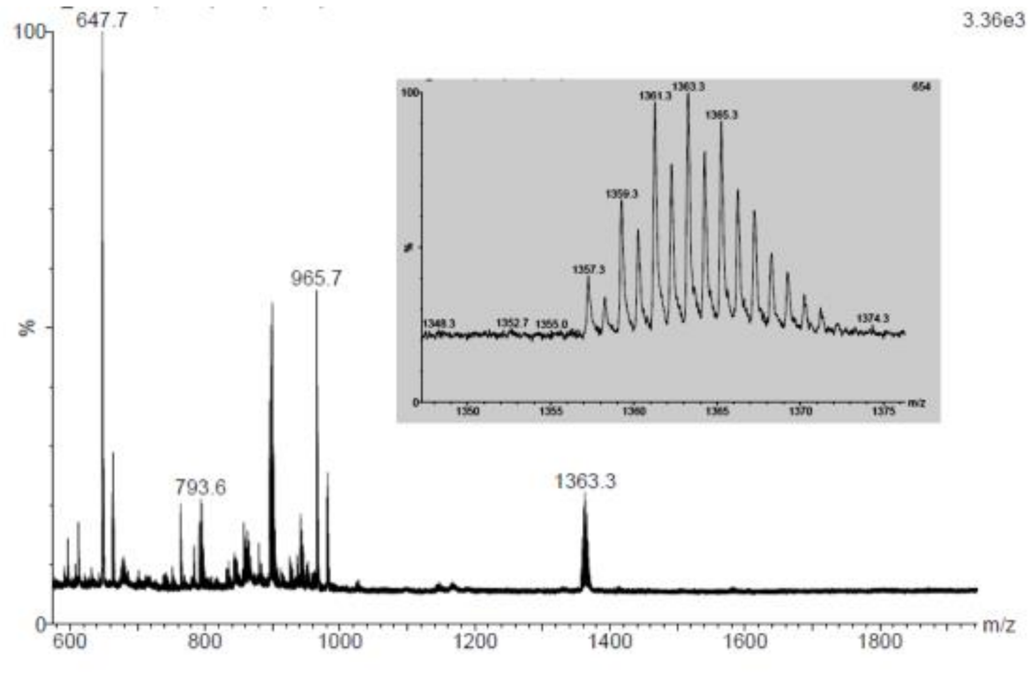

Supplementary Figure 7: MALDI-mass spectrum of **2A**; $[Zn_4O(DPPA)_5]^+$ signal inset.

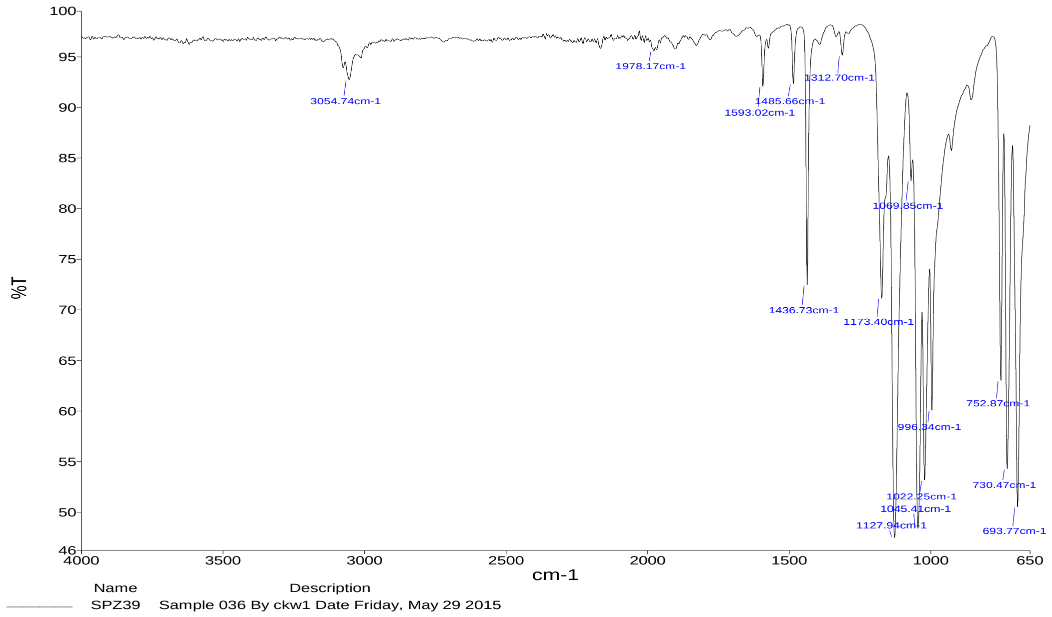

Supplementary Figure 8: I.R. spectrum of **2A**, selected stretches, ν: 3054.7 (w, C-H stretch), 1127.9 (s, PO_2^- stretch), 1045.4 (s, PO_2^- stretch).

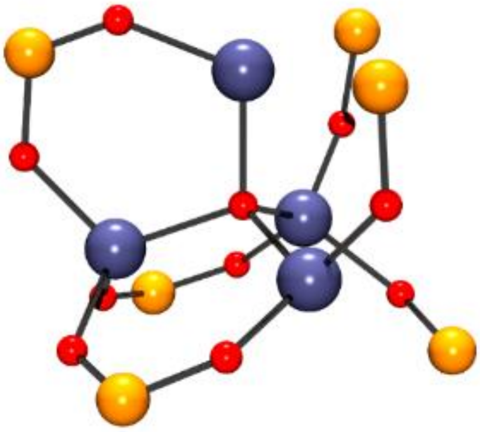

Supplementary Figure 9: Ball and stick representation of one of the four independent molecules of **2A** in its solid state structure. Disordered phenyl groups not shown, some oxygen atoms missing. Crystals of **2A** were grown from toluene or from CH_2Cl_2/Hexane solutions. X-ray diffraction revealed the same unit cell in each case. a, 30.0822(5) Å; b, 30.1296(5) Å; c, 30.2013(4); α, 90; β, 90; γ, 90; V, 27373.4(4) $Å^3$, orthorhombic (P 22_12). This solved to show four independent molecules, however significant disorder of the phosphinate units did not allow for an accurate solution. In each case a Zn_4O unit surrounded by PO_2 fragments was observed in keeping with the predicted structure and the structure of 2**C**. The structural data is not accurate enough for bond length analysis and only shows the connectivity of the core heavy atoms (Zn, P, O).

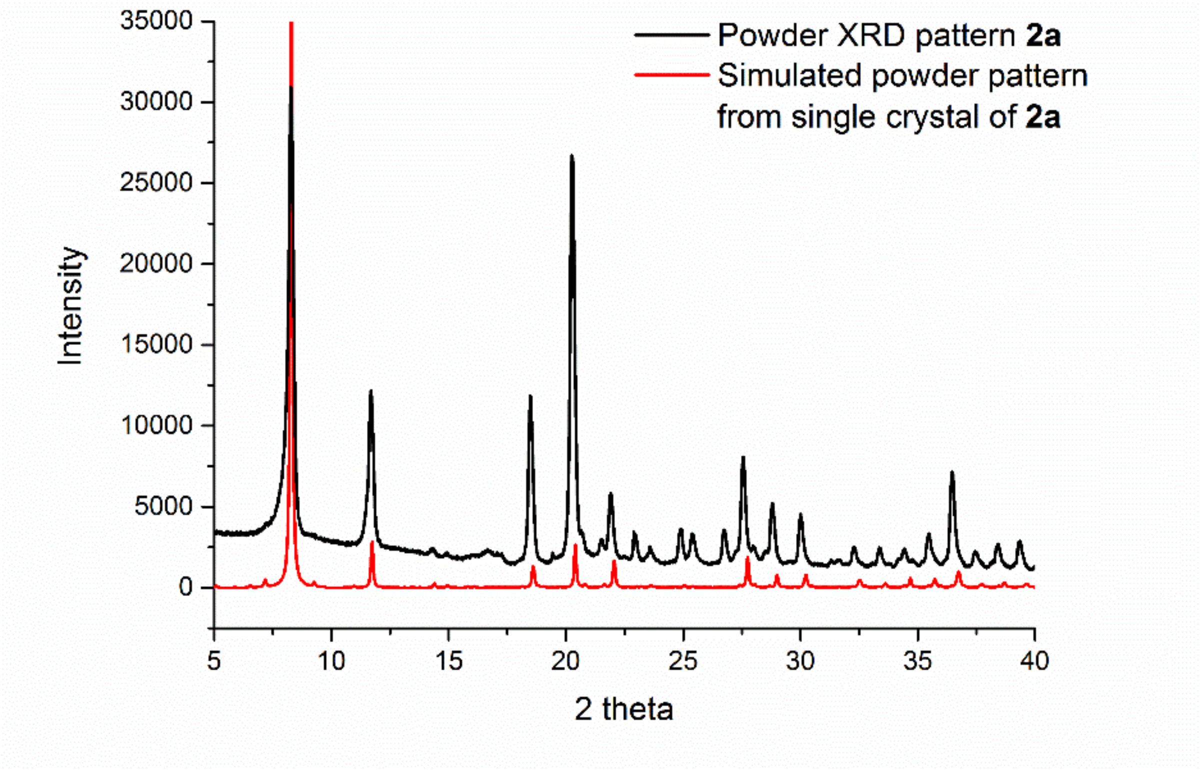

Supplementary Figure 10: Powder X-ray diffraction pattern of a bulk sample of **2A** compared to the simulated powder pattern extracted from single crystal diffraction data.

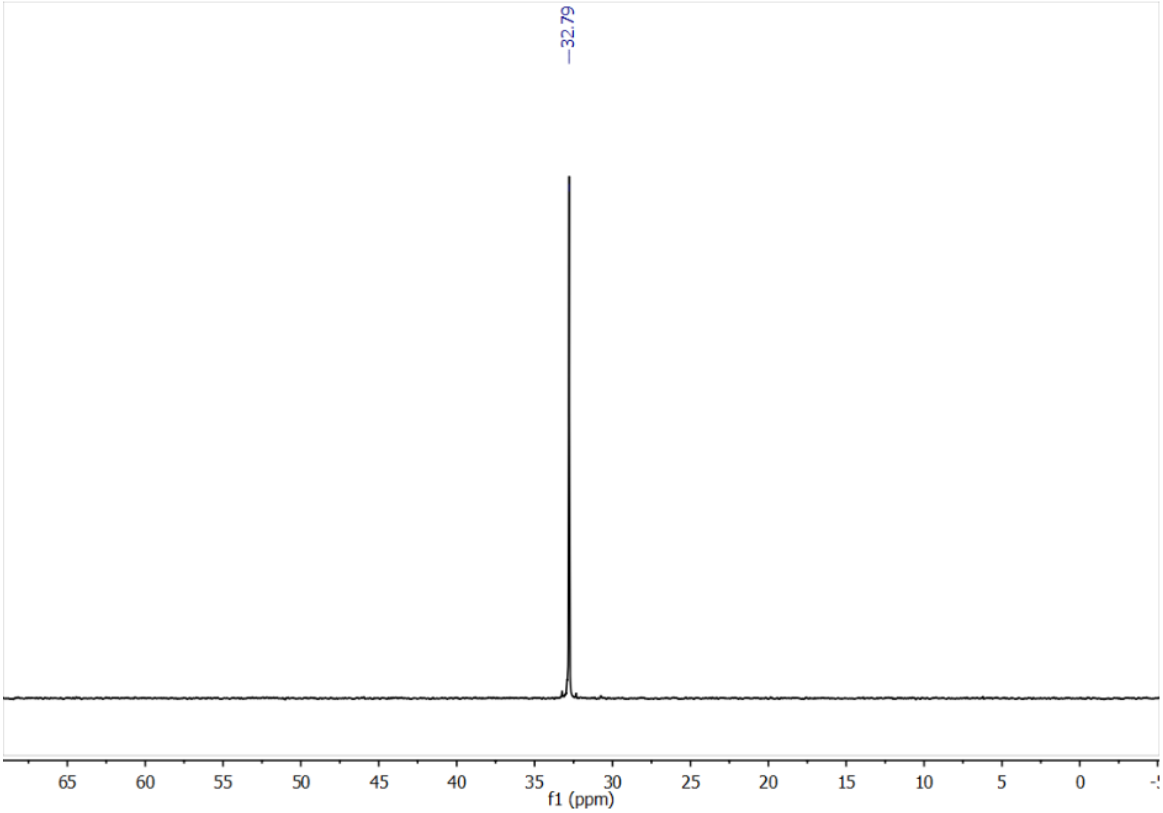

Supplementary Figure 11: $^{31}P\{^{1}H\}$ NMR spectrum of **2B**

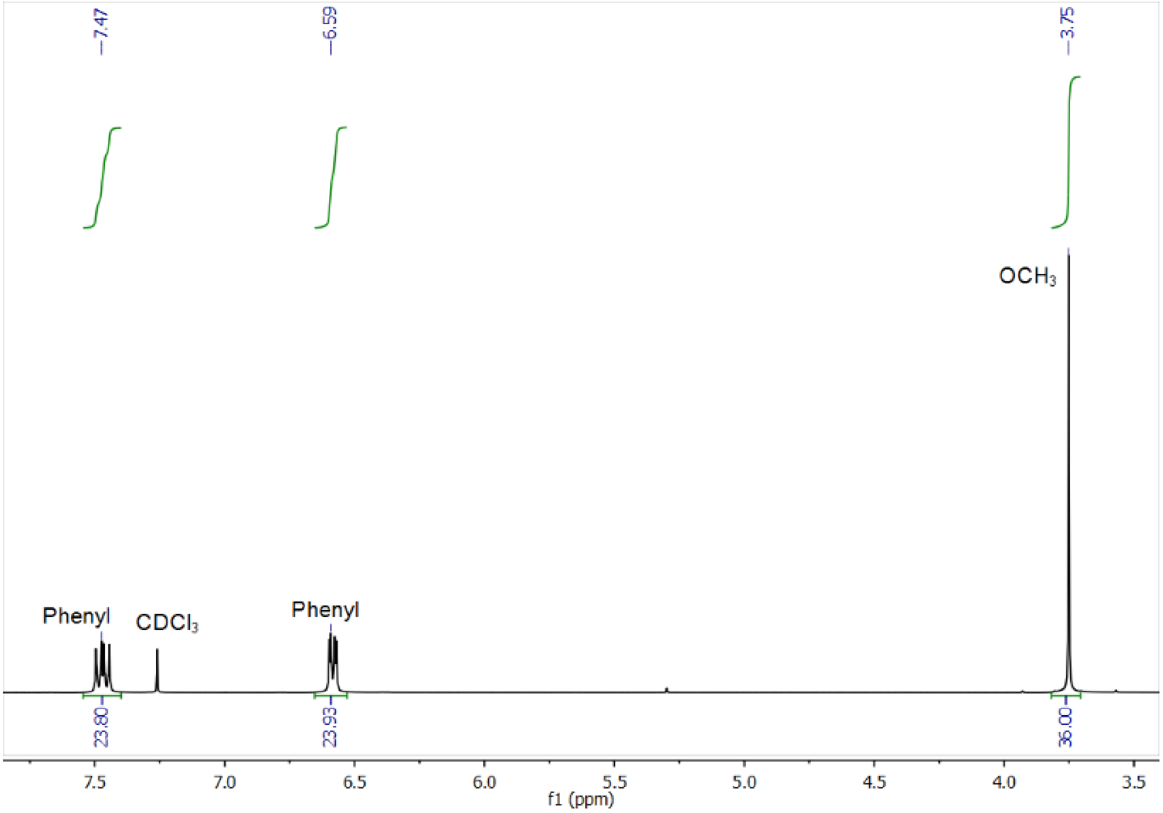

Supplementary Figure 12: ^{1}H NMR spectrum of **2B**

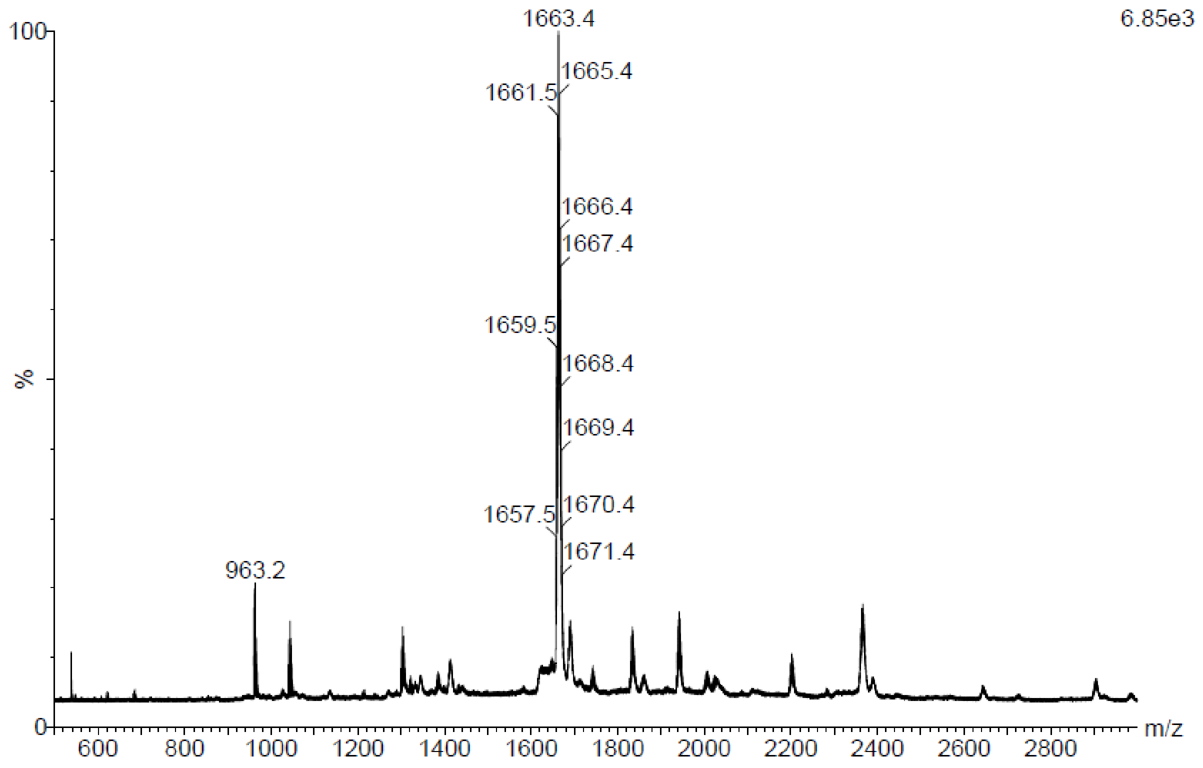

Supplementary Figure 13: MALDI-mass spectrum of **2B**

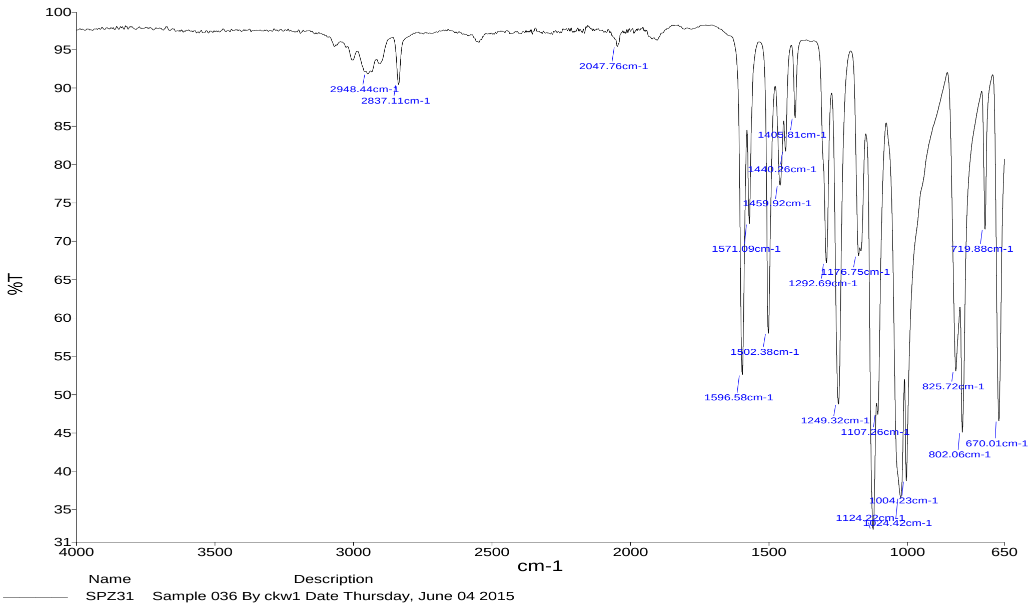

Supplementary Figure 14: I.R. spectrum of **2B**, selected stretches, ν: 2948.4 (w, aromatic C-H stretch), 2837.1 (w, CH_3 C-H stretch), 1124.2 (s, PO_2^- stretch), 1004.2 (PO_2^- stretch, s).

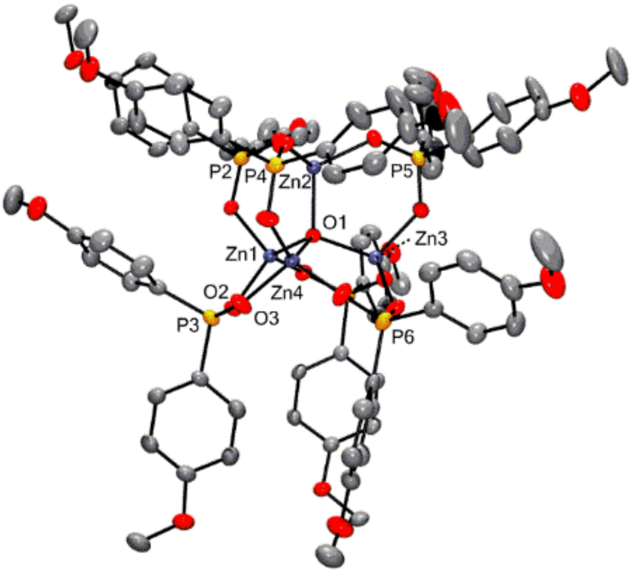

Supplementary Figure 15: Solid state structure of **2B**: Displacement ellipsoids at the 50% probability level, hydrogen atoms omitted for clarity. Selected bond lengths (Å) and angles (°): Zn1-O1, 1.996(2); Zn1-O4, 1.980(2); Zn1-O2, 1.960(2); Zn4-O3, 1.917(2); P3-O2, 1.513(2); P3-O3, 1.502(2); Z2-O1-Zn4, 106.92(9), Zn1-O1-Zn2, 111.62(10), O1-Zn1-O2, 115.69(9), O2-P3-O3, 114.36(13)

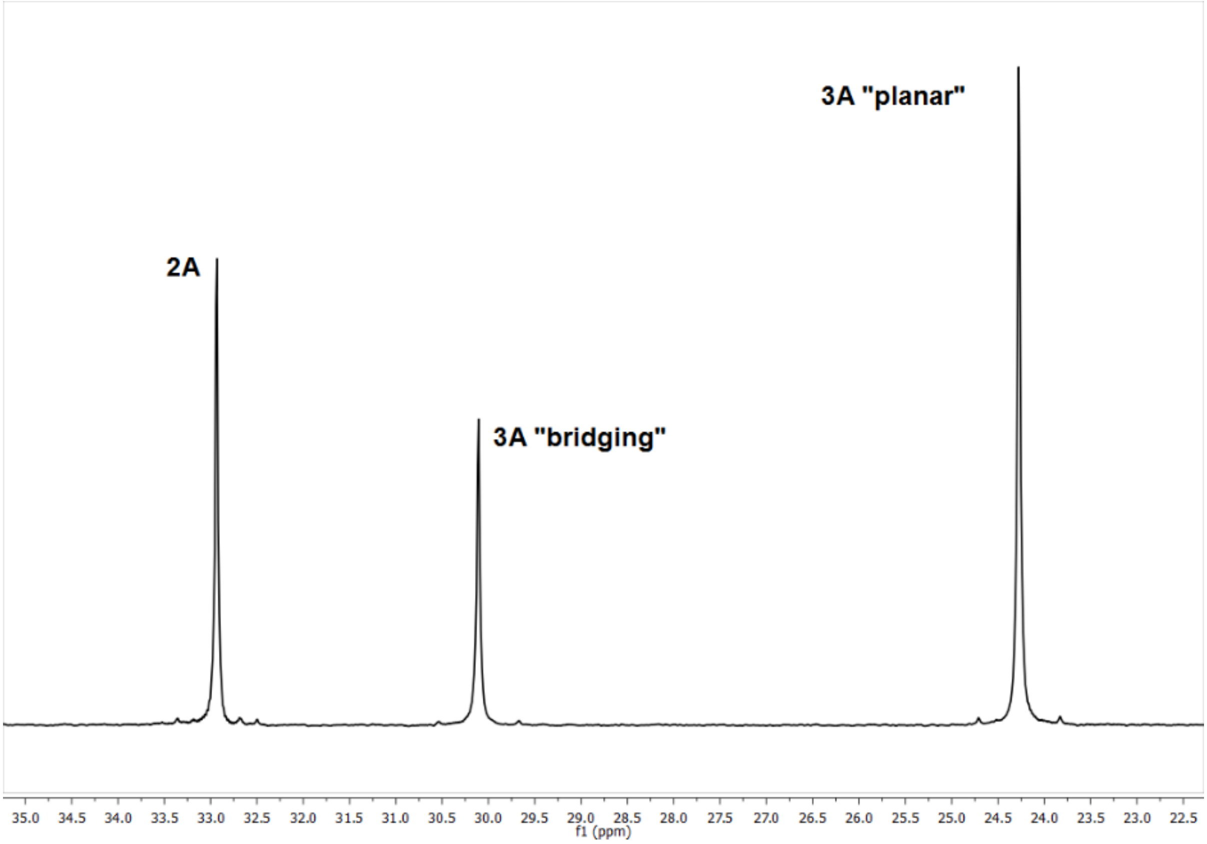

Supplementary Figure 16: $^{31}P\{^{1}H\}$ NMR spectrum of an equilibrium between **2A**, **3A** and water.

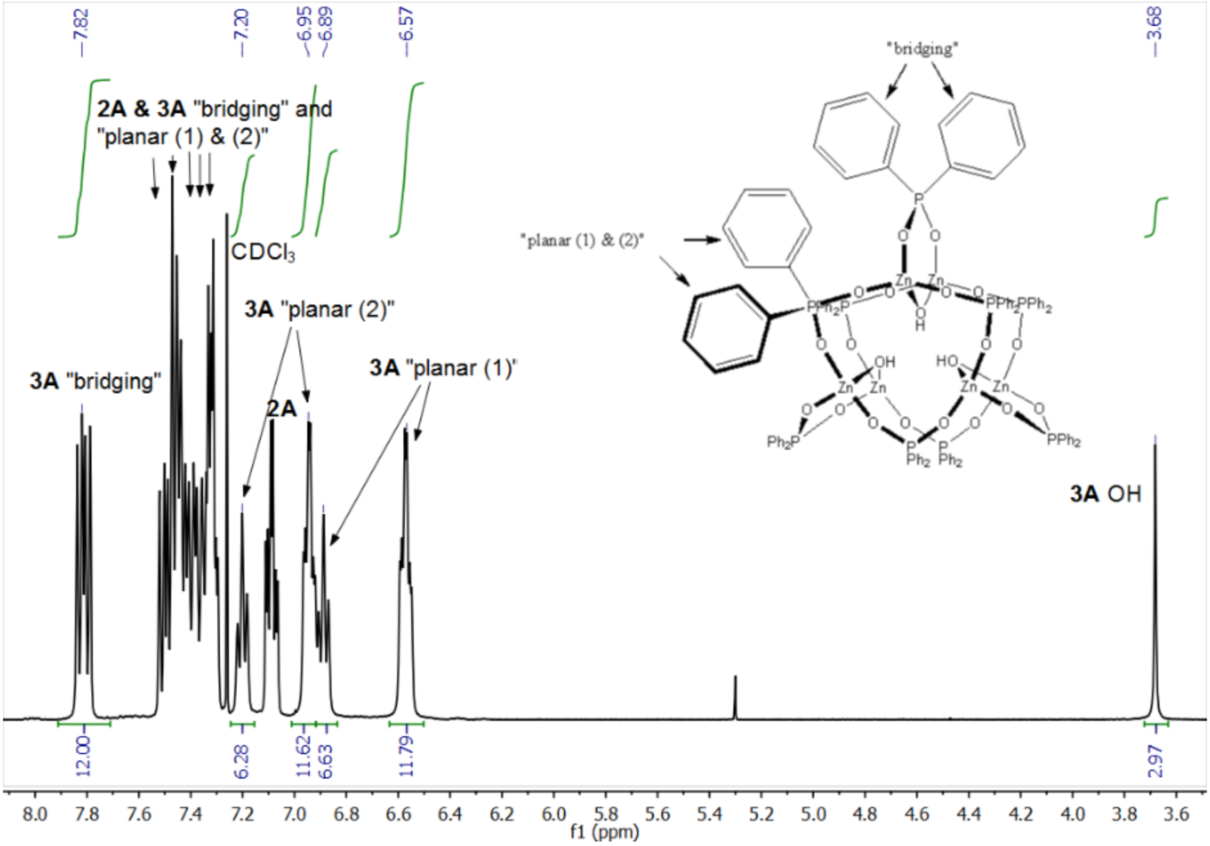

Supplementary Figure 17: ^{1}H NMR spectrum of an equilibrium between **2A**, **3A** and water. Diagram inset to aid assignment.

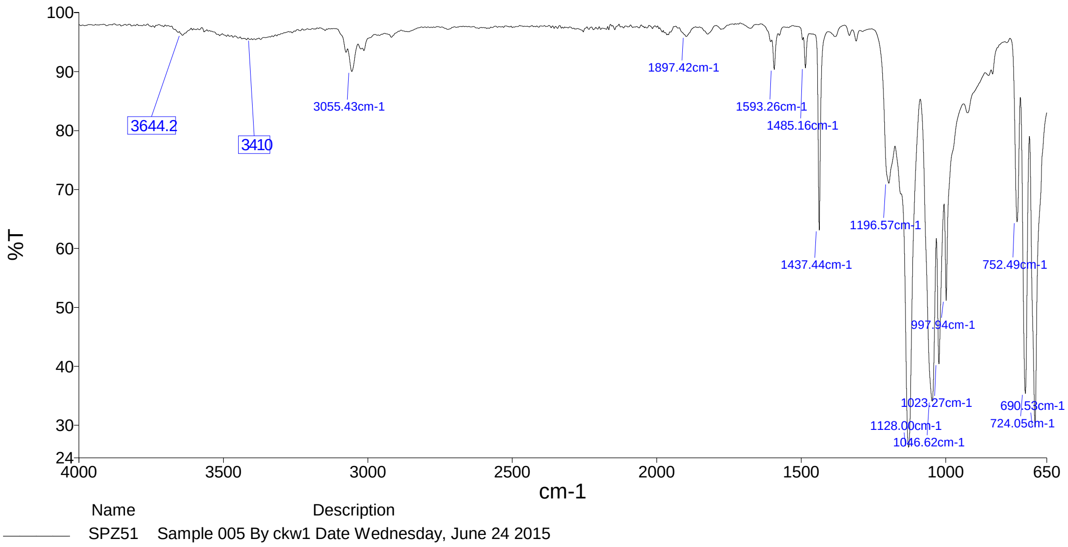

Supplementary Figure 18: I.R. spectrum of **3A**, selected stretches, ν: 3644, 3410 (w, O-H stretches), 3055.4 (w, C-H stretch), 1128.0 (s, PO_2^- stretch), 1046.6 (s, PO_2^- stretch).

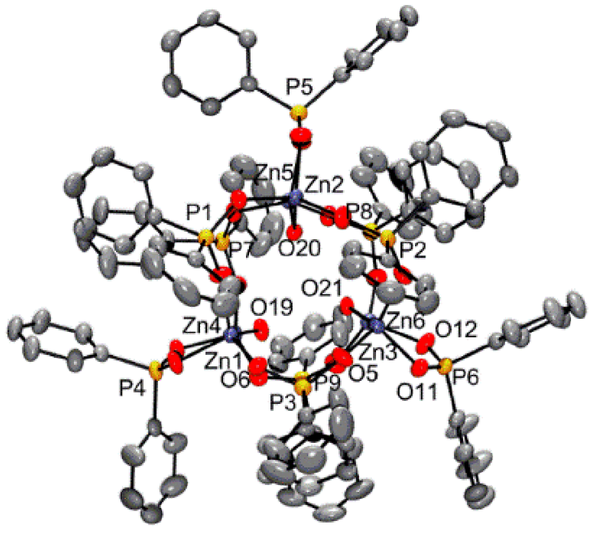

Supplementary Figure 19: Solid state structure of **3A**: Displacement ellipsoids at the 50% probability level, hydrogen atoms omitted for clarity. Selected bond lengths (Å) and angles (°): Zn1-O19, 1.908(2); Zn4-O19, 1.911(2); Zn2-O20, 1.956(2); Zn5-O20, 1.951(2); Zn3-O21, 1.945(2); Zn6-O21, 1.937(2); Zn1-O6, 1.925(3); Zn3-O5, 1.936(2); P3-O5, 1.502(3); P3-O6, 1.513(3); Zn3-O11, 1.966(2); Zn6-O12, 1.958(3); P6-O11, 1.496(3); P6-O12, 1.504(3); Zn1-O19-Zn4, 124.04(13); Zn2-O20-Zn5, 116.88(12); Zn3-O21-Zn6, 120.30(12); O5-P3-O6, 116.52(16); O11-P6-O12, 117.59(15)

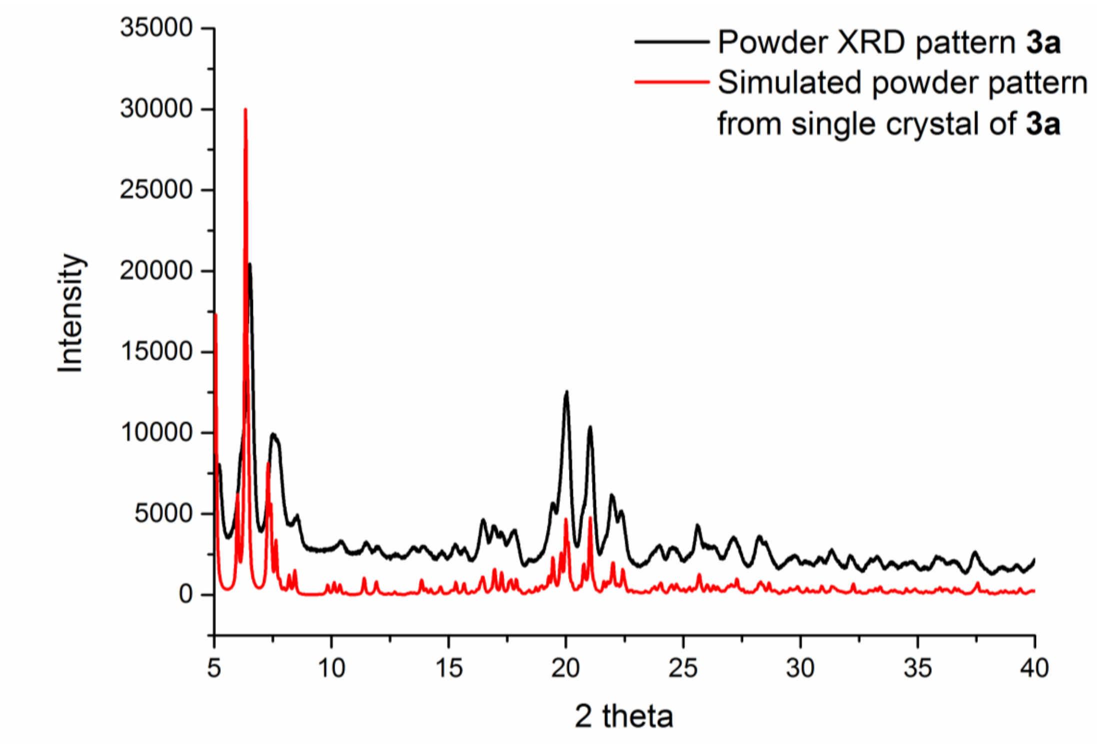

Supplementary Figure 20: Powder X-ray diffraction pattern of a bulk sample of **3A** compared to the simulated powder pattern extracted from single crystal diffraction data. No significant overlap with the pattern of **2A** is present.

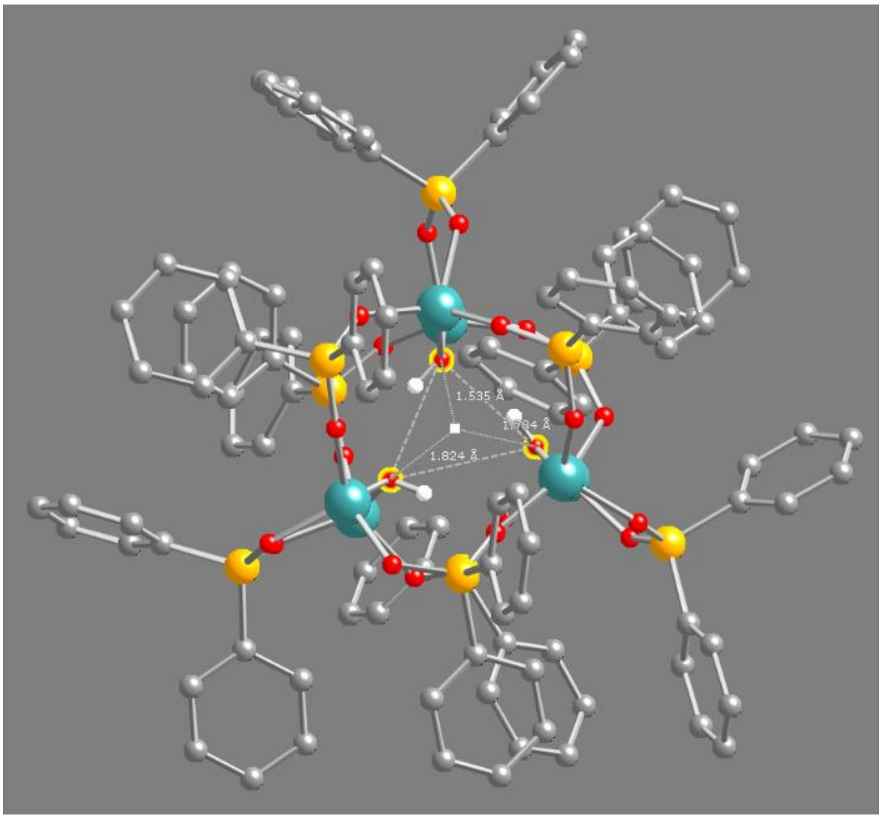

Supplementary Figure 21: Ball and stick model of **3A** showing the OH positions around a central point. O-centroid distances = 1.535, 1.784 and 1.824 Å. Hydrogen atoms other than hydoxides omitted for clarity.

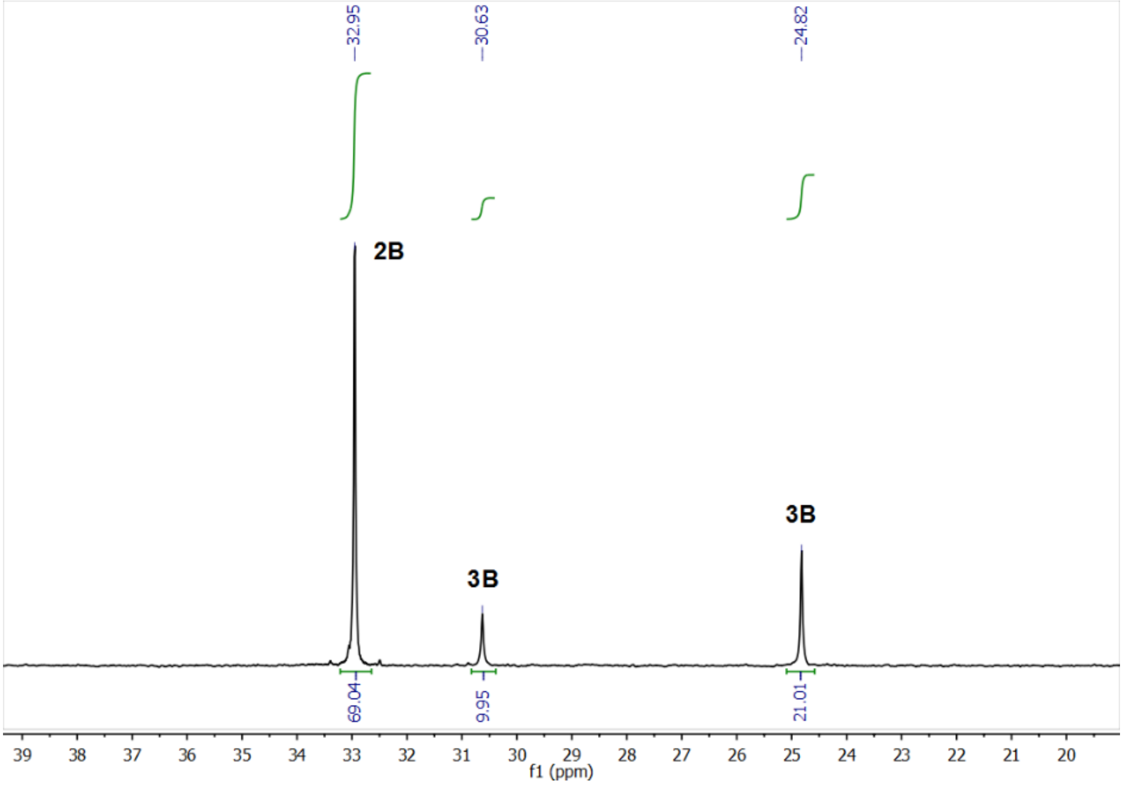

Supplementary Figure 22: $^{31}P\{^{1}H\}$ NMR spectrum of an equilibrium between **2B**, **3B** and water.

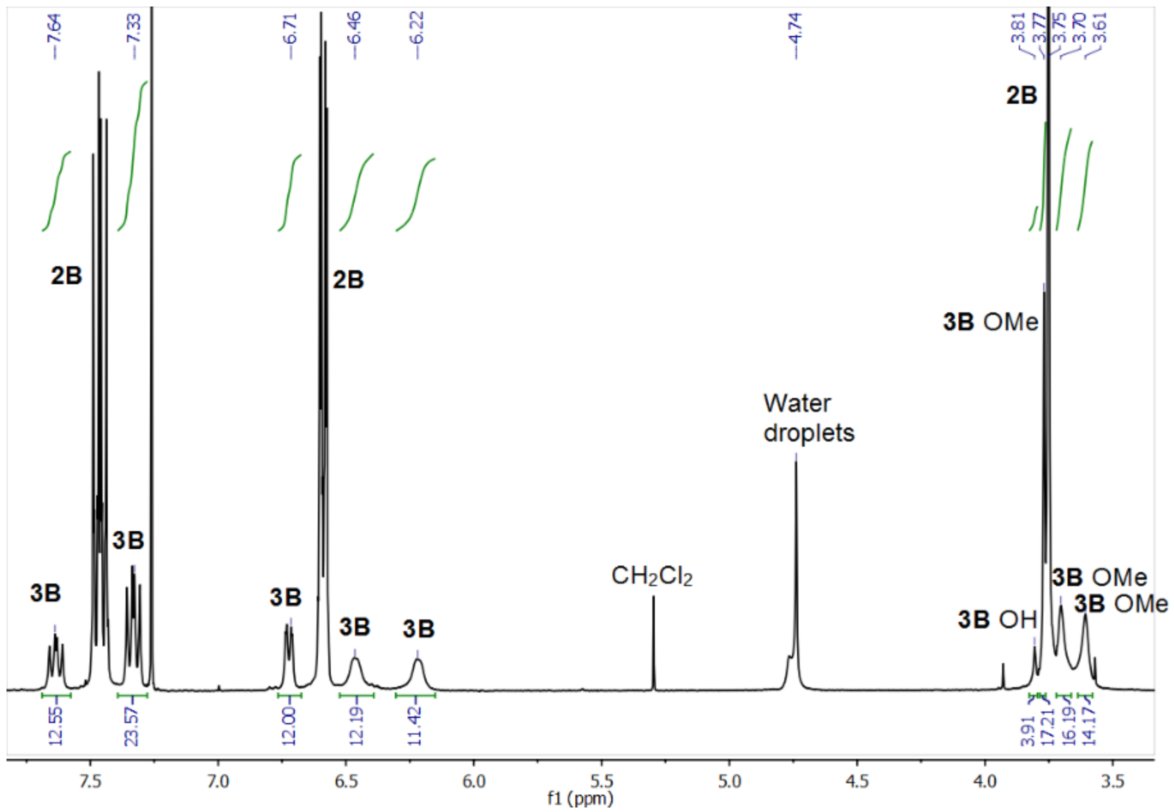

Supplementary Figure 23: ^{1}H NMR spectrum of an equilibrium between **2B**, **3B** and water.

Variable Temperature NMR spectra (2A/3A)

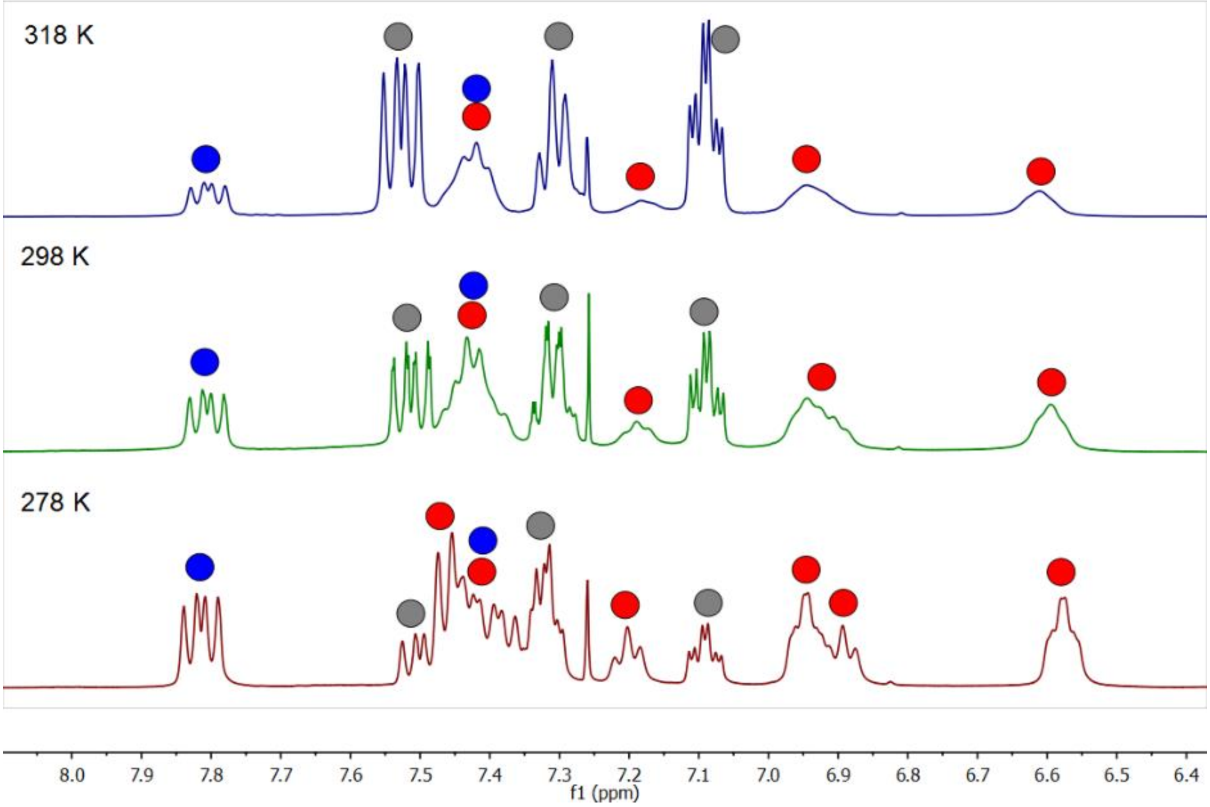

Supplementary Figure 24: Variable temperature ^{1}H NMR spectra of **3A** (red and blue circles) in equilibrium with **2A** (grey circles). For **3A**, red circles are assigned to phenyl resonances from asymmetric ("planar") phosphinate ligands, whilst blue circles are assigned to resonances from symmetric ("bridging") phosphinate ligands. At 273 K the phenyl resonances for **3A** are all sharp suggesting a relatively static species, however at 298 K the two phenyl environments attributed to the "planar" DPPA units broaden suggesting that these DPPA ligands may rotate, exchanging the phenyl positions, presumably via (partial) dissociation. The hydroxide signal of **3A** remains sharp across all temperatures and there is no evidence of the "bridging" DPPA units interchanging positions with the "planar" positions even at higher temperatures (up to 328 K). ^{1}H NMR Spectra of **3B** (288 K-328 K) and **4A** (298 K) also show broadening exclusively in asymmetric phosphinate environments. These observations suggest a consistent ligand coordination mode exists in these clusters, with fast rotation of the phosphinate relative to the NMR timescale at room temperature, but exchange between different phosphinate environments not observed upon the same timescale.

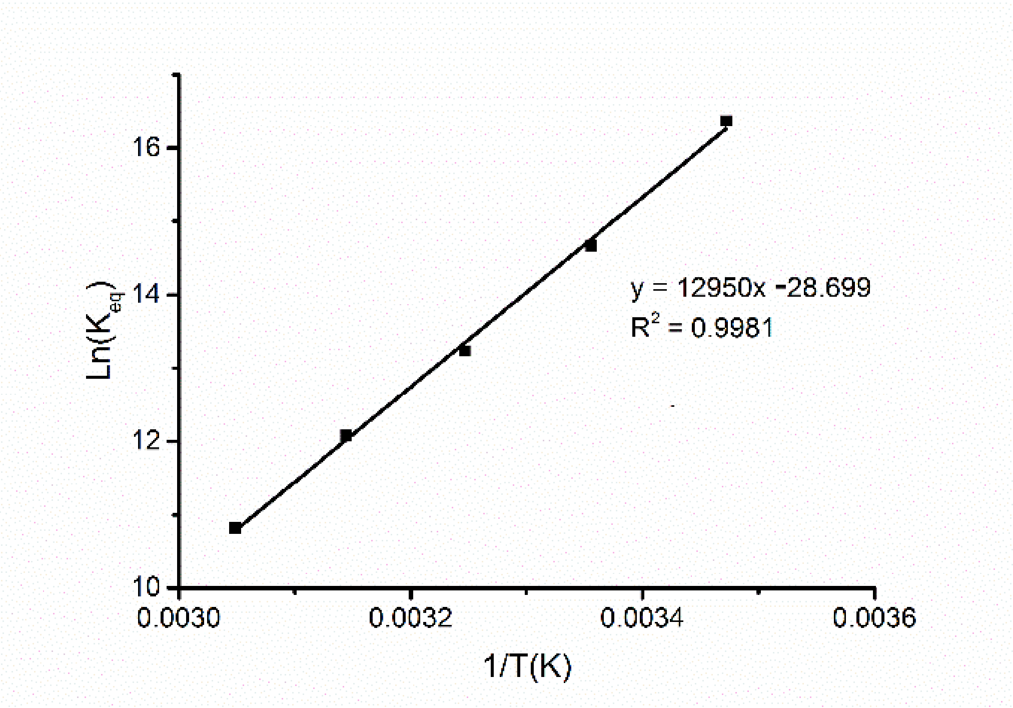

Supplementary Figure 25: Van't Hoff plot for the equilibrium between **2A**, **3A** and water. See Supporting Methods.

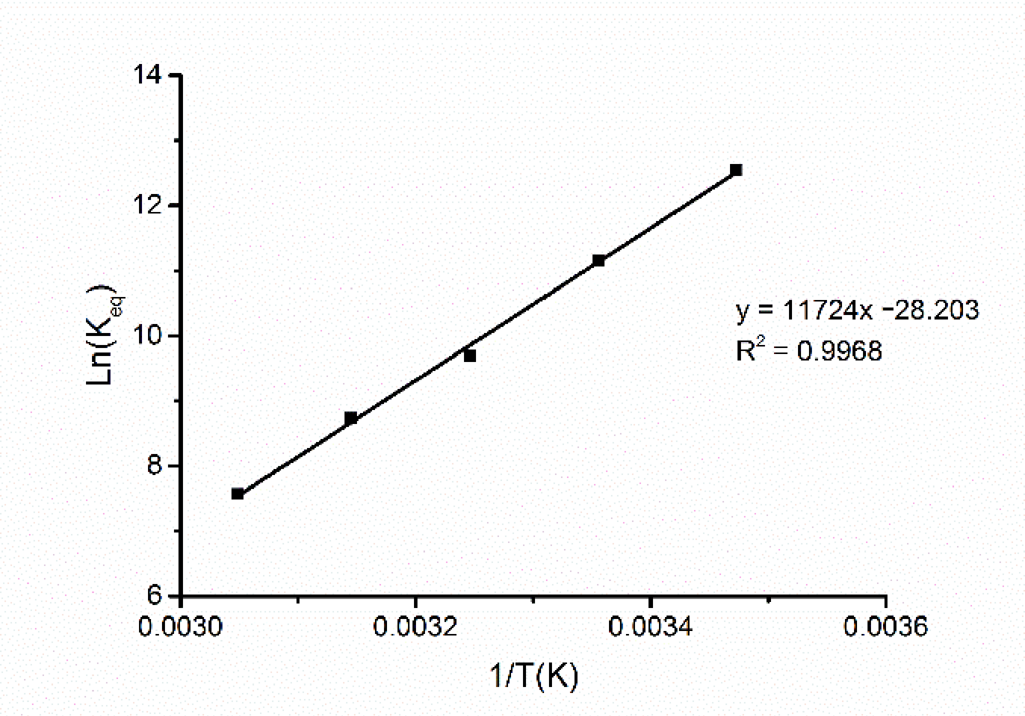

Supplementary Figure 26: Van't Hoff plot for the equilibrium between **2B**, **3B** and water. See Supporting Methods.

Spectra and crystal structures of compounds 4A and 5A

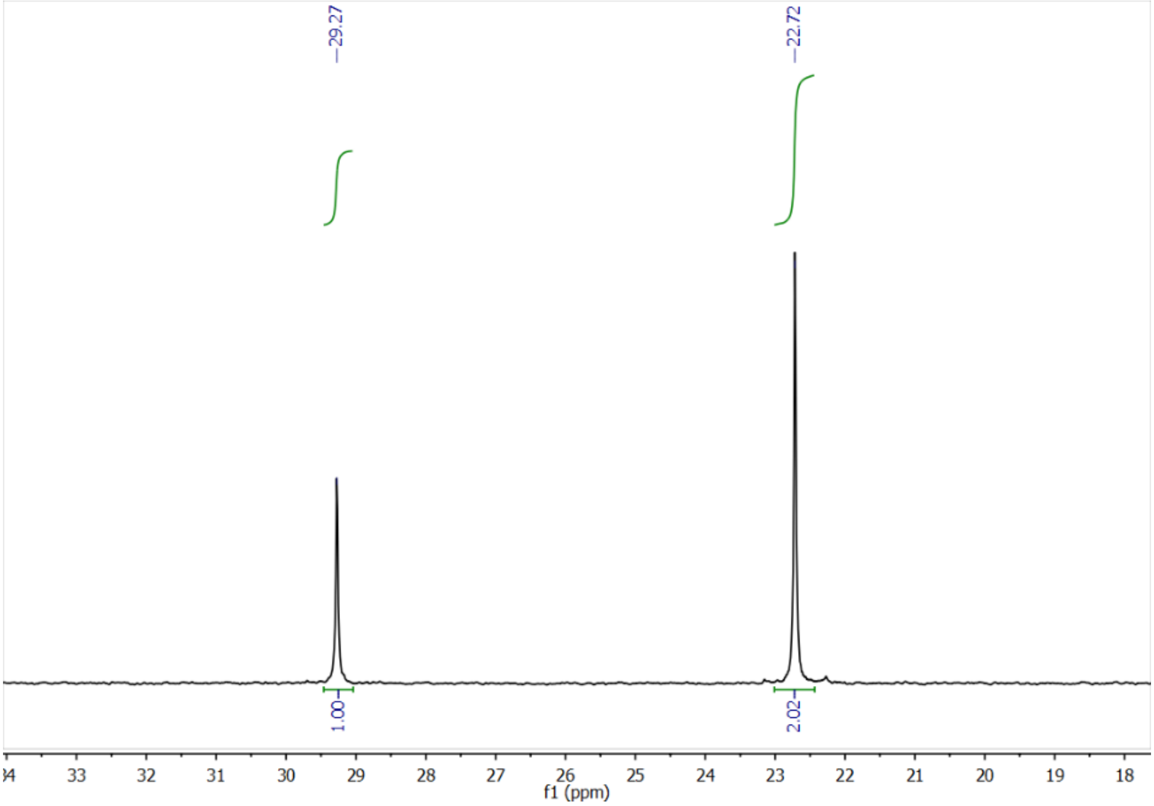

Supplementary Figure 27: $^{31}P\{^{1}H\}$ NMR spectrum of **4A**

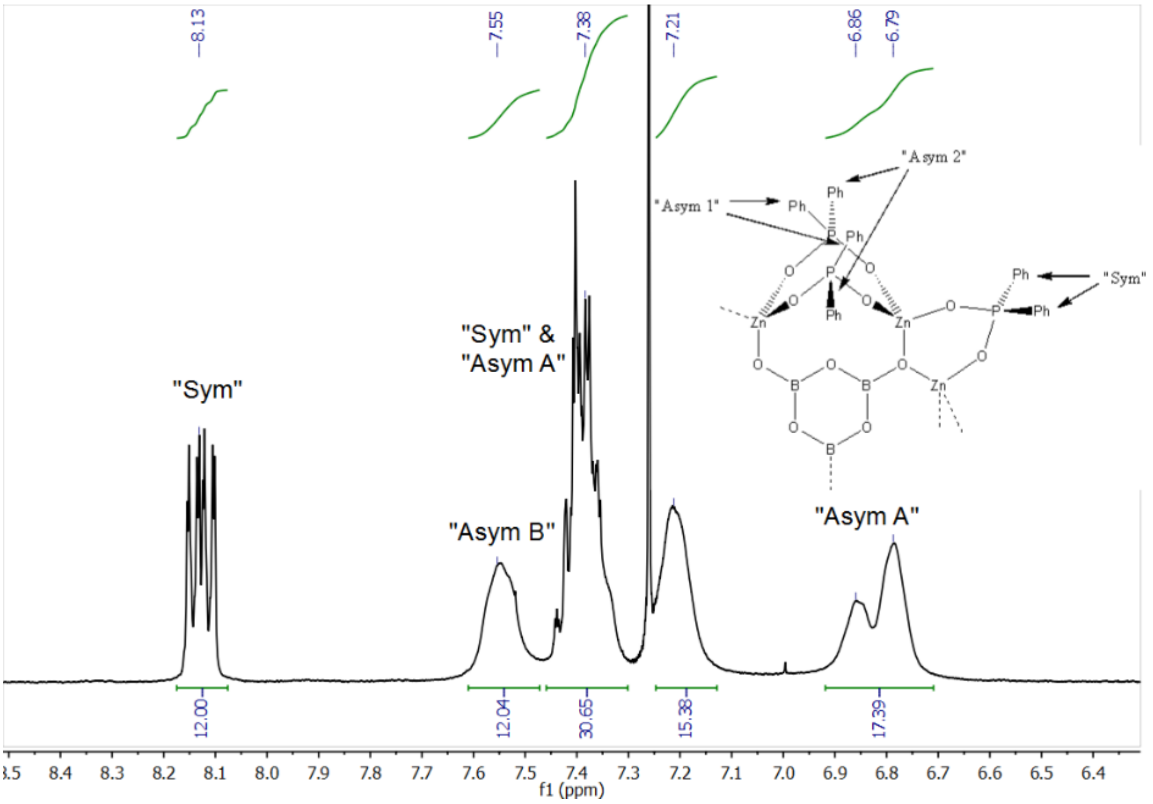

Supplementary Figure 28: ^{1}H NMR spectrum of **4A.** Sym and both Asym envrironments shown inset. Sets of Asym A, Asym B and Sym resonances couple to each other in ^{1}H-^{1}H COSY spectra.

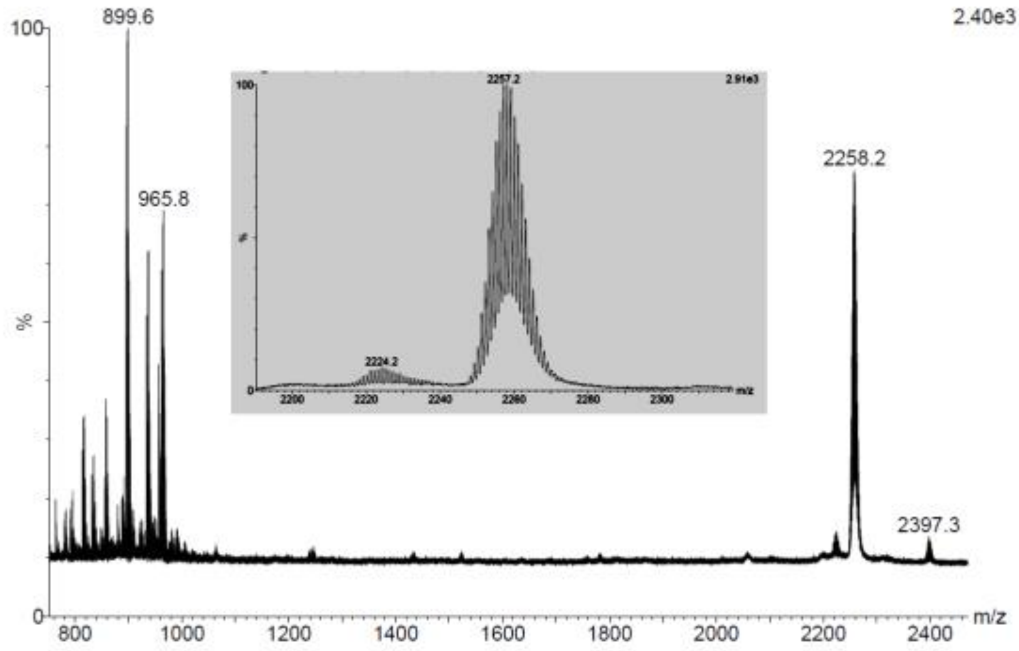

Supplementary Figure 29: MALDI-mass spectrum of **4A**; $[Zn_6(BO_2)_3(DPPA)_8]^+$ signal inset.

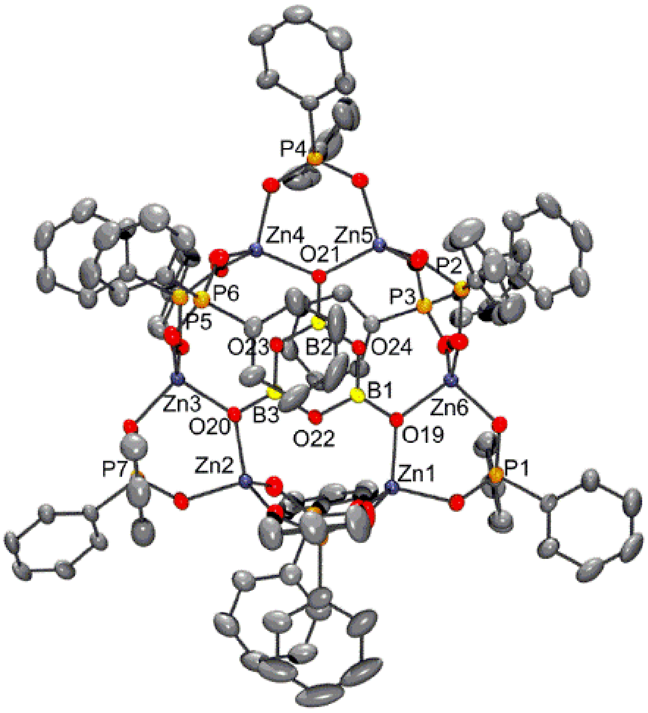

Supplementary Figure 30: Solid state structure of one independent molecule of **4A**: Displacement ellipsoids at the 50% probability level, hydrogen atoms omitted for clarity. Selected bond lengths (Å) and angles (°): **4A**) boroxine ring B-O, 1.376(4)-1.387(4); B1-O19, 1.333(4); Zn1-O19, 1.9614(19); Zn1-O1, 1.9424(19); Zn1-O16, 1.921(2); O22-B1-O24, 119.2(3); O22-B1-O19, 120.4(3); Zn1-O19-Zn6, 129.37(10); O1-Zn1-O19, 99.94(8); O16-Zn1-O19, 112.56(11)

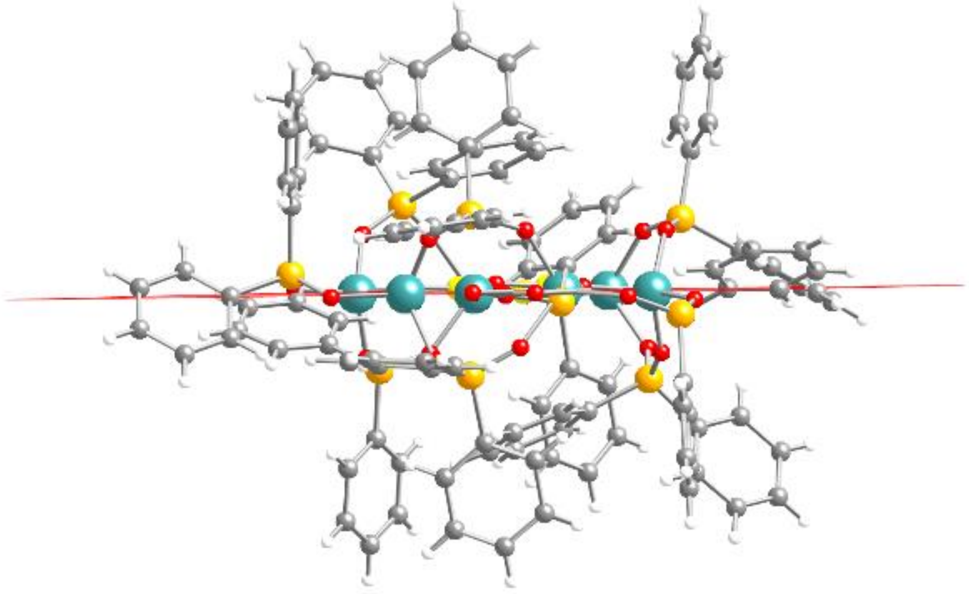

Supplementary Figure 31: Solid state structure of one independent molecule of **4A** showing planarity through six zinc atoms.

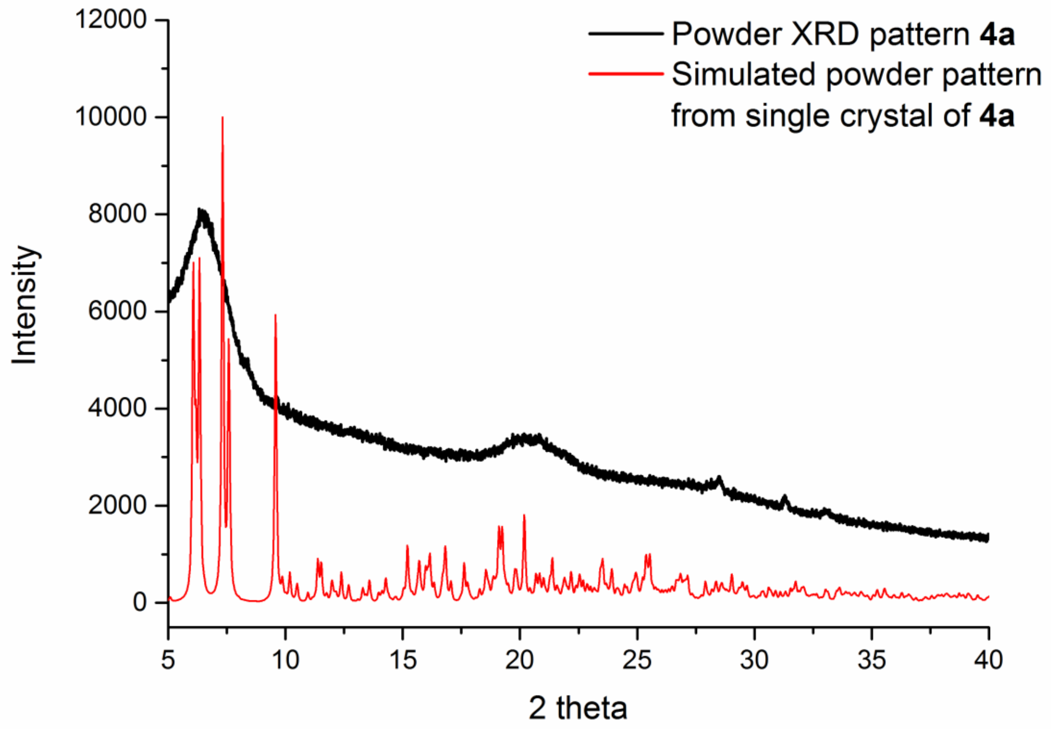

Supplementary Figure 32: Powder X-ray diffraction pattern of a bulk sample of **4A** obtained by fast precipitation compared to the simulated powder pattern extracted from single crystal diffraction data. It appears the bulk phase is mainly amorphous without using slow crystallisation techniques (N.B. the sample was fully soluble in $CDCl_3$ and clean by ^{1}H & ^{31}P NMR spectroscopy).

Equilibrium between **4A**, **2A** and **3A**:

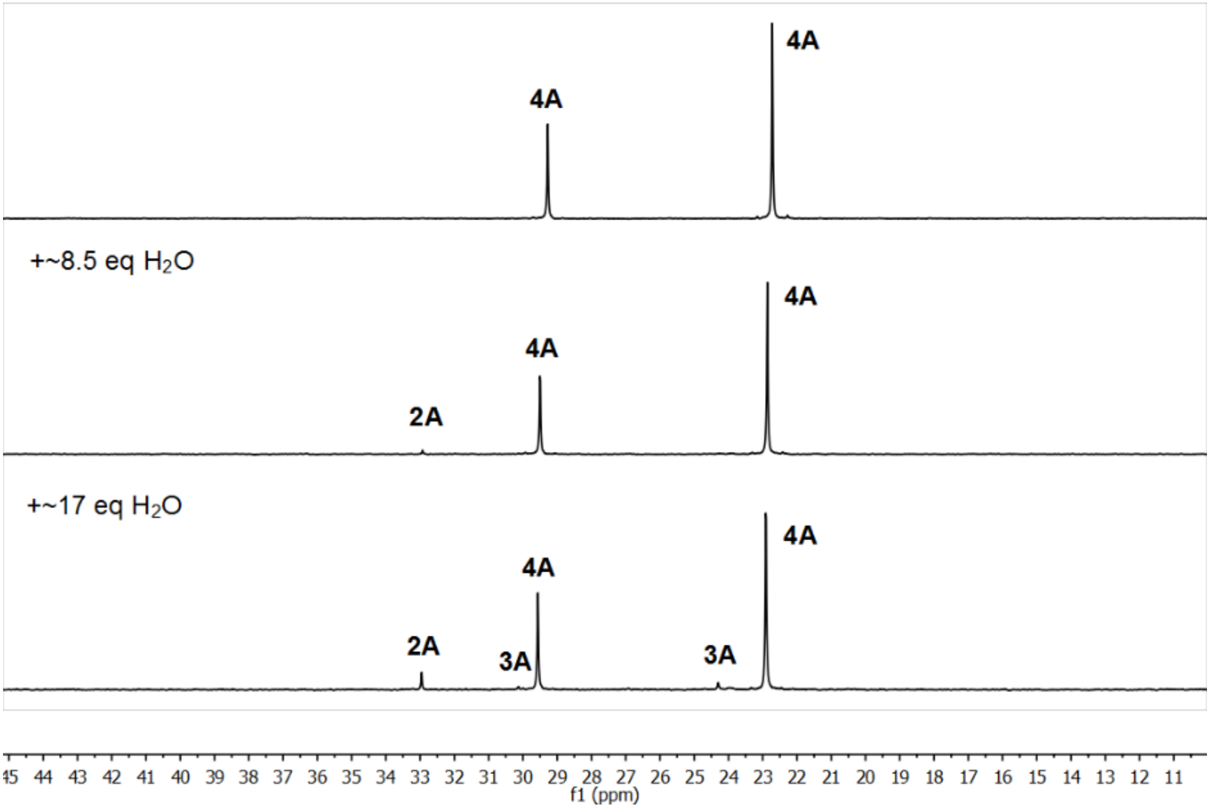

Supplementary Figure 33: $^{31}P\{^1H\}$ NMR spectra of a $CDCl_3$ solution of **4A** with added water. **4A** (8 mg, 3.3 µmol) was dissolved in $CDCl_3$ (0.5 mL) and a $^{31}P\{^1H\}$ NMR spectrum recorded. To this NMR tube, water (0.5 µL, 28 µmol) was added and the spectrum repeated, this revealed the growth of a trace of **2A** (molar ratio: 98:2 in favour of **4A**). A further portion of water (0.5 µL, 28 µmol) was added revealing increased growth of signals attributed to **2A** and the observation of its known equilibrium partner **3A** (molar ratio: 89:7:4 for **4A**:**2A**:**3A**). The data show that an equilibrium exists between **4A**, **2A** and **3A** in the presence of water, however **4A** is strongly favoured even with multiple equivalents of water present.

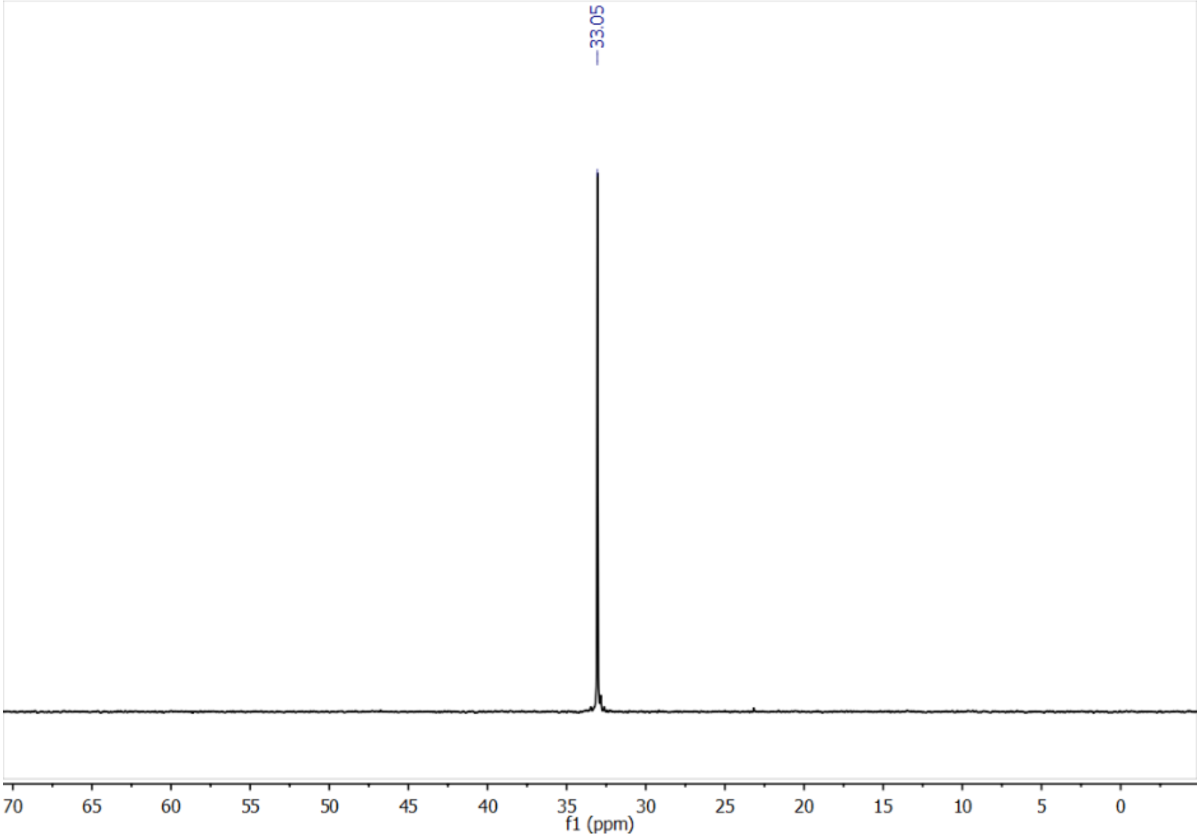

Supplementary Figure 34: $^{31}P\{^{1}H\}$ NMR spectrum of **5A**

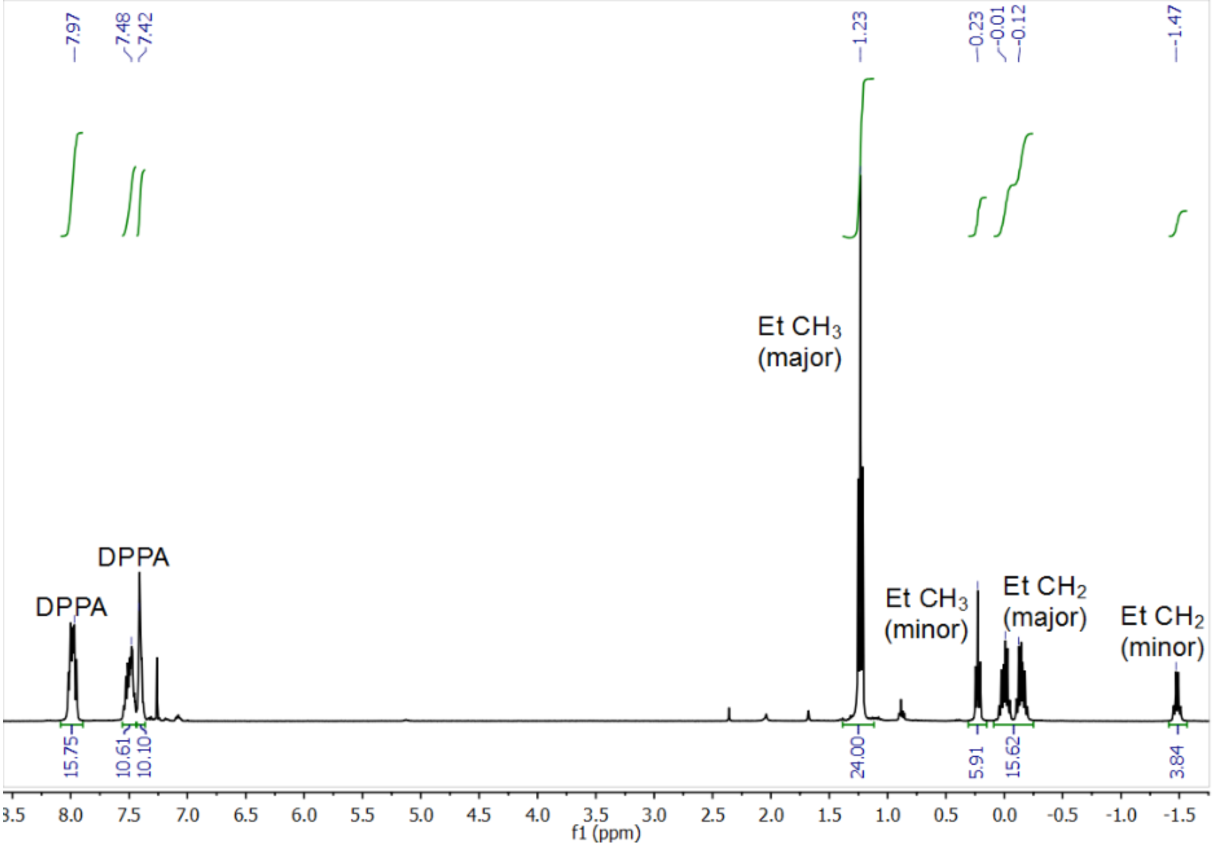

Supplementary Figure 35: ^{1}H NMR spectrum of **5A**

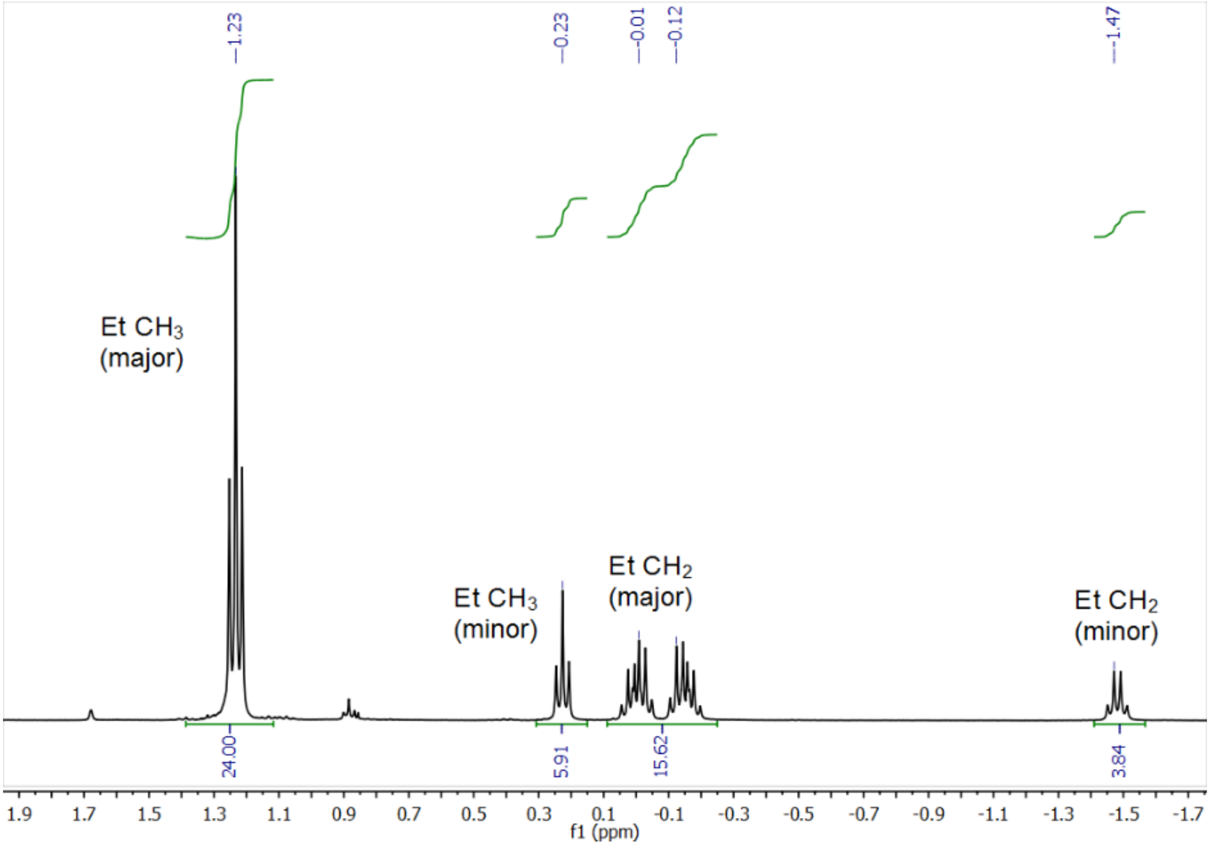

Supplementary Figure 36: ^{1}H NMR spectrum of **5A**, ethyl region only

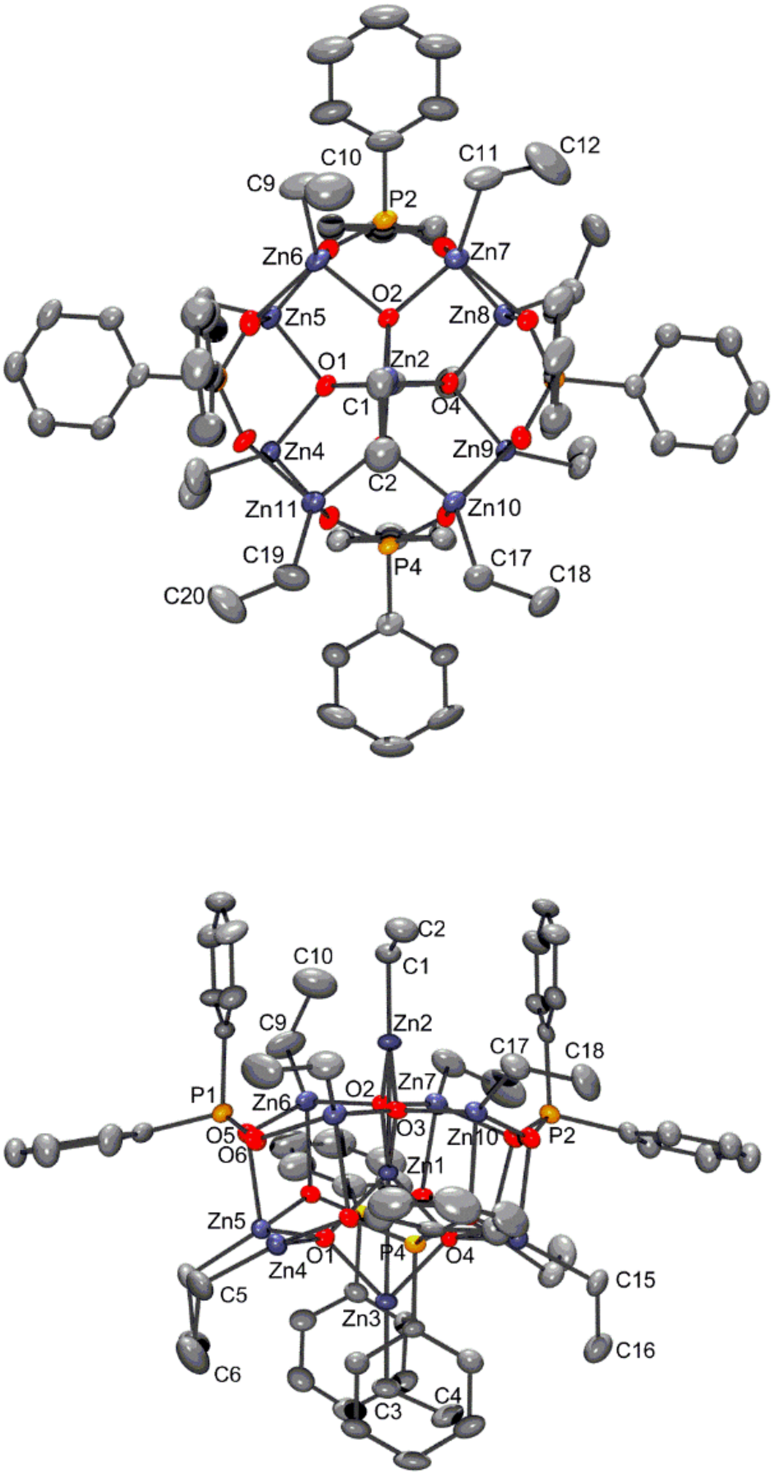

Supplementary Figure 37: Solid state structures of **5A** (2 views shown): Displacement ellipsoids at the 50% probability level, hydrogen atoms omitted for clarity. Selected bond lengths (Å) and angles (°): **5A**) Zn1-O1, 2.003(4); Zn3-O1, 1.985(4); Zn5-O1, 1.964(4); Zn2-C1, 1.948(6); Zn4-C5, 1.959(7); Zn4-O6, 2.286(4), O1-Zn1-O2, 122.48(14); O1-Zn3-O4, 87.96(15); O1-Zn3-C3, 136.8(2)

NMR Study of the Equilibrium between 1A/B, 2A/B, 5A/B and $ZnEt_2$

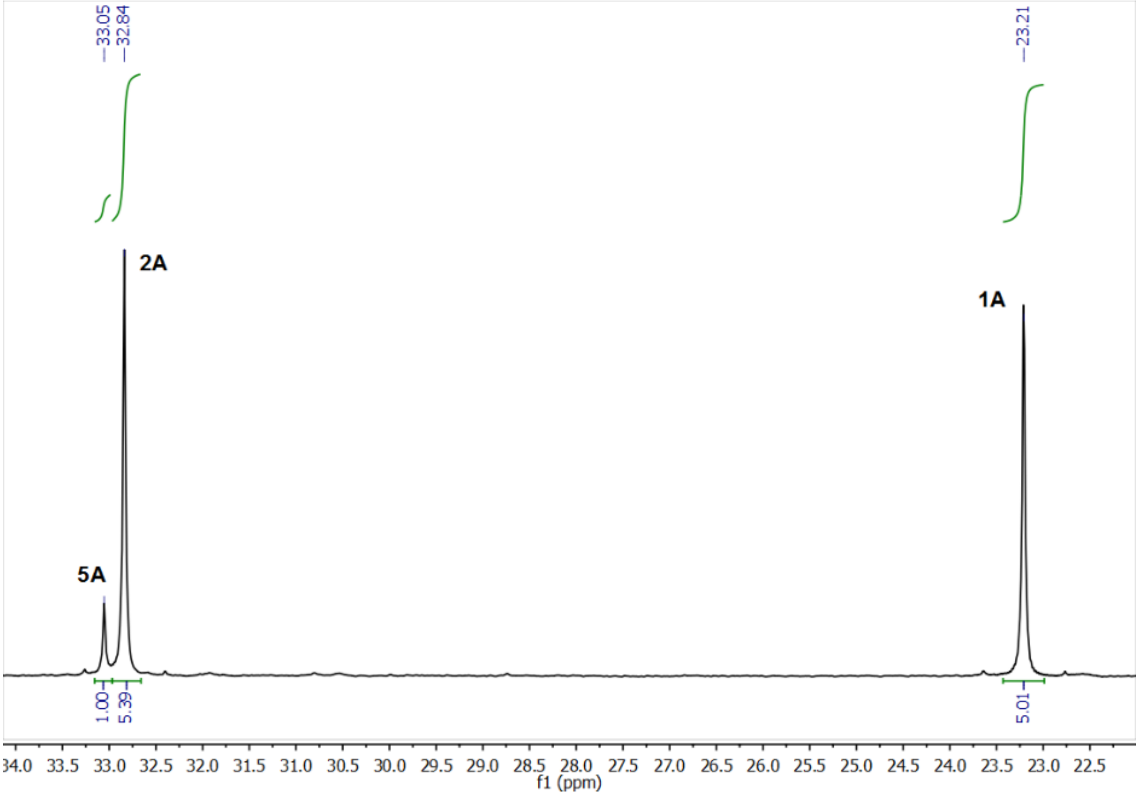

Supplementary Figure 38: $^{31}P\{^1H\}$ NMR spectrum of **2A** + 15 $ZnEt_2$. See Supporting Methods.

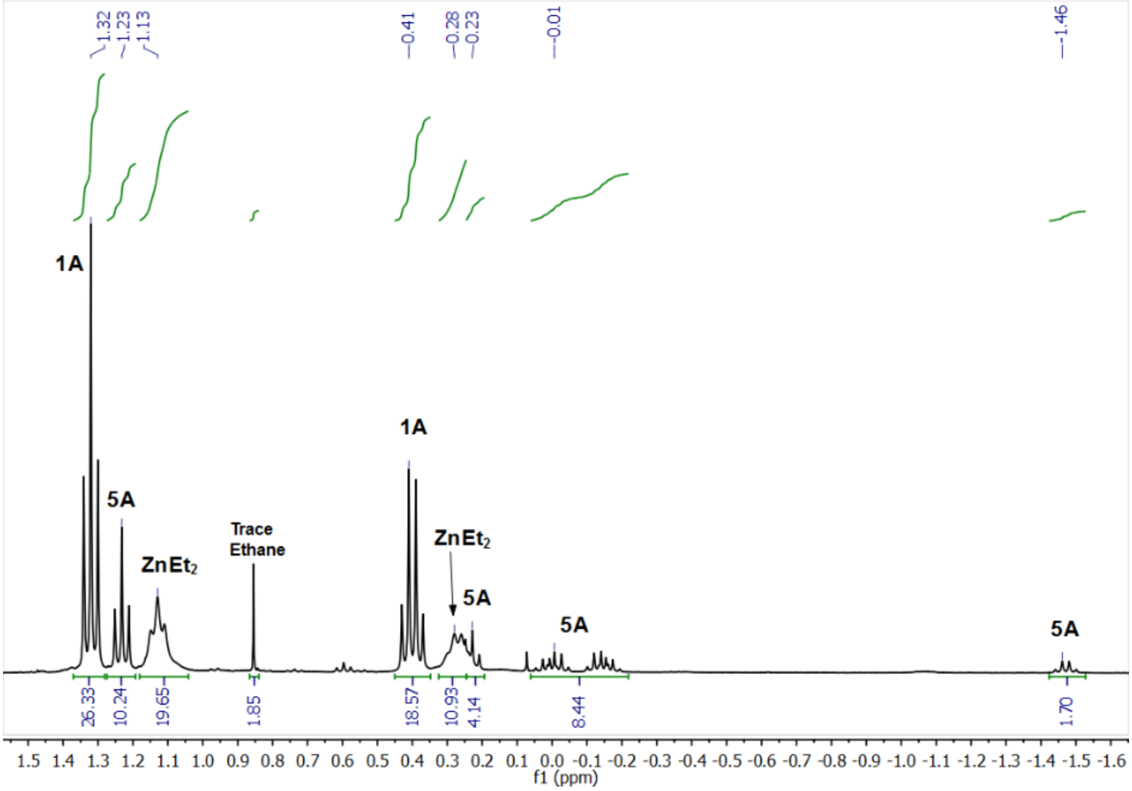

Supplementary Figure 39: ^1H NMR spectrum (ethyl region) of 4 **2A** + 15 $ZnEt_2$. See Supporting Methods.

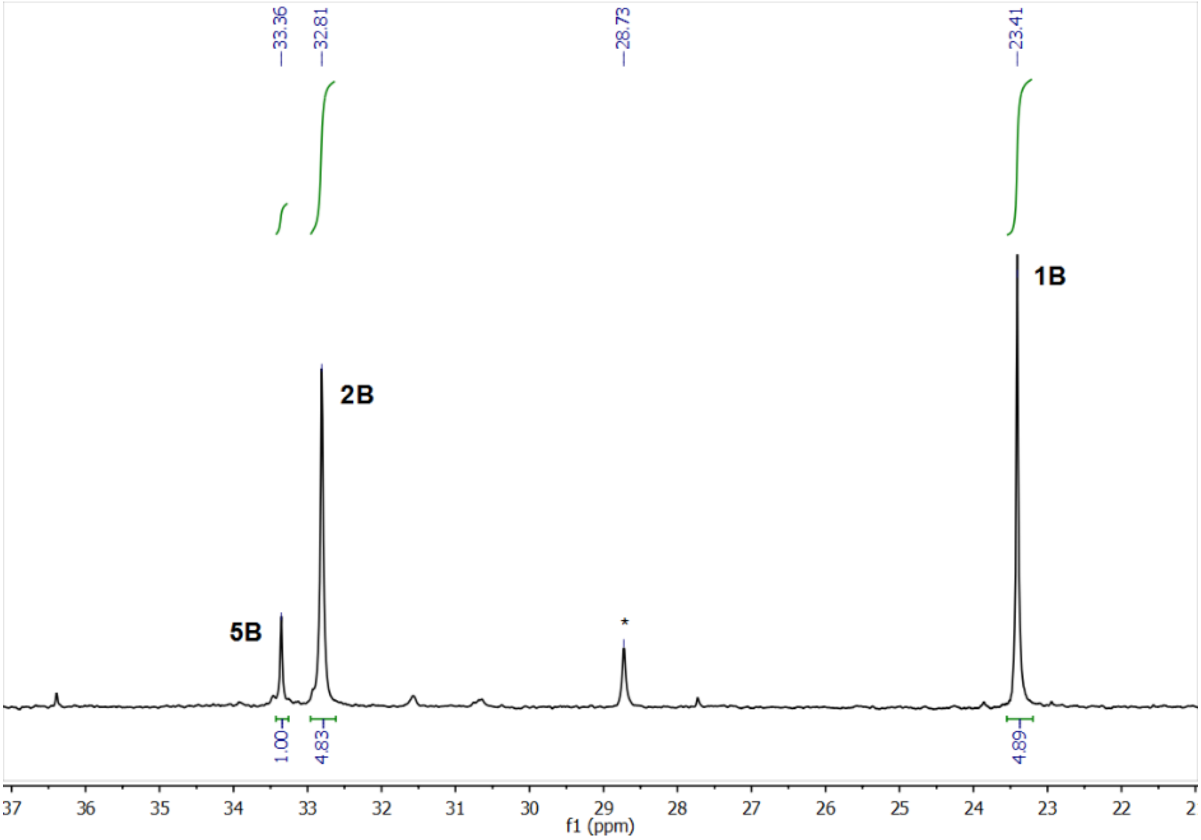

Supplementary Figure 40: $^{31}P\{^{1}H\}$ NMR spectrum of **2B** + 15 $ZnEt_2$. Unknown species marked with *.

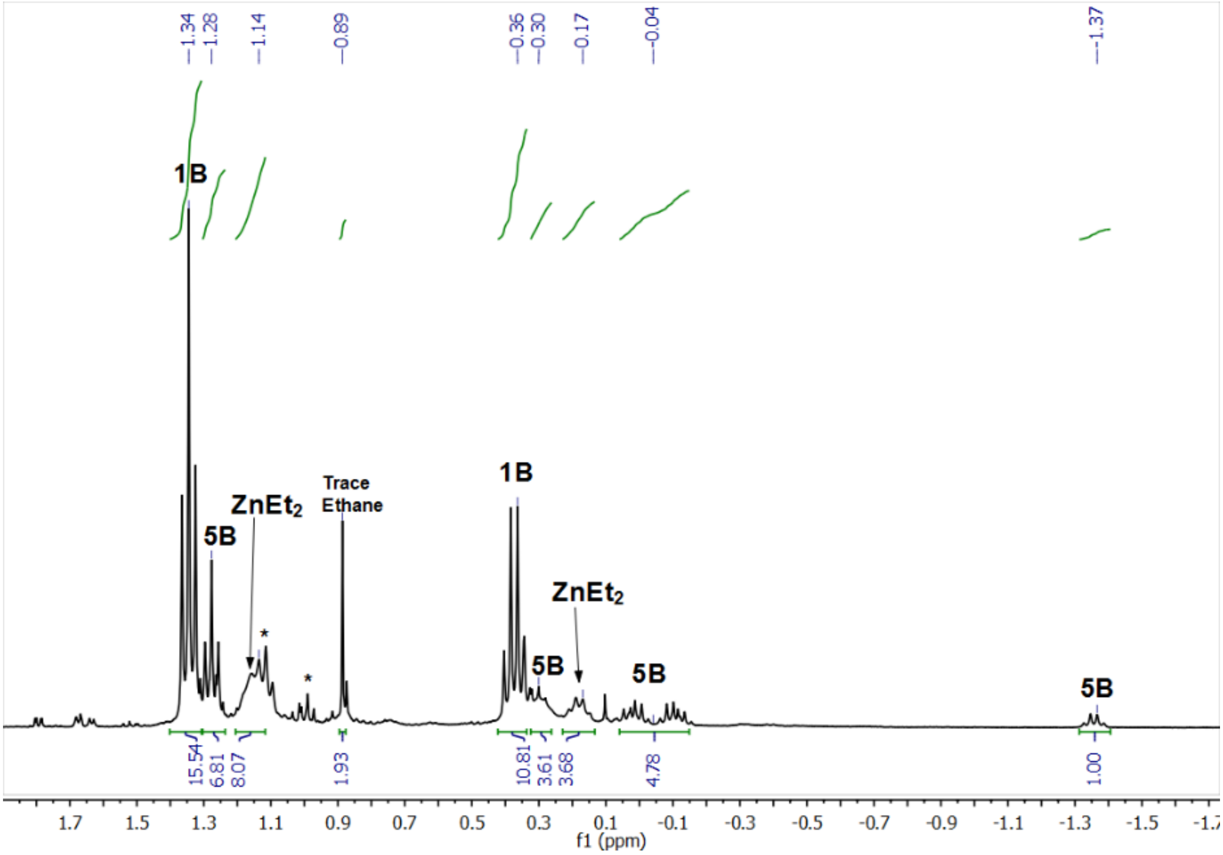

Supplementary Figure 41: ^{1}H NMR spectrum (ethyl region) of 4 **2B** + 15 $ZnEt_2$. *Unknown species.

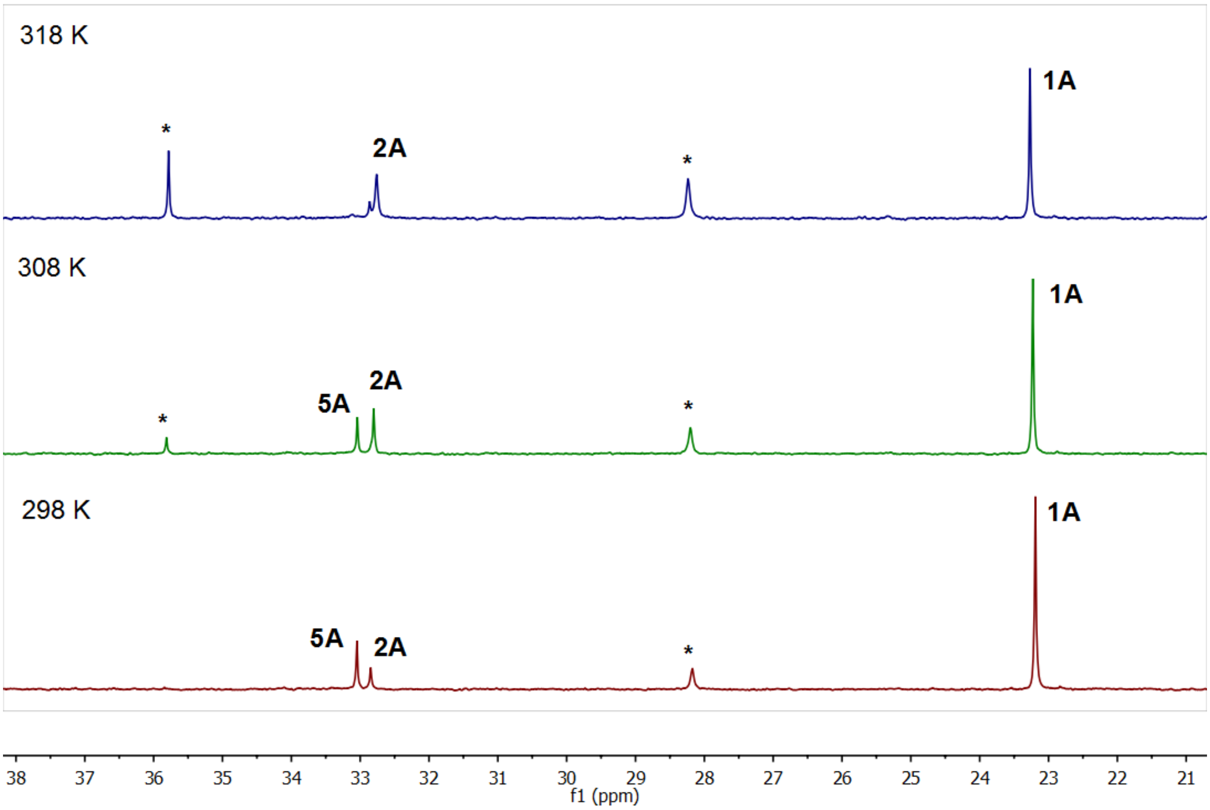

Supplementary Figure 42: ^{31}P{^{1}H} NMR spectrum of 4 **2A** + 15 $ZnEt_2$ at three different temperatures. * marks unknown species which grow in over time (likely decomposition products).

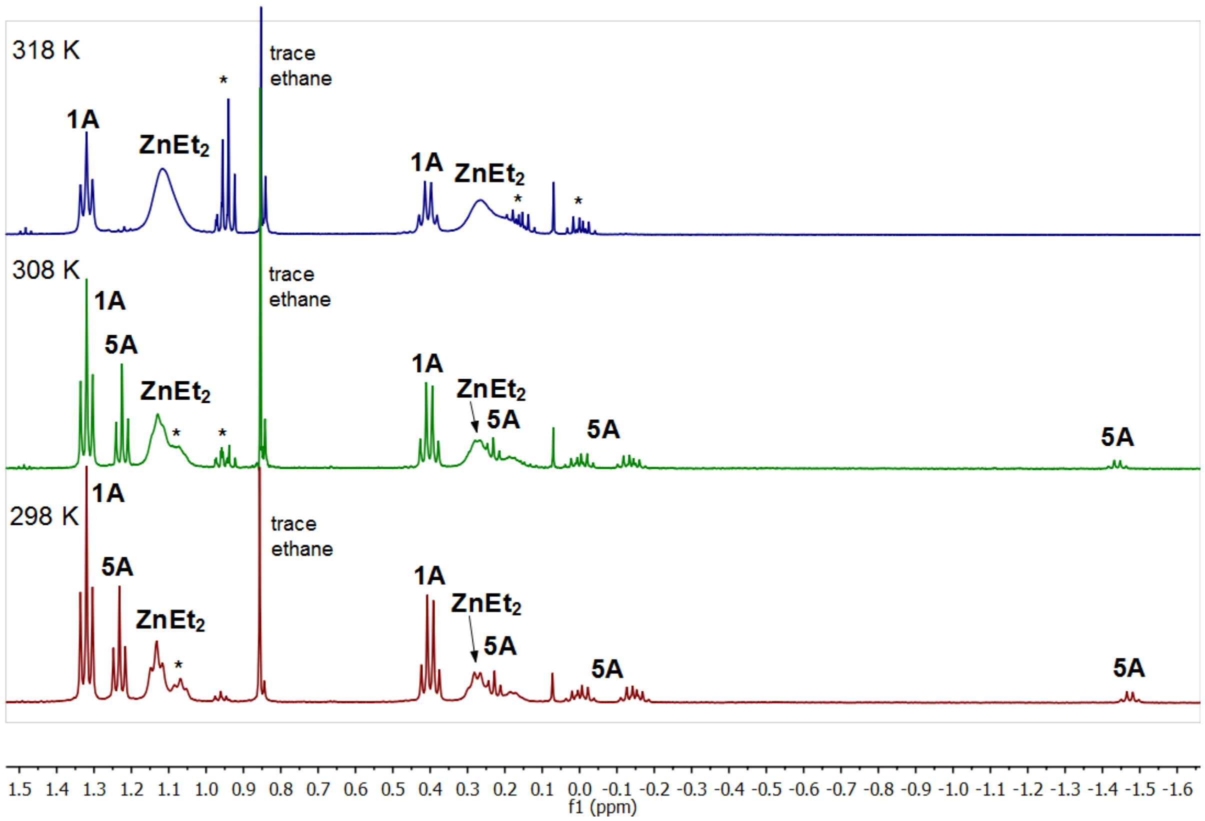

Supplementary Figure 43: ^{1}H NMR spectrum of 4 **2A** + 15 $ZnEt_2$ at three different temperatures. * marks unknown species which grow in over time (likely decomposition products).

<u>**Spectra of compounds 1C, 2C and 3C**</u>

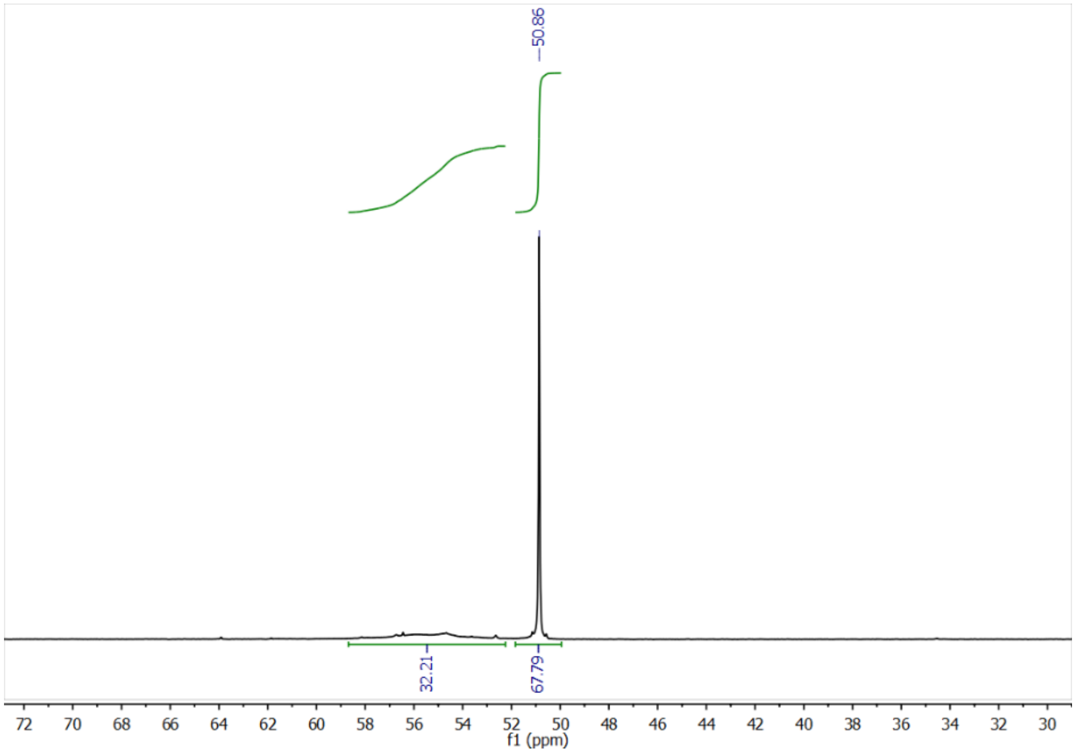

Supplementary Figure 44: $^{31}P\{^{1}H\}$ NMR spectrum of **1C**

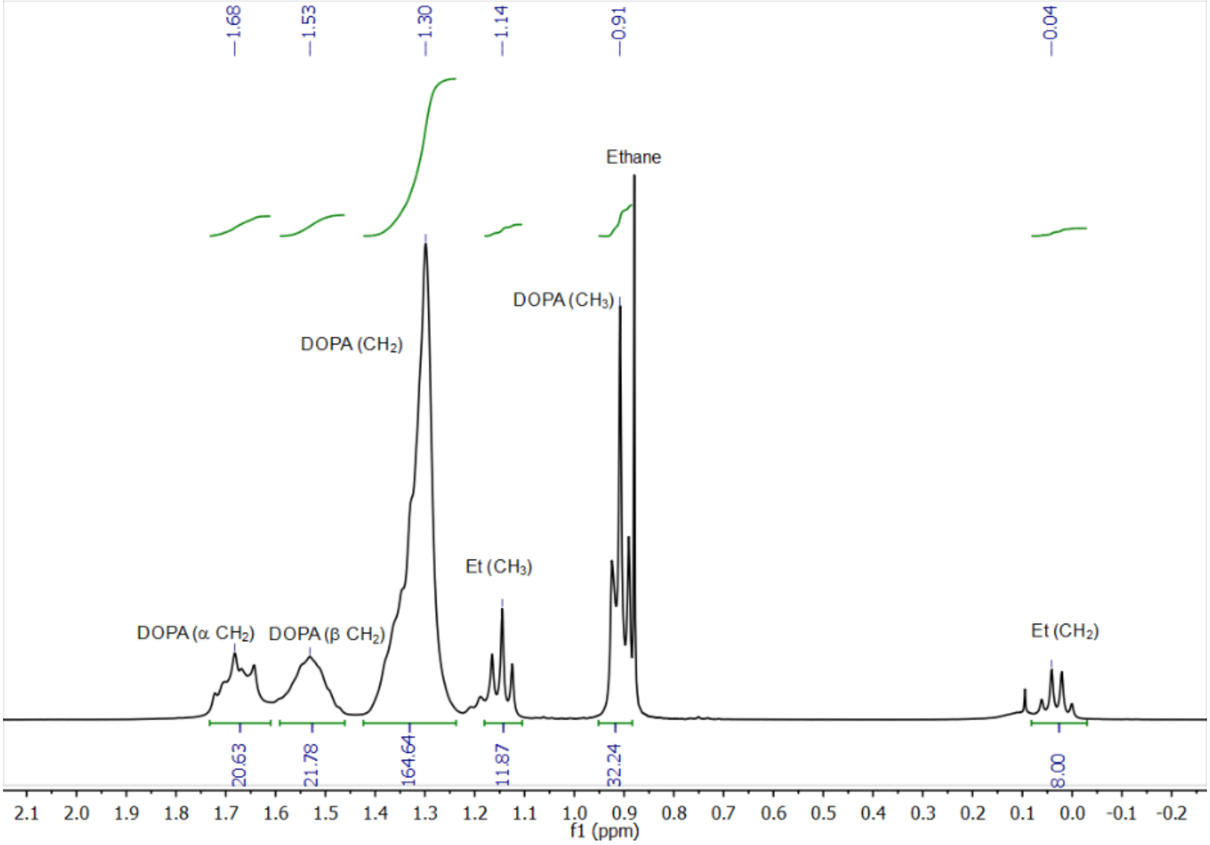

Supplementary Figure 45: ^{1}H NMR spectrum of **1C**

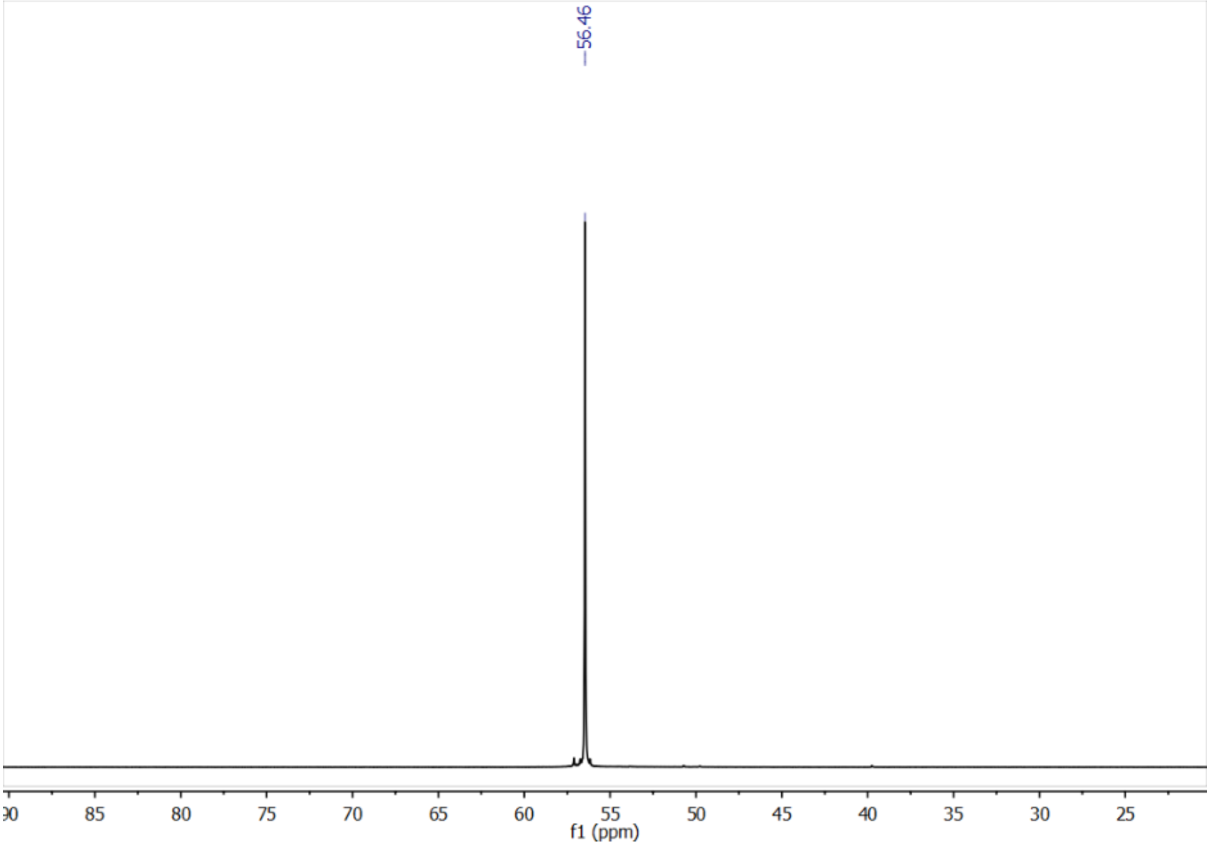

Supplementary Figure 46: ^{31}P{^{1}H} NMR spectrum of **2C**

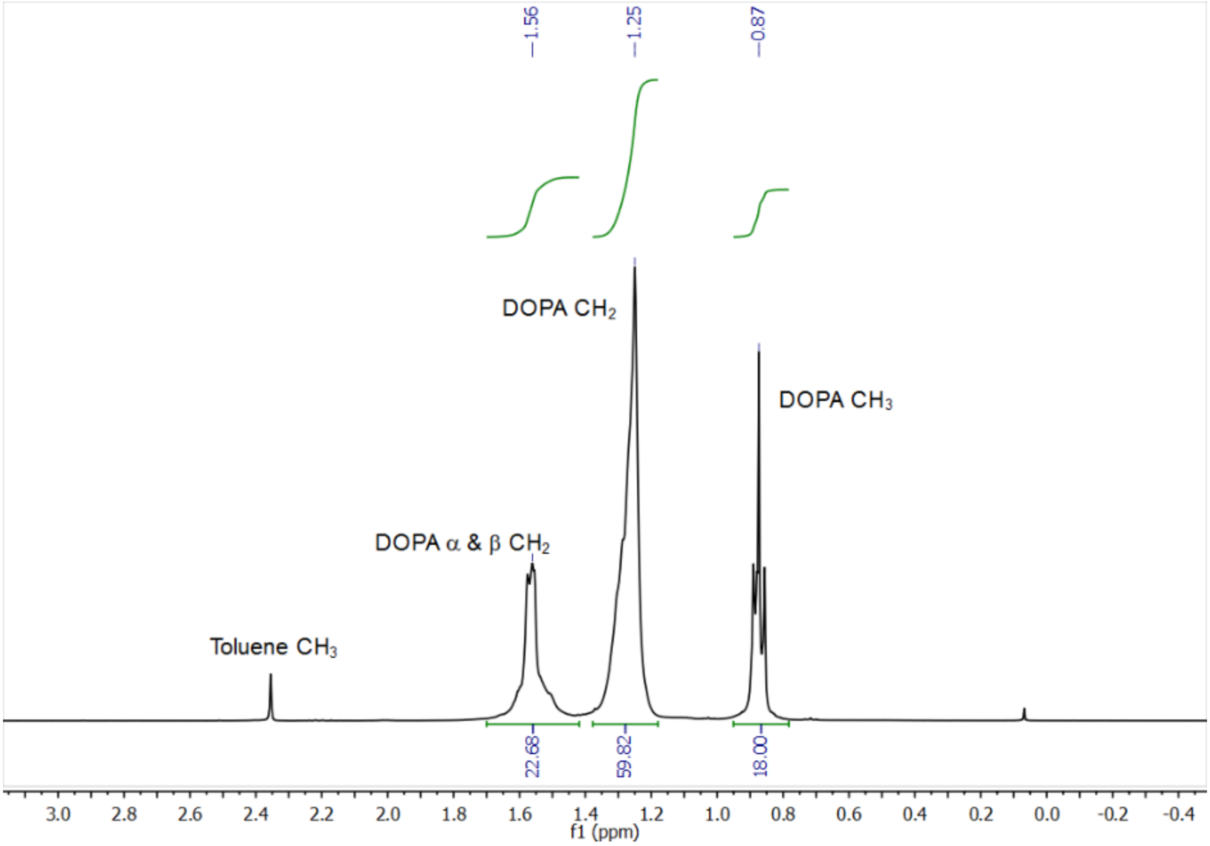

Supplementary Figure 47: ^{1}H NMR spectrum of **2C**

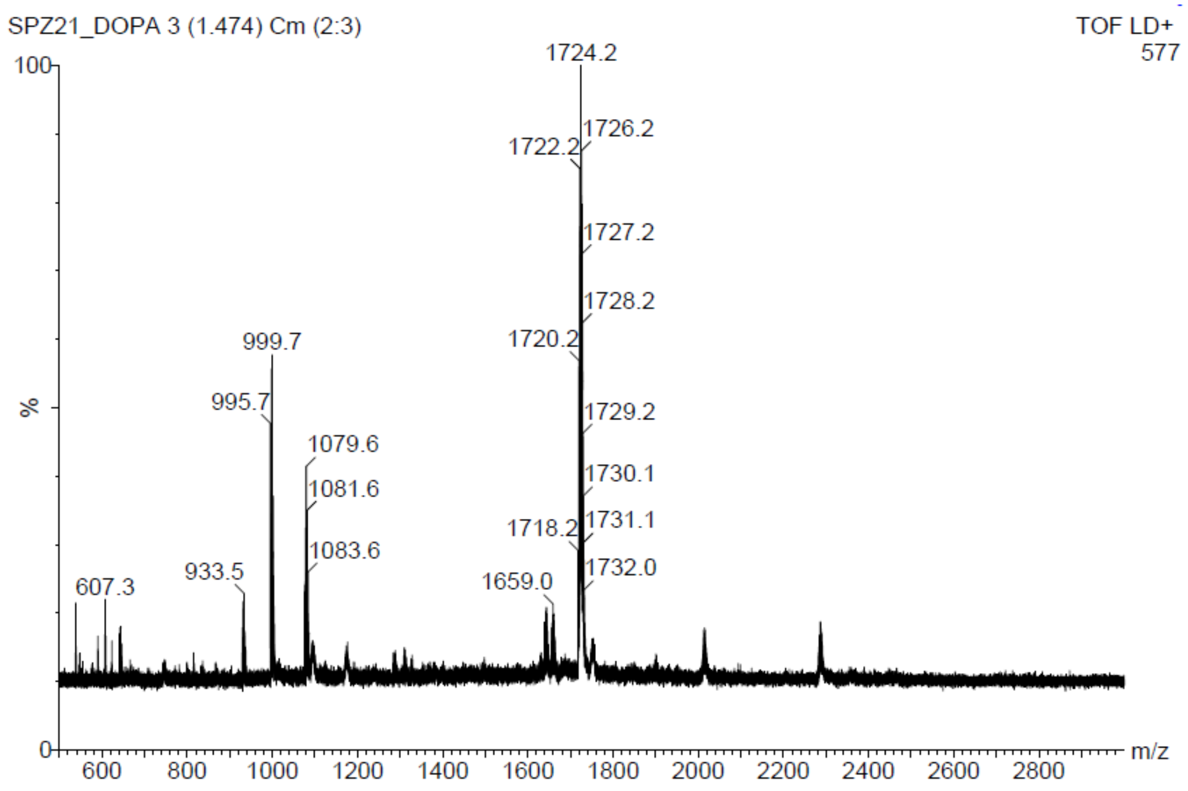

Supplementary Figure 48: MALDI mass spectrum of **2C**

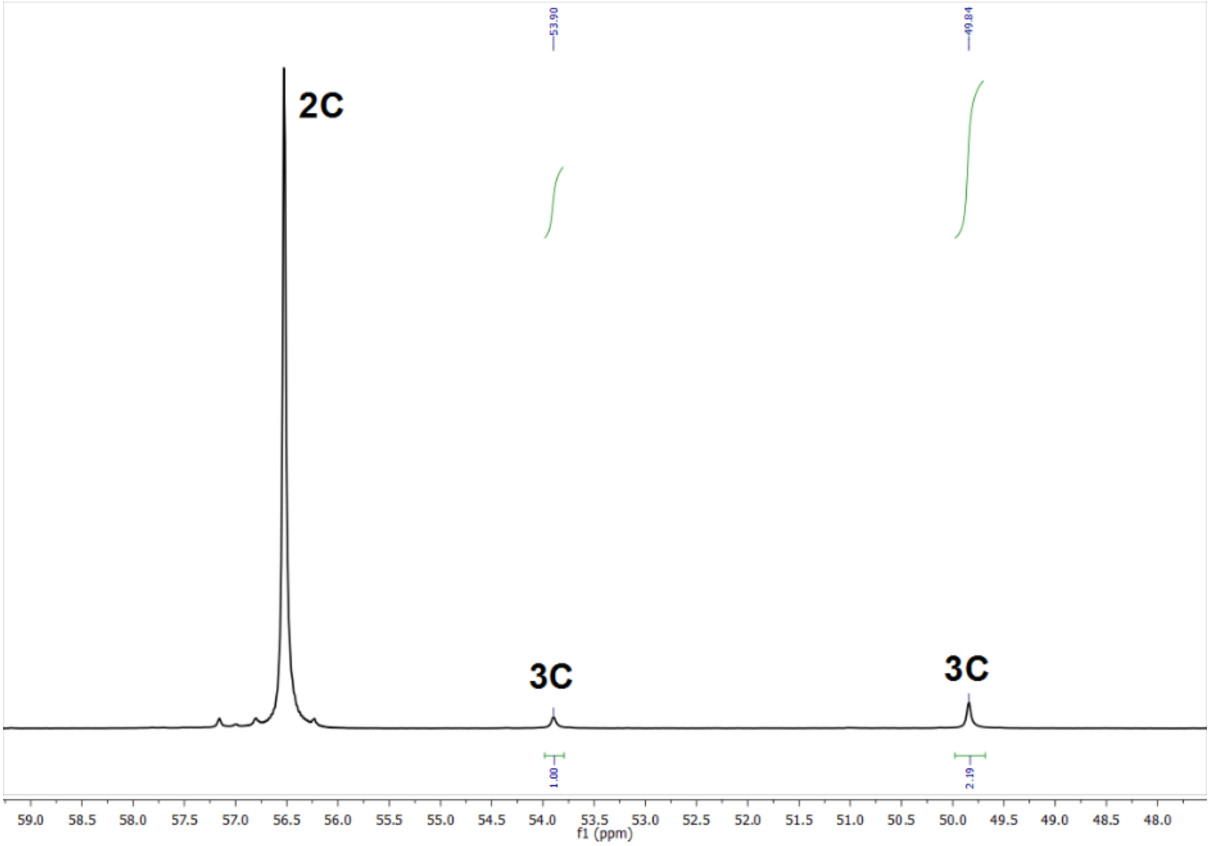

Supplementary Figure 49: $^{31}P\{^{1}H\}$ NMR spectrum of **2C** and water in equilibrium with **3C**

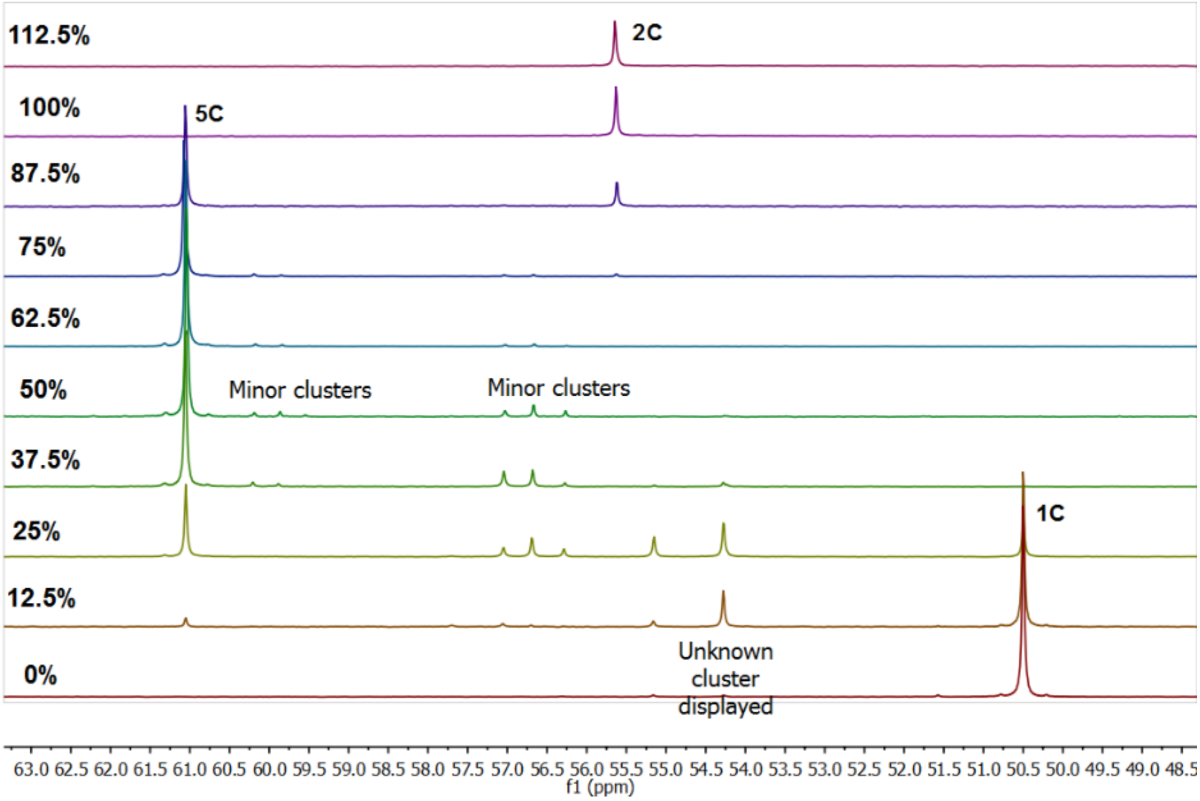

Supplementary Figure 50: $^{31}P\{^{1}H\}$ NMR spectra of a solution of a 5:1 ratio of $ZnEt_2$ and DOPA-H with varying amounts of water (100% = full hydrolysis of all Zn−Et bonds). "Unknown cluster" is displayed in Figure 6b of main text whilst other minor signals are included in Figure 6c of main text. The spectra reveal the initial presence of **1C** which is consumed into minor clusters and **5C** upon initial hydrolysis. **5C** grows to become the dominant species over the partial hydrolysis regime, before declining sharply upon complete hydrolysis. Traces of **2C** are clearly observed in the final spectra but a large broad signal for ligand coordinated to ZnO nanoparticles is also observed upon careful inspection.

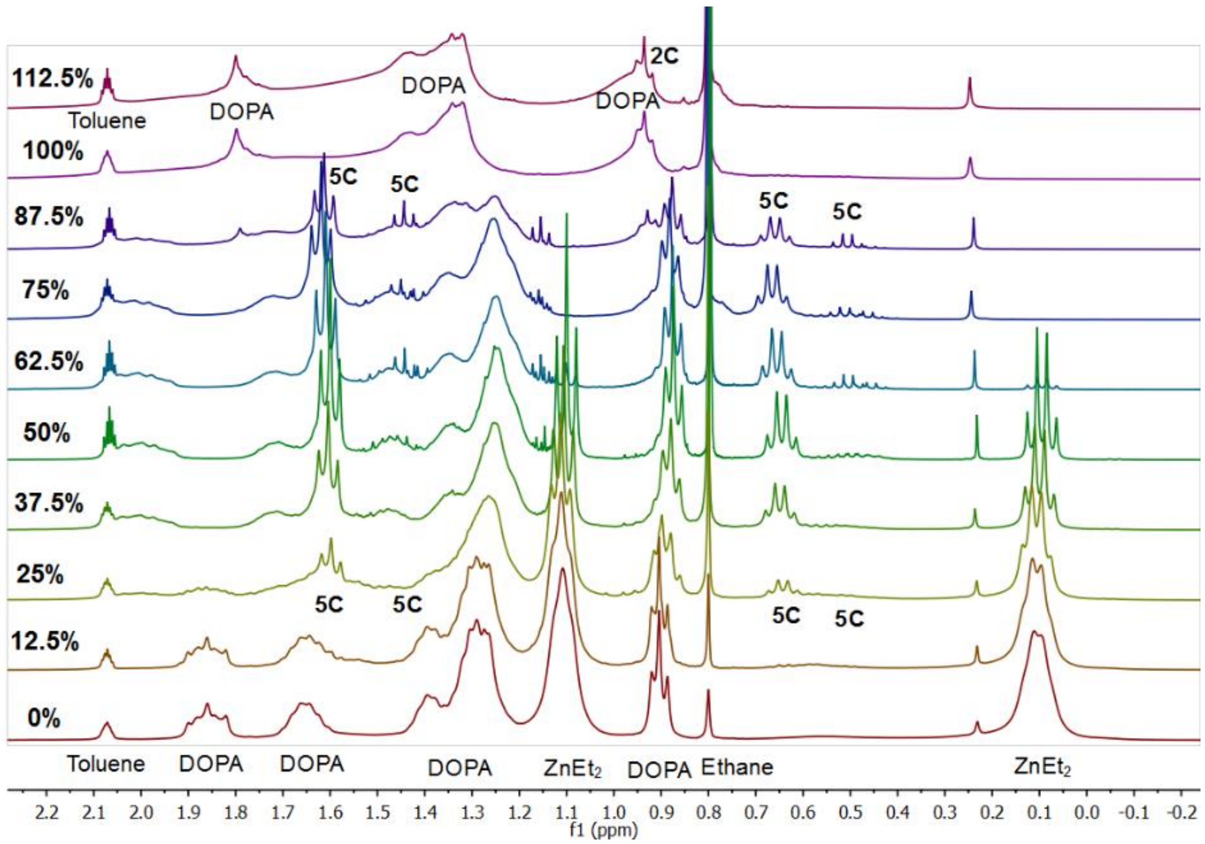

Supplementary Figure 51: ^{1}H NMR spectra of a solution of a 5:1 ratio of $ZnEt_2$ and DOPA-H with varying amounts of water (100% = full hydrolysis of all Zn−Et bonds). DOPA labels refer to the ligand in coordination to the various clusters/nanoparticles present at each stage (e.g. initially as **1C** then becoming mainly **5C** and finally **2C**/ZnO@DOPA). The spectra show the gradual consumption of $ZnEt_2$ signals, which begin as broad signals in equilibrium with **1C** but sharpen as **1C** is consumed. As **1C** is hydrolysed new ethyl signals assigned to **5C** grow in (δ 0.66, 1.63) as confirmed by independent synthesis of **5C** in d_8-toluene [Supplementary Figure 56:]. A second ethyl signal appears at about 50% hydrolysis (δ 0.50, 1.44) this signal sharpens to ¼ of the integral of the major **5C** ethyl signal once free $ZnEt_2$ is consumed – this could possibly be the second ethyl environment of **5C** which may undergo ethyl exchange in the presence of free $ZnEt_2$. No clear species evolve in the ^{31}P spectrum in concert with the signals at (δ 0.50, 1.44) and these signals are lost upon the consumption of the other signals for **5C**. Upon full hydrolysis a shift is seen for the DOPA resonances as the ligand switches from well-defined molecular clusters to the surface of ZnO nanoparticles, smaller sharp resonances are found for traces of **2C** which also forms.

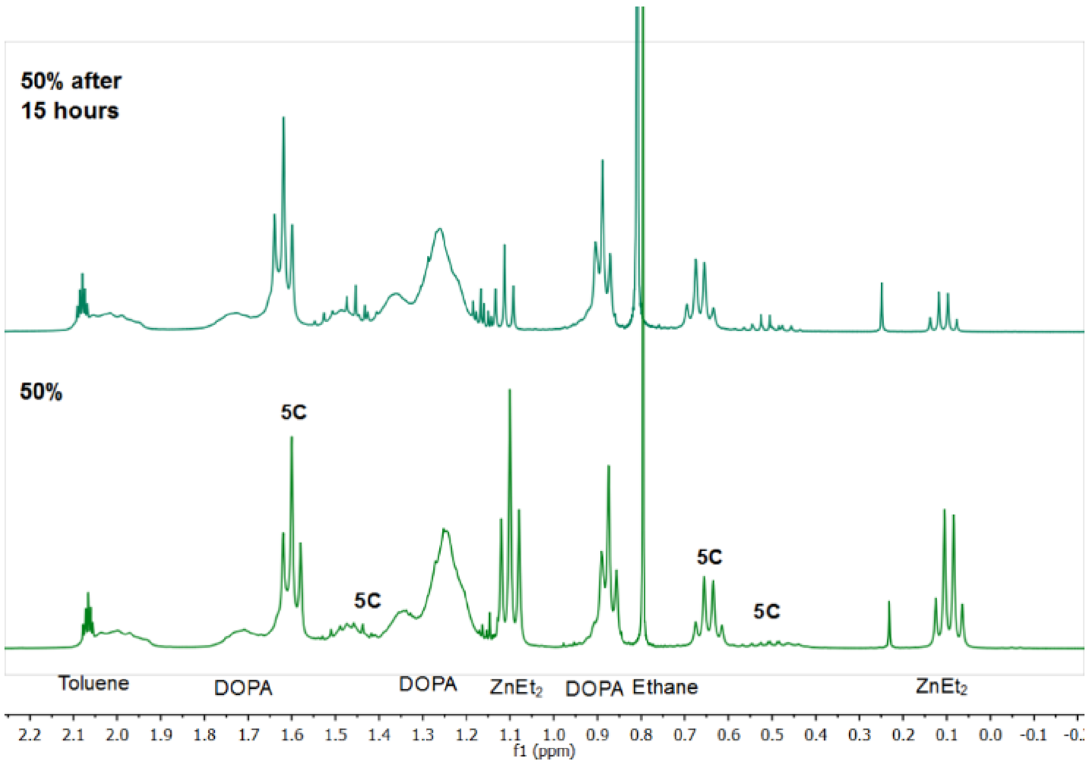

Supplementary Figure 52: ^{1}H NMR spectra of a solution of a 5:1 ratio of $ZnEt_2$ and DOPA-H with 2 µl added water giving 50% = hydrolysis of all Zn−Et bonds, before and after 15 hour waiting period. Although little change occurs to the DOPA containing species some further consumption of free $ZnEt_2$ does occur – this suggests that a small amount of moisture had not fully incorporated into the toluene solution during the initial 30 minute mixing period. Some minor species are lost over this waiting period, however, interestingly they re-appear upon addition of the next aliquot of water.

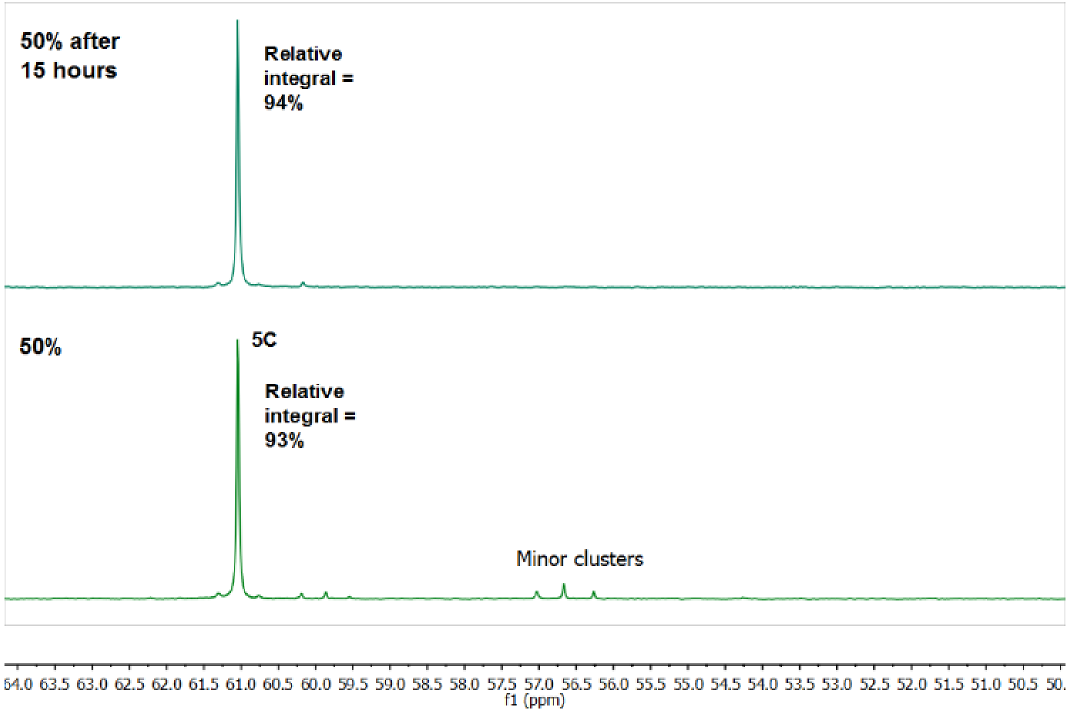

Supplementary Figure 53: $^{31}P\{^{1}H\}$ NMR spectra of a solution of a 5:1 ratio of $ZnEt_2$ and DOPA-H with 2 µl added water giving 50% = hydrolysis of all Zn−Et bonds, before and after 15 hour waiting period.

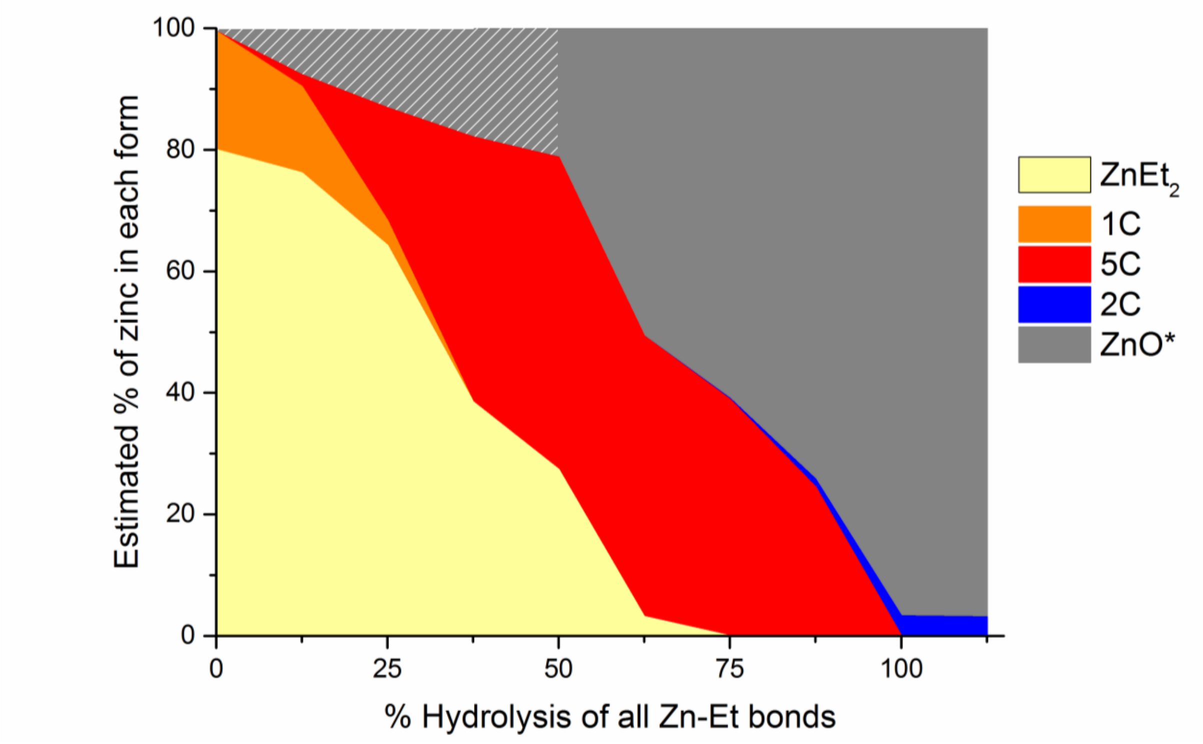

Supplementary Figure 54: Area graph showing estimated % of total zinc in various forms in a solution of a 5:1 ratio of $ZnEt_2$ and DOPA-H with varying amounts of added water (100% = hydrolysis of all Zn−Et bonds). It is assumed that the initial reaction (before the addition of water) absorbs 20% of the total zinc into cluster **1C** leaving 80% as free $ZnEt_2$. The $ZnEt_2$ fraction is estimated from ^{1}H integrals of $ZnEt_2$ from NMR spectra relative to the internal standard (PPh_3 phenyl groups). **1C** (4x Zn), **5C** (11x Zn) and **2C** (4x Zn) fractions are estimated from relative ^{31}P integrals. *ZnO fraction is assumed to make up the zinc balance to 100% - this does not take into account other unknown minor species and so will be a slight overestimate especially at low levels of hydrolysis indicated by dashed region.

NMR spectra of 5C

Isolating 5C as an oil led to partial decomposition/reaction with trace impurities and so the most representative spectra were taken directly from the reaction solution (protonated solvent). The position of the ethyl signals varies with solvent choice hence a $CDCl_3$ and H^8-toluene ^1H NMR spectrum are included here. The CH_3 signal of the minor Et component of **5C** is not located in either solvent and is likely obscured by other signals.

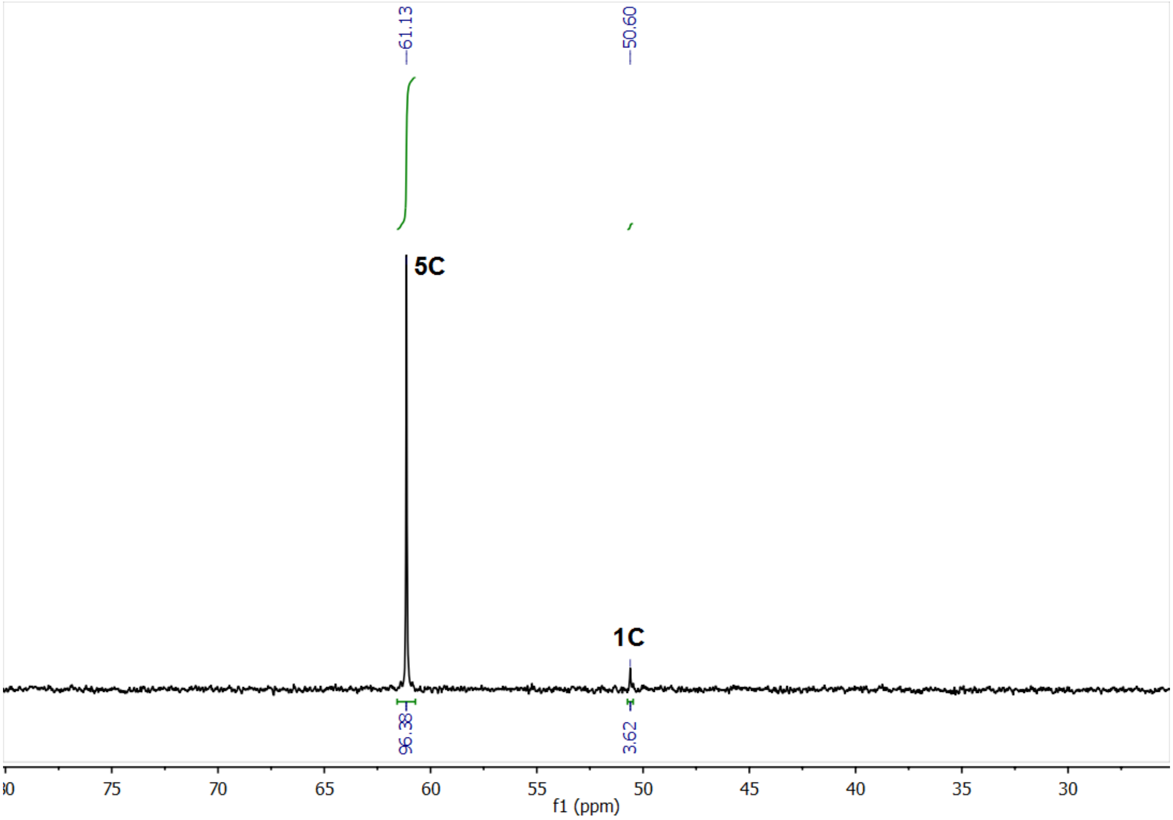

Supplementary Figure 55: $^{31}P\{^1H\}$ NMR spectrum of **5C** taken directly from reaction solution (H^8-toluene). Trace impurity of known species **1C**.

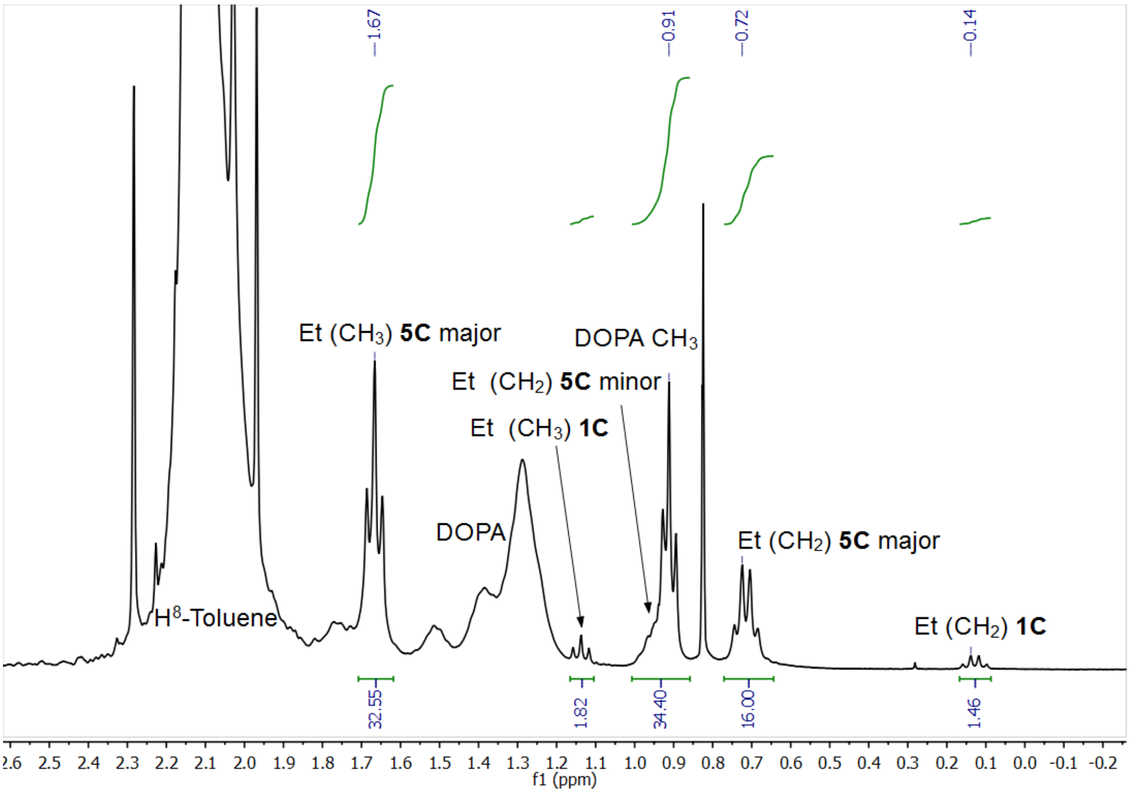

Supplementary Figure 56: ^{1}H NMR spectrum of **5C** taken directly from reaction solution (H^{8}-toluene). Trace impurity of known species **1C**. Et (CH_3) signal for the minor ethyl signal of **5C** not located.

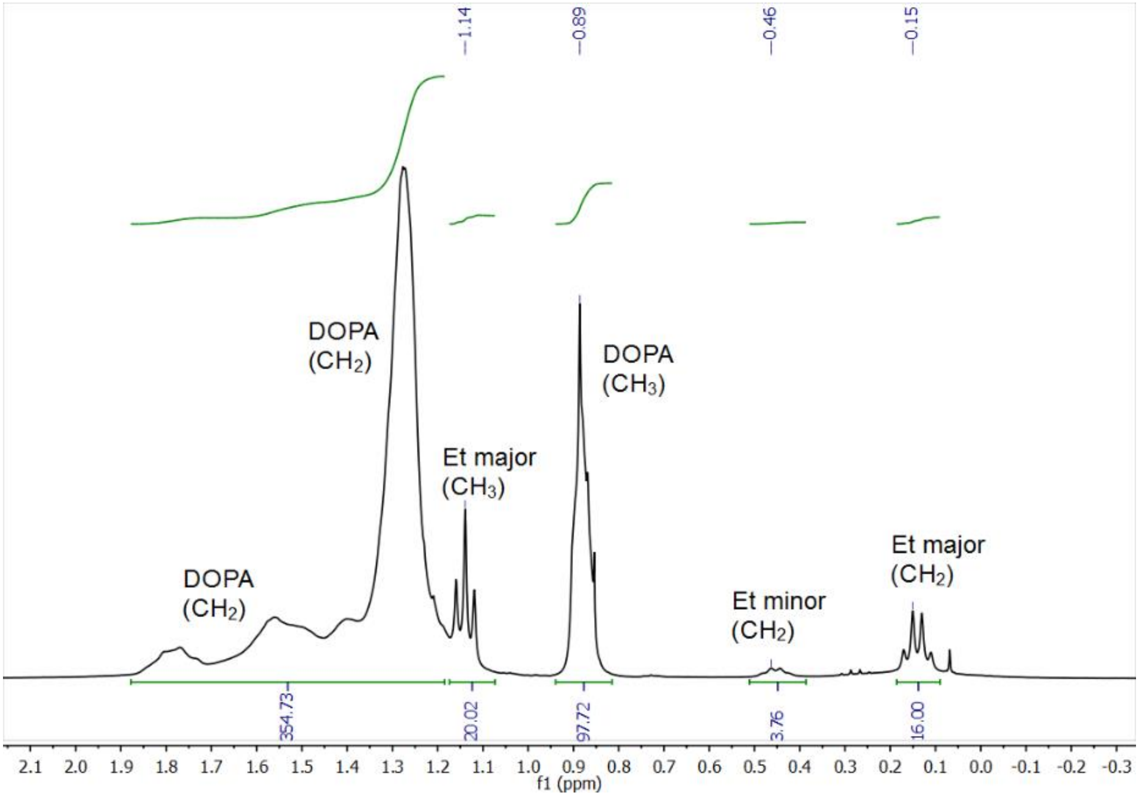

Supplementary Figure 57: ^{1}H NMR spectrum of **5C** in $CDCl_3$ (with minor impurities, consistent spectra were achieved on repeated occasions).

Reaction of $ZnEt_2$ with 2C

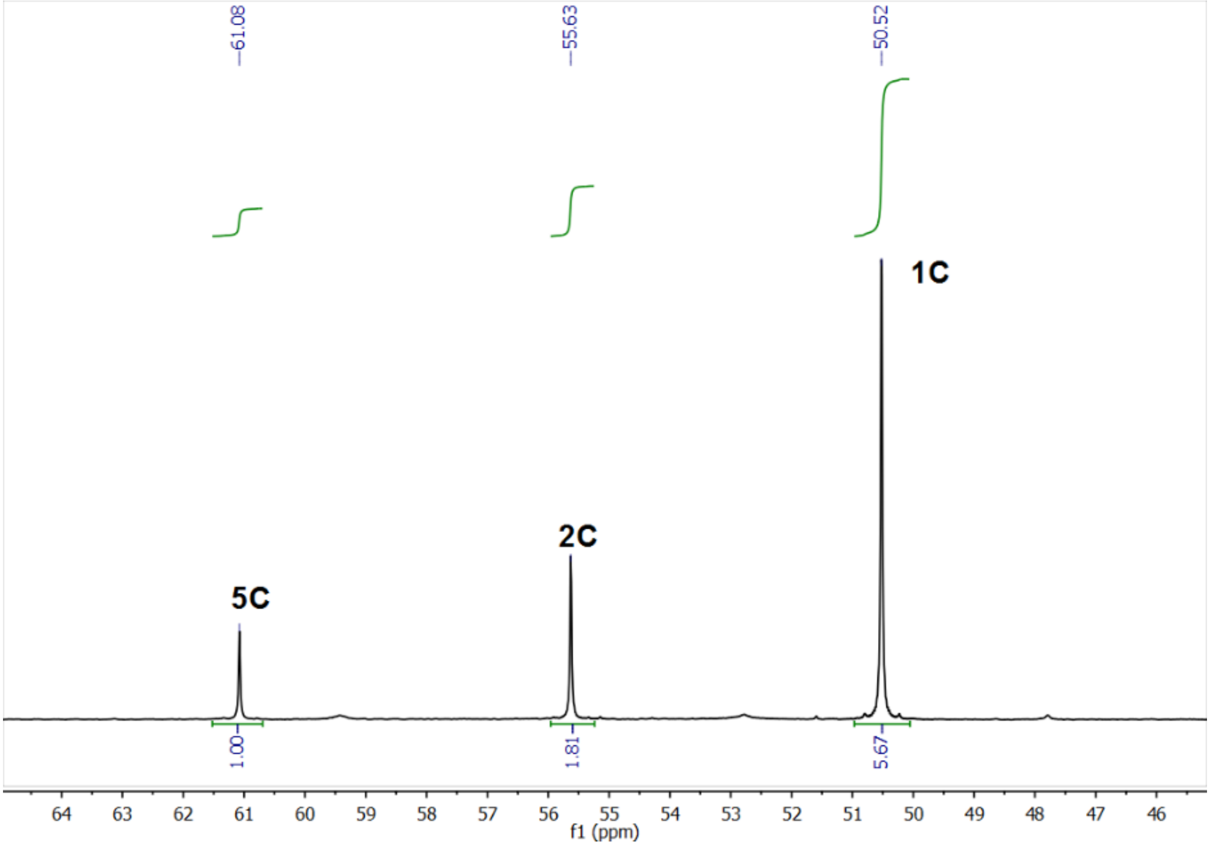

Supplementary Figure 58: $^{31}P\{^{1}H\}$ NMR spectra after the addition of 4 equivalents of $ZnEt_2$ to a solution of **2C**. It is known that addition of $ZnEt_2$ to solutions of **2A/B** forms an equilibrium generating a 5:1 ratio of **1A/B**:**5A/B**. To test that the same process occurs with **2C** four equivalents of $ZnEt_2$ were added to a solution of **2C**. The resulting spectrum shows the formation of a 5:1 ratio of signals for **1C** and **5C** as expected with residual **2C** also observed.

Characterisation of 'Yellow ZnO'

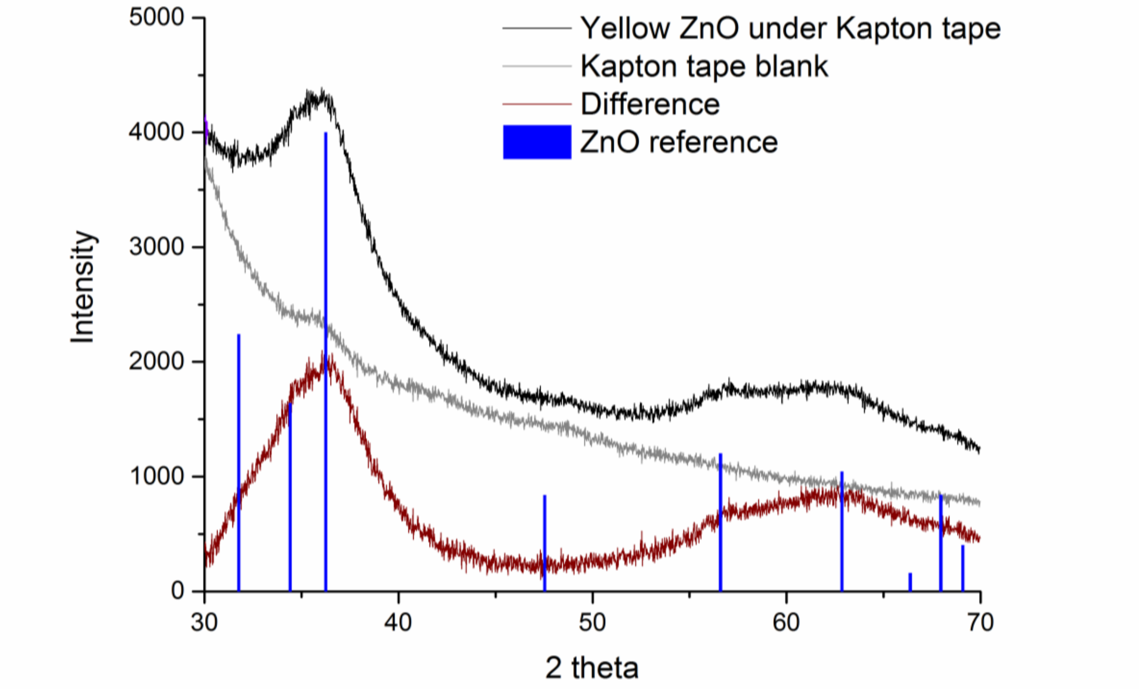

Supplementary Figure 59: Powder X-ray diffraction of isolated 'yellow ZnO' powder, prepared in a glove-box and sealed using Kapton tape to ensure an air free environment. Strong signals for Kapton tape are observed between 2θ =10-30° but unlike the signal at 2θ = 36° these signals are lost once the tape is removed. Wurzite ZnO pattern indicated below (JCPDS card 00-001-1136).[1] The pattern was too broad for sensible Scherrer analysis, however in comparison with known XRD spectra of ZnO nanoparticles (see Supplementary Figure 69:) we would anticipate any ZnO crystallites to be smaller than 2 nm in diameter.

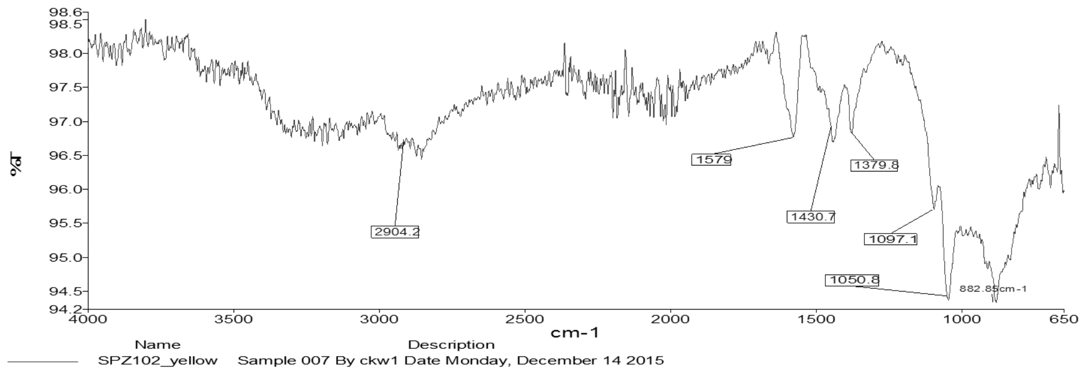

Supplementary Figure 60: ATR-IR spectra of 'yellow ZnO'. Under air. Evidence of weak C-H stretches attributed to residual ethyl groups, otherwise very weak absorbances.

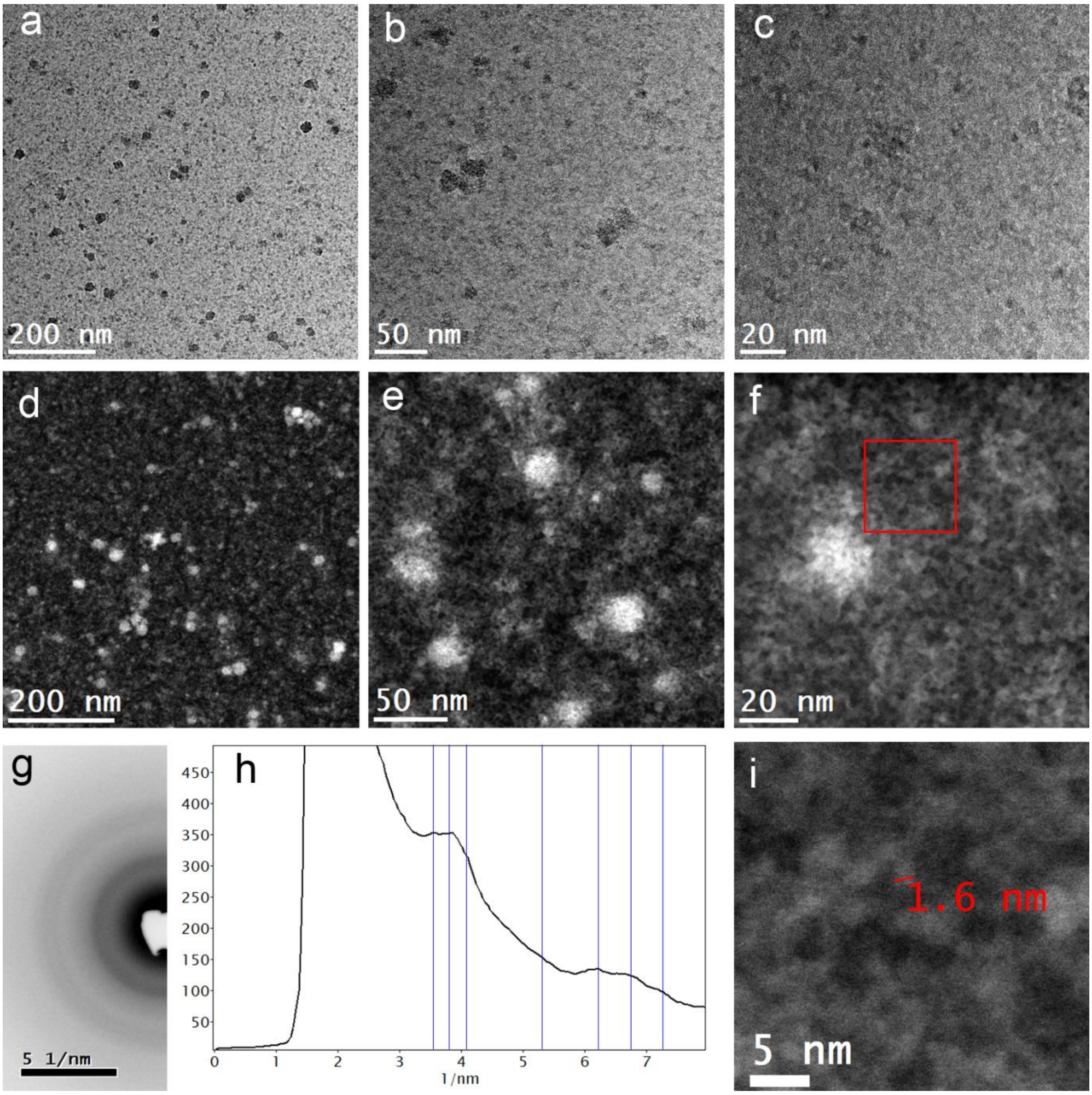

Supplementary Figure 61: Conventional TEM (a-c) and STEM images (annular dark field mode) (d-f) of the 'yellow ZnO'. The yellow ZnO uniformly coats the TEM grid apart from some agglomerates ~20 nm in diameter. (g) Electron diffraction pattern of the region shown in **a**. (h) Rotational average of **g**. While the yellow ZnO appears amorphous in the images, the electron diffraction pattern shows several broad rings. The blue lines overlaid in **h** indicate expected peak locations for wurtzite ZnO, which overlap with the peaks in the rotational average. (i) Higher magnification STEM image of the region outlined in the red box of **f**. One of the smallest resolvable features, possibly a small ZnO crystallite, is sub 2 nm in size.

UV spectroscopy study of synthesis of ZnO nanoparticles by hydrolysis route

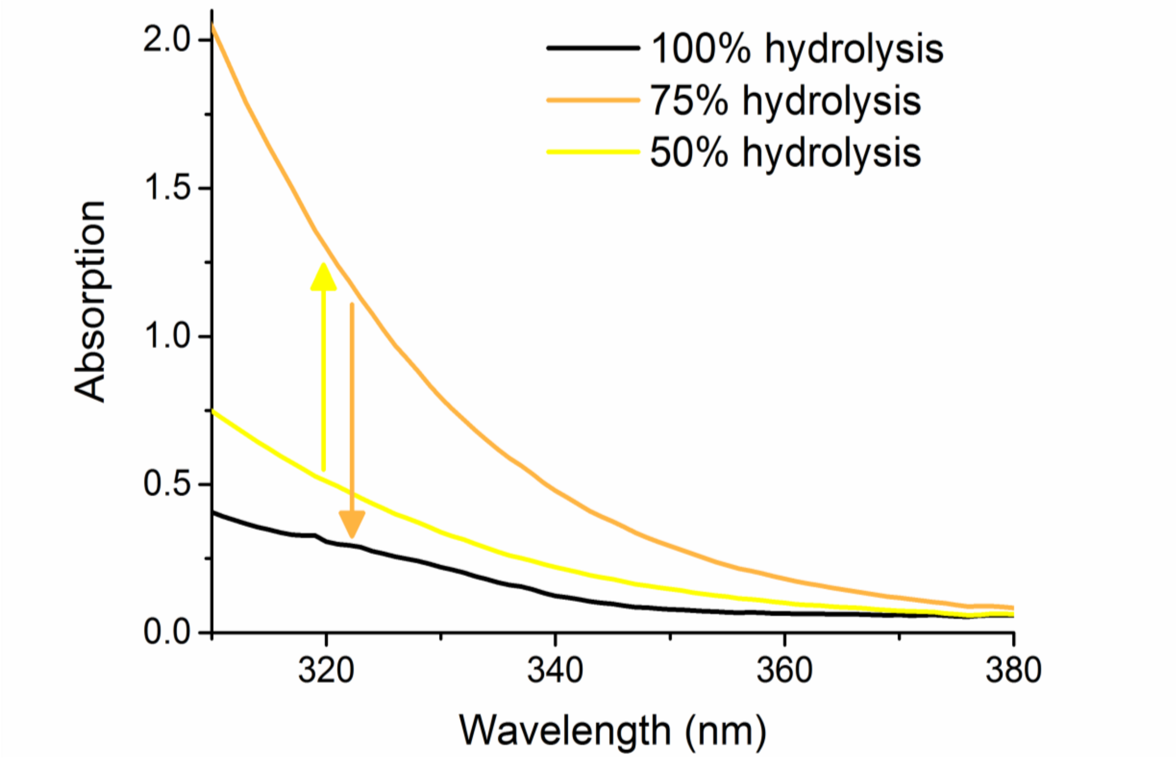

Supplementary Figure 62: UV spectra of partially and fully hydrolysed samples, [Zn] = $4x10^{-3}$ M, at 100% hydrolysis a band edge is observed corresponding to ZnO nanoparticles of ~3 nm diameter (calculated from a more concentrated spectrum, Supplementary Figure 63:). After initial UV spectra an excess of water was added to the 75% hydrolysed sample and a further spectrum collected, this confirmed that the intensity of UV absorption is reduced and a characteristic ZnO band-edge forms upon full hydrolysis. The fully hydrolysed sample displayed a characteristic ZnO band edge in its UV spectrum, from this the size of the resultant ZnO nanoparticles could be estimated by the Meulenkamp method.[2]

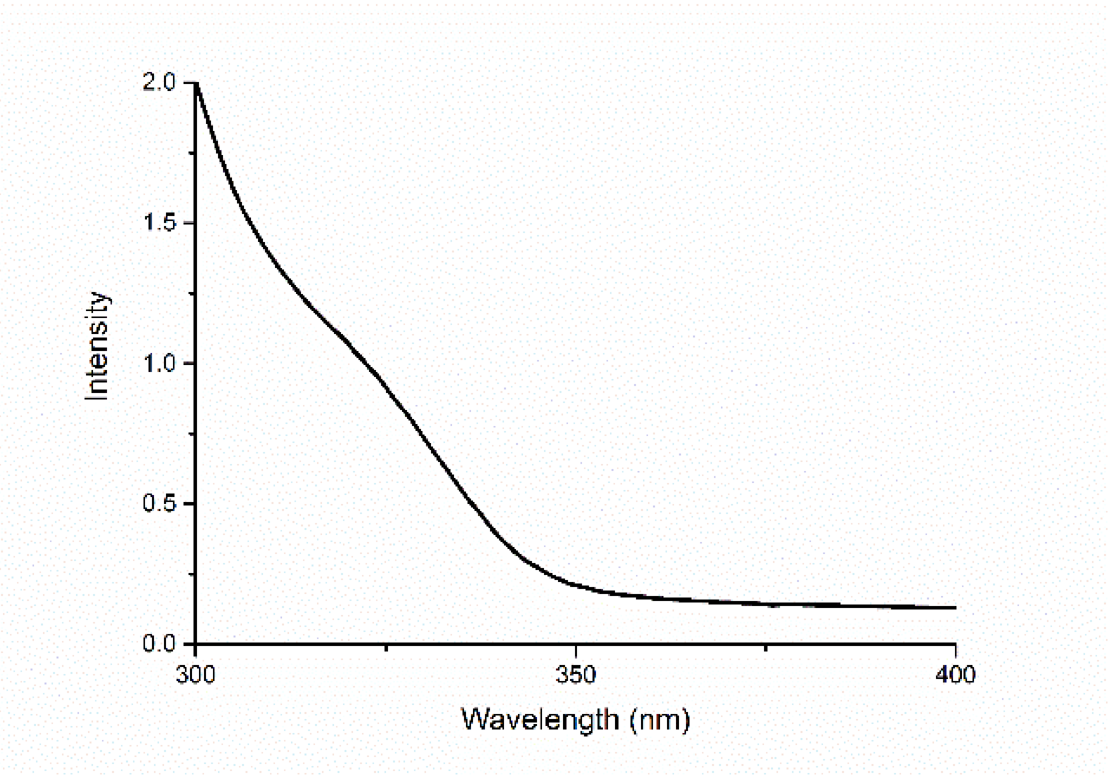

Supplementary Figure 63: UV spectrum of ZnO@DOPA nanoparticles generated during UV spectroscopy investigations after complete hydrolysis. Estimated size = 2.8 nm diameter particles

Characterisation of ZnO@DOPA nanoparticles (after complete hydrolysis)

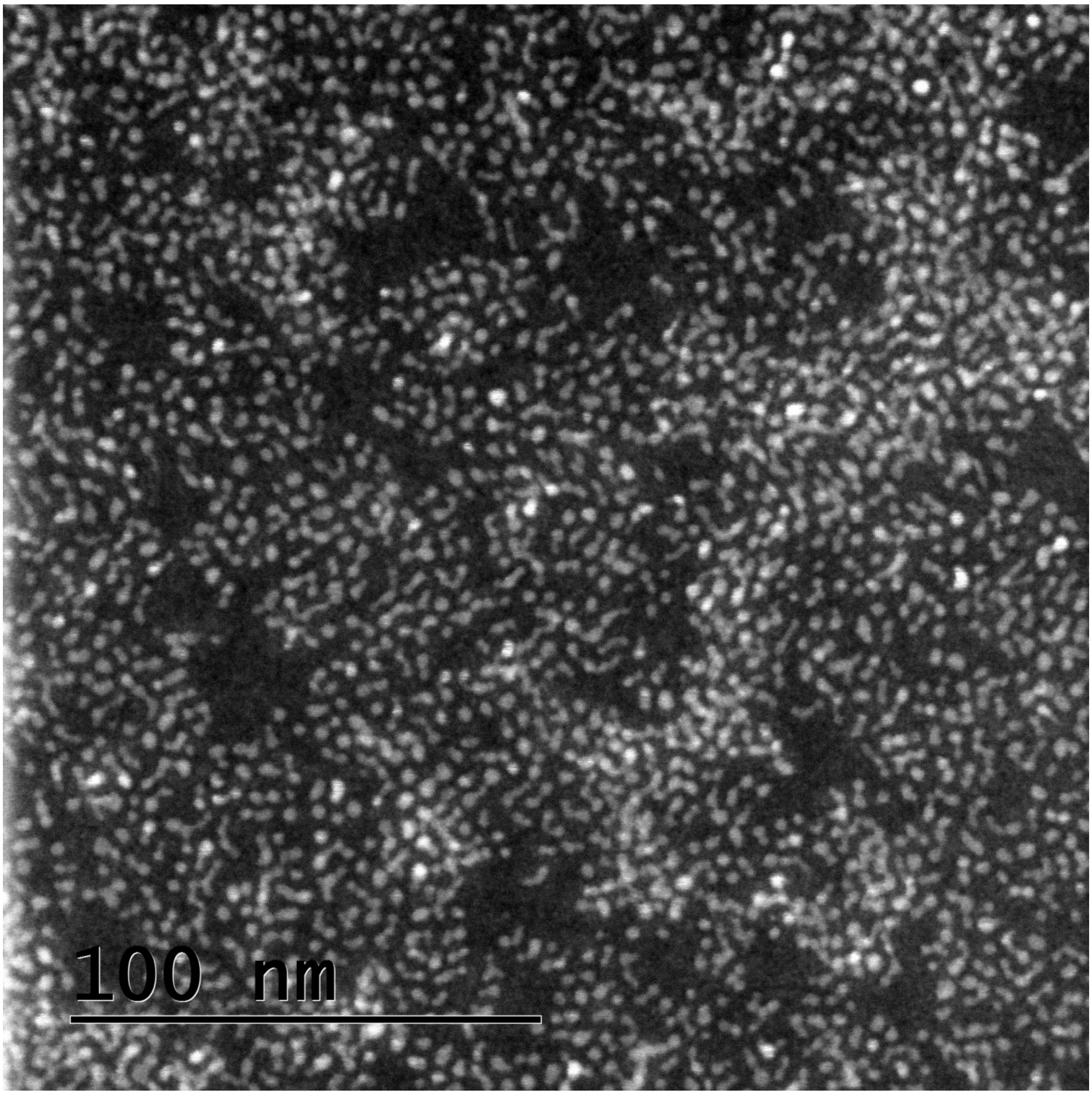

Supplementary Figure 64: Representative image of ZnO@DOPA nanoparticles acquired using scanning transmission electron microscopy in annular dark field mode.

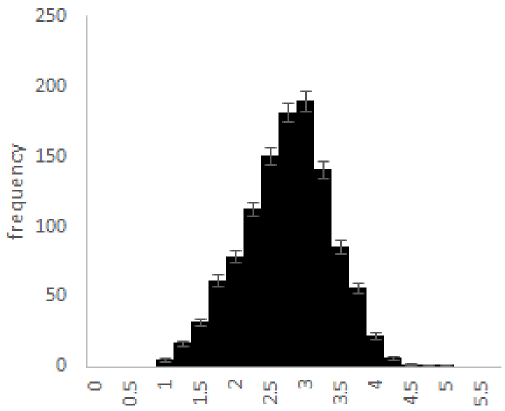

Supplementary Figure 65: Histogram displaying measured sizes of ZnO@DOPA nanoparticles from STEM images. Average size = 2.75 ± 0.02 nm (S.D. = 0.6 nm)

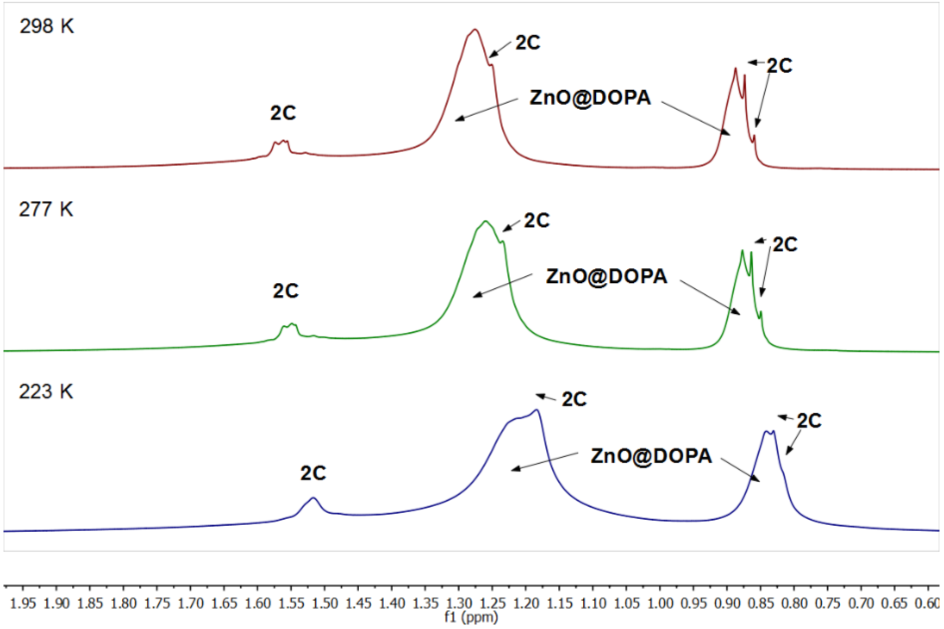

Supplementary Figure 66: ^{1}H NMR spectrum of ZnO@DOPA nanoparticles at three different temperatures. A small trace of **2C** (see Supplementary Figure 47:) is observed as a sharp overlapping signal. Broad signals are observed for the ZnO@DOPA nanoparticles whilst sharp signals are observed for a trace of by-product **2C**. No apparent change occurs upon cooling the solution.

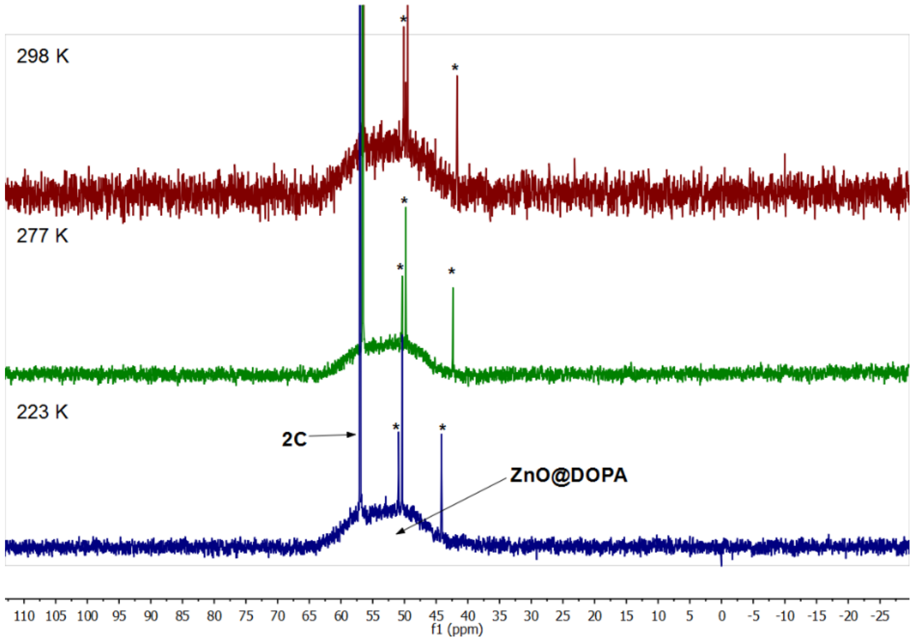

Supplementary Figure 67: ^{31}P{^{1}H} NMR spectrum of ZnO@DOPA nanoparticles at three different temperatures. A small trace of **2C** is observed as a sharp signal and trace unknown signals are observed (marked with *, please note these spectra are magnified to show the broad signal and the impurity species, which may be $Zn(DPPA)_2$ or similar, are very minor signals). No apparent change occurs upon cooling the solution.

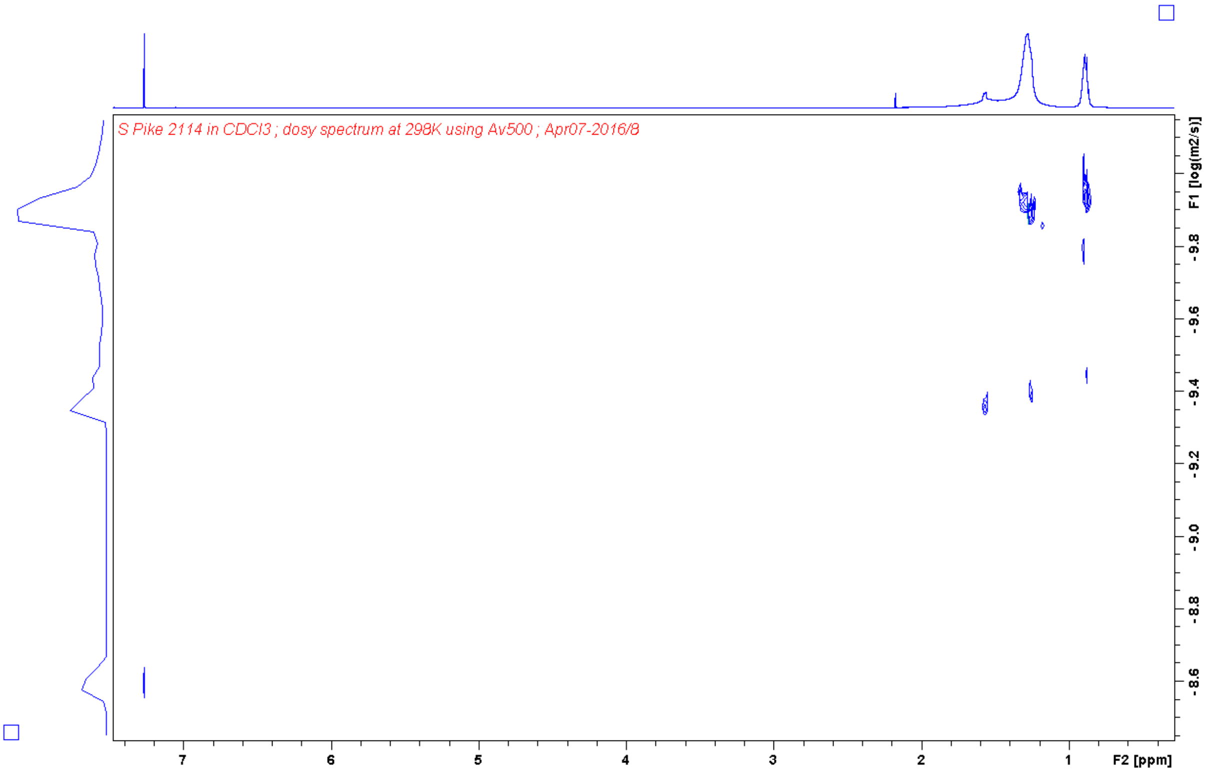

Supplementary Figure 68: ^{1}H DOSY NMR spectrum of ZnO@DOPA nanoparticles with a small trace of **2C** present. Diffusion coefficients are calculated as: ZnO@DOPA, 1.25×10^{-10} m^2/s; **2C**, 4.35×10^{-10} m^2/s; $CDCl_3$, 2.59×10^{-9} m^2/s. Using the Stokes-Einstein equation the diameter of the various fractions is estimated as (assuming a spherical shape): ZnO@DOPA, 6.2 ±1.1 nm; **2C**, 1.9 ±0.2 nm; $CDCl_3$, 0.3 ±0.1 nm. The sizes for the ZnO crystalline cores for this sample are estimated to be 2-2.9 nm by X-ray diffraction and UV spectroscopy techniques. Since the DOPA ligand is expected to be around 1 nm in length, a full diameter of around 4-5 nm is anticipated, in fairly good agreement with the DOSY analysis. **2C** has an estimated diameter of 2.9 nm if the DOPA ligands are fully extended. It is important to note that the ligands attached to ZnO nanoparticles or the cluster **2C** show no evidence of exchange by this technique (in which case diffusion coefficients between the two size regimes might be expected).[3]

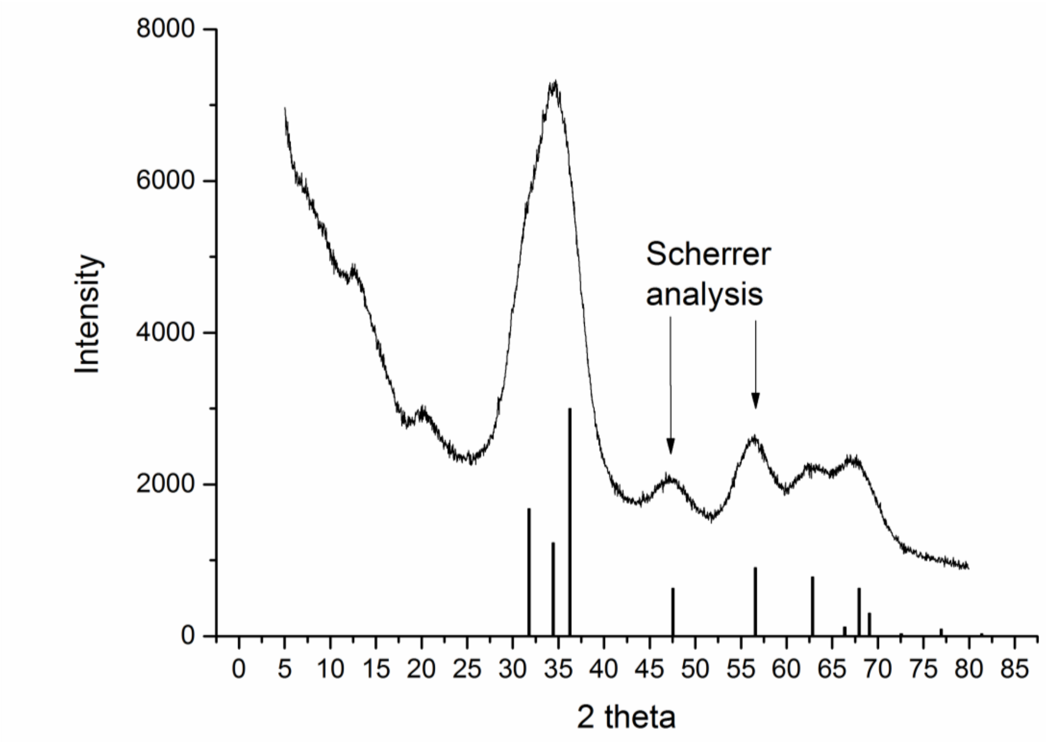

Supplementary Figure 69: Powder XRD spectrum of a typical batch of ZnO nanoparticles prepared from a 5:1 ratio of $ZnEt_2$ and DOPA-H. Analysis using the Scherrer equation suggests a consistent diameter in two perpendicular directions of between 1.9-2.5 nm [2θ = 47.43, hkl = 102, d = 2.3-2.5 nm; 2θ = 56.55, hkl = 110, d = 1.9-2.0 nm], size analysis by UV spectroscopy suggested 2.7 nm diameter in this case. N.B. some ripening does occur over time once the particles are isolated.[4] Wurzite ZnO reference pattern indicated (JCPDS card 00-001-1136).[1]

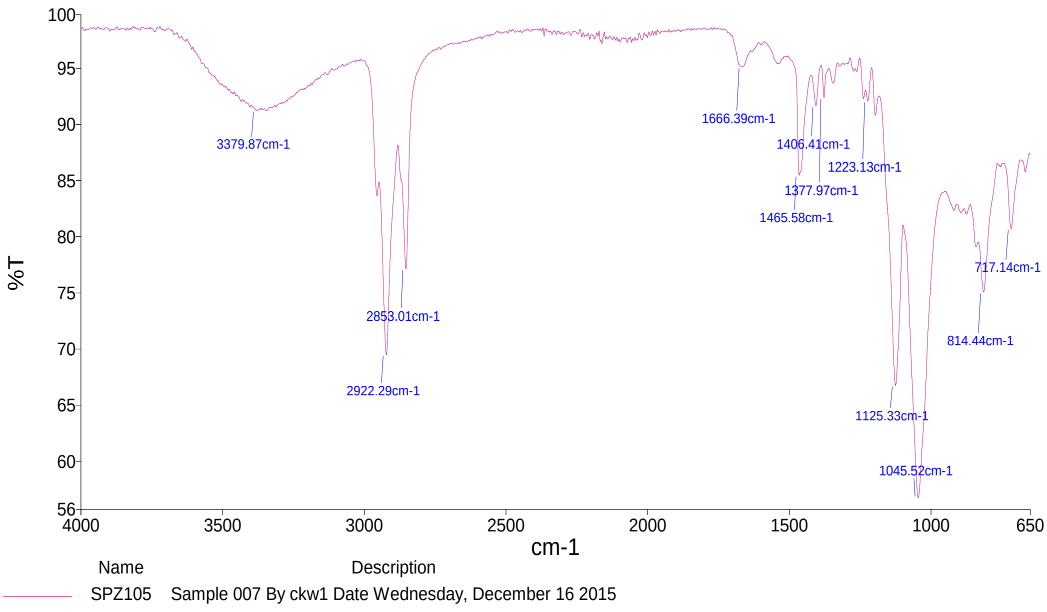

Supplementary Figure 70: FTIR spectrum of a typical batch of ZnO@DOPA nanoparticles prepared from a 5:1 ratio of $ZnEt_2$ and DOPA-H

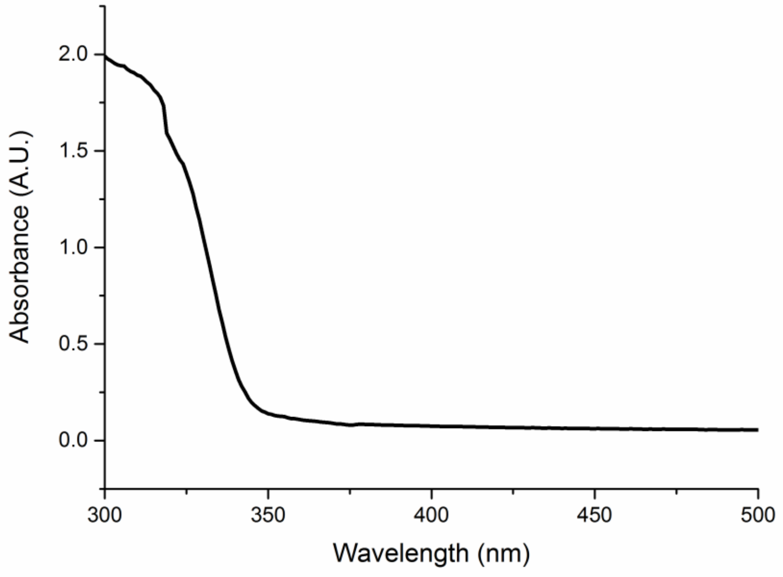

Supplementary Figure 71: UV/vis spectrum of a typical batch of ZnO@DOPA nanoparticles prepared from a 5:1 ratio of $ZnEt_2$ and DOPA-H in toluene

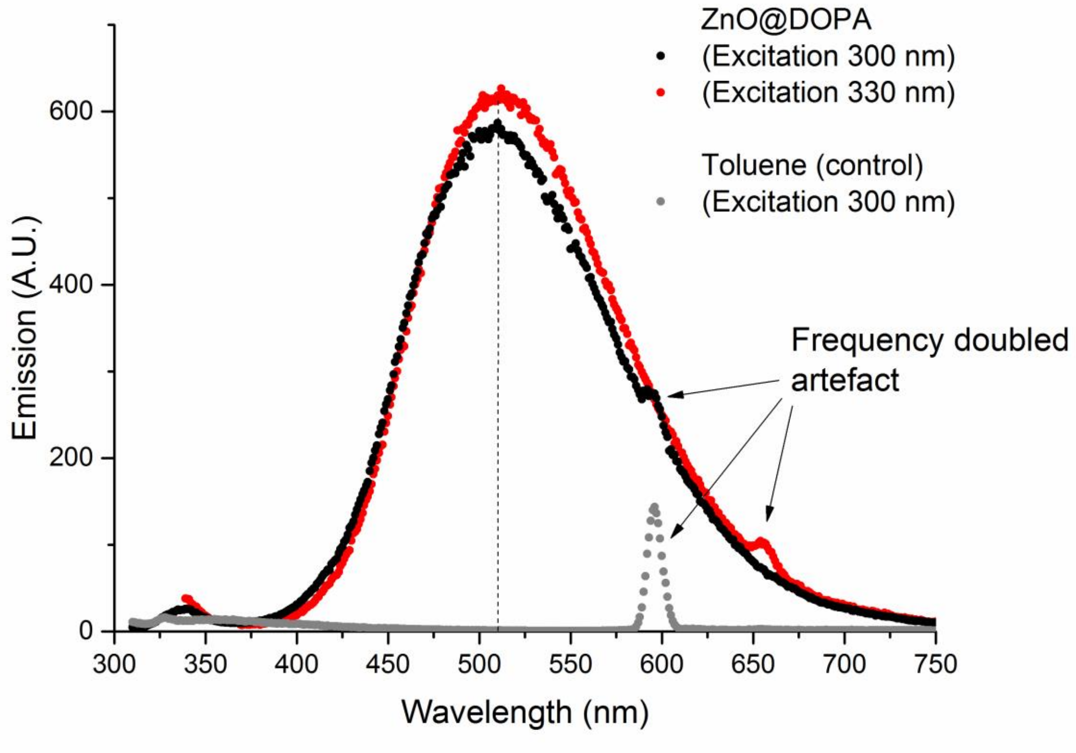

Supplementary Figure 72: Photoluminescence spectrum (excitation at 300 or 330 nm, in toluene, blank solvent spectrum included) of a typical batch of ZnO@DOPA nanoparticles prepared from a 5:1 ratio of $ZnEt_2$ and DOPA-H. Emission maximum located at ~510 nm.

Characterisation of ZnO nanoparticles without capping ligand

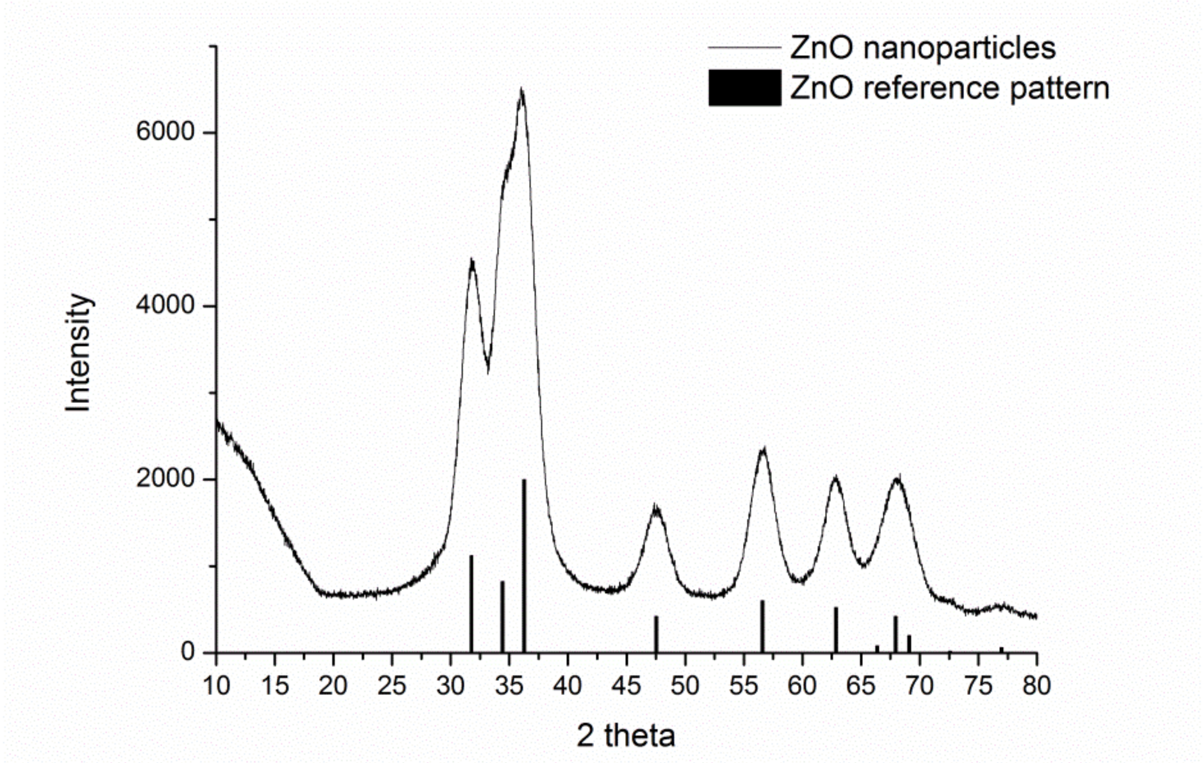

Supplementary Figure 73: Powder X-ray diffraction pattern of ZnO nanoparticles prepared by the hydrolysis of $ZnEt_2$ without any added ligand, analysis of the peak width indicates a particle size of ~3.5 nm using the Scherrer equation. The resulting white powder was insoluble.

Supplementary Table 1. Data referring to equilibria between **2X**, **3X** and water **X** = **A** or **B**.

Equilibrium	Ratio (at 0.056 M H_2O) **2X**:**3X** (**X** = **A**, **B**)	^{1}H NMR hydoxide signal for **3X** (**X** = **A**, **B**)	ΔH_r (kJ/mol)	ΔS_r (J/Kmol)
2A⇌3A	1:0.9	δ 3.68	−108±3	−238±9
2B⇌3B	1:0.23	δ 3.81	−97±3	−234±9

Supplementary Table 2. NMR spectroscopy characterisation for **1B** and **5B** presumed to have analogous structures to **1A** and **5A**.

Compound	^{31}P NMR signal (δ)	^{1}H ethyl signals (δ)
**1B**	23.4	0.34, 1.32
**5B**	33.4	−1.39, −0.08, 0.28, 1.25

Supplementary Table 3. Crystallographic data

Compound	**1A**	**2B**	**3A**
CCDC No.	1432882	1432883	1432884
Formula	$C_{56}H_{60}O_8P_4Zn_4$	$C_{98}H_{100}O_{25}P_6Zn_4$	$C_{122}H_{109}O_{21}P_9Zn_6$
M	1246.50	2125.23	2582.24
Crystal System	monoclinic	monoclinic	monoclinic
Space Group	C 2/c	P 2_1/c	P 2_1/c
T [K]	173(2)	173(2)	173(2)
a [Å]	24.3886(5)	26.8366(4)	18.0222(3)
b [Å]	10.1308(2)	19.6190(3)	27.3672(4)
c [Å]	23.8034(5)	18.5191(3)	24.9782(4)
α [deg]	90	90	90
β [deg]	110.684(2)	94.8530(14)	104.1787(18)
γ [deg]	90	90	90
V [Å^3]	5502.16(12)	9715.49(16)	11944.38(19)
Z	4	4	4
Radiation Type	Mo Kα	Mo Kα	Mo Kα
μ (mm^{-1})	1.892	1.149	1.370
θ range [deg]	$2.200 \leq \theta \leq 26.369$	$2.351 \leq \theta \leq 25.027$	$2.182 \leq \theta \leq 26.373$
Reflns collected	15051	32119	42517
R_{int}	0.025	0.022	0.029
No. of data/restr/param	5419 / 0 / 325	16957 / 0 / 1198	22819 / 1002 / 1415
R_1 [I>2σ(I)]	0.0295	0.0386	0.0449
wR_2 [all data]	0.0653	0.0981	0.1015
GoF	0.9733	1.0132	0.9335
Largest diff. pk and hole [e/Å^3]	0.69, -0.47	1.35, -0.74	1.03, -0.75

Compound	**4A**	**5A**
CCDC No.	1432885	1432886
Formula	$C_{124}H_{122}B_3O_{28}P_9Zn_6$	$C_{68}H_{90}O_{12}P_4Zn_{11}$
M	2763.78	1942.53
Crystal System	triclinic	orthorhombic
Space Group	P -1	A b a 2
T [K]	173(2)	173(2)
a [Å]	18.9696(3)	27.6082(4)
b [Å]	19.3442(3)	23.0173(4)
c [Å]	35.8523(7)	24.3319(4)
α [deg]	100.3971(16)	90
β [deg]	98.3638(16)	90
γ [deg]	98.8290(15)	90
V [Å^3]	12582.2(2)	15462.1(3)
Z	4	8
Radiation Type	Cu Kα	Mo Kα
μ (mm^{-1})	2.941	34.84
θ range [deg]	$2.363 \leq \theta \leq 66.601$	$2.231 \leq \theta \leq 25.027$
Reflns collected	69184	40911
R_{int}	0.025	0.040
No. of data/restr/param	43624 / 884 / 3107	12581 / 775 / 857
R_1 [I>2σ(I)]	0.0389	0.0334
wR_2 [all data]	0.1054	0.0767
GoF	0.9889	0.9947
Largest diff. pk and hole [e/Å^3]	1.57, -1.13	0.98, -0.68

Supplementary Note 1

Comparison of 5A and 5C

The species **5C** appears to be analogous to the known structure **5A**. Both form under partial hydrolysis conditions and show two clear ethyl environments in a 4:1 ratio when dissolved in $CDCl_3$. The addition of $ZnEt_2$ to **2C** also establishes an equilibrium with a 5:1 ratio of **1C** to **5C** as is also observed for **2A/1A/5A** and **2B/1B/5B**. Whilst **5A** displays a well-defined diastereotopic relationship between geminal CH_2 protons in one ethyl group in its ^{1}H NMR spectrum this is not observed for **5C**, the addition of bulkier ligands in this case may enforce a more flexible molecular structure.

Supplementary Methods

ZnO@DOPA(5:1) nanoparticles

The synthesis of DOPA capped ZnO nanoparticles by the hydrolysis of $ZnEt_2$ has been previously reported.[5] Briefly, a 5:1 ratio of $ZnEt_2$ and DOPA-H are dissolved in toluene and to this an excess of an acetone solution of water (0.4 M) is added dropwise whilst stirring to induce hydrolysis. The resulting nanoparticles are precipitated with acetone and centrifuged from acetone/toluene to isolate a white powder which is then dried overnight under air. UV, NMR, IR and photoluminescence spectroscopic characterisation along with a representative transmission electron microscopy image are included [Supplementary Figs. 64-72] N.B. The previous study[5] mistakenly identified the sharp ^{31}P NMR signal of by-product **2C** as the ZnO@DOPA nanoparticles themselves, the signal for DOPA coordinated to the nanoparticle surface is in fact very broad and found along the baseline [Supplementary Figure 66:]. **^{31}P{^{1}H} NMR** (162 MHz, C_6D_6): δ ~52 (typical spectrum full width half max. ~ 1800 Hz). Carbon elemental analysis and thermogravimetric analysis were undertaken upon a typical batch of the resulting nanoparticles (estimated size: 2.7 nm by UV spectroscopy, 2-2.5 nm by XRD analysis) to determine the final metal to ligand ratio, both methods generated a consistent result of approximately 6:1. **Elemental Micro-Analysis**: $ZnO(PO_2C_{16}H_{34})x$, found C, 25.27. Corresponding to 38% weight of ligand in the sample and a metal to ligand ratio of 5.8:1. **Thermogravimetric Analysis**: 29.1% mass loss (120-550° C) expected from the loss of octyl chains of the ligand. This corresponds to 37% weight of ligand in the sample and a metal to ligand ratio of 6:1.

Van't Hoff analysis

To test the equilibrium between **2A/B** and **3A/B** variable temperature NMR spectra were recorded between 288 and 328 K. After reaching the required temperature ^{1}H and ^{31}P{^{1}H} spectra were recorded, these were repeated after a waiting period of at least 15 minutes. All repeat spectra show the same ratio of species as the initial spectrum indicating that equilibrium is established quickly at each temperature. The ratio of **2A/B** to **3A/B** was calculated from integral analysis of the ^{31}P{^{1}H} spectra and from this the concentrations of both species were calculated considering the known amount of ligand. The ratio of water to **3A** (for **2A/3A**) and to **2B** (for **2B/3B**) was calculated from integral analysis of the ^{1}H NMR spectrum comparing the dissolved water signal (δ ~1.62, slight temperature dependence) to a clear aromatic resonance of known relative integral (for **3A** δ 7.82, for **2B** δ 6.58).

Plotting a graph of $\ln(K_{(eq)})$ vs 1/T allows estimation of the enthalpy and entropy of reaction

Equilibrium between **2A**, H_2O and **3A**

$$3\ \mathbf{2A} + 3\ H_2O \rightleftharpoons 2\ \mathbf{3A}$$

$$K_{(eq)} = \frac{[\mathbf{3A}]^2}{[\mathbf{2A}]^3 \times [H_2O]^3} \quad \text{(eq. 1)}$$

2A (19.4 mg, 0.0123 mmol) was dissolved in $CDCl_3$ (0.47 mL 0.701 g) in a Young's tap NMR tube and, to this, water (0.5 µL, 0.028 mmol) was added, by Eppendorf syringe, after which the tube was sealed to avoid any solvent evaporation.

Equilibrium between **2B**, H_2O and **3B**

$$3\ \mathbf{2B} + 3\ H_2O \rightleftharpoons 2\ \mathbf{3B}$$

$$K_{(eq)} = \frac{[\mathbf{3B}]^2}{[\mathbf{2B}]^3 \times [H_2O]^3} \quad \text{(eq. 1)}$$

An identical series of VT-NMR experiments as per the experiments using **2A/3A**, were conducted to study the equilibrium.

2B (23.6 mg, 0.0122 mmol) was dissolved in $CDCl_3$ (0.47 Ml, 0.70 g) in a Young's tap NMR tube and, to this, water (0.5 µL, 0.028 mmol) was added, by Eppendorf syringe, after which the tube was sealed to avoid any solvent evaporation.

NMR Study of the Equilibrium between 1A/B, 2A/B, 5A/B and $ZnEt_2$

2A (10(±0.5) mg, 6.3 µmol) [or **2B** (12.3(±0.5) mg, 6.3 µmol)] were measured into a NMR tube and dissolved in $CDCl_3$ (~0.4 mL) at room temperature. To this, a solution of $ZnEt_2$ (100 µL from a 0.237 M stock solution in $CDCl_3$) was added. This created an approximately 4:15 ratio of **2A/B** to $ZnEt_2$. The resulting solution was analysed by $^{31}P\{^1H\}$ and ^1H NMR spectroscopy. This revealed that in both cases a 5:1 ratio of new products appeared (e.g. 5 **1A/B** : 1 **5A/B**), however, rather than a complete reaction some of both **2A/B** and $ZnEt_2$ are retained indicative of an equilibrium between the four species.

Starting with **2A**, The final ratios determined by integral analysis were: 4 **2A** : 7 $ZnEt_2$: 5 **1A**: 1 **5A**. This shows that the equilibrium position is at around 50% conversion from **2A** to **1A** and **5A**.

Starting with **2B**, The final ratios determined by integral analysis were: 3 **2B** : 4 $ZnEt_2$: 5 **1B**: 1 **5B**. This shows that the equilibrium position is at around 57% conversion to **1B** and **5B**. N.B. in this case an 'unknown' species is also observed with an integral similar in magnitude to **5B**.

This experiment shows the generality of structures **1A** and **5A** in the alternative phosphinate (D^{Meo}PPA) system. Whilst **1B** and **5B** have not been isolated we can be confident from the similar reactivity and chemical shifts to **1A** and **5A** that they form in this case.

A variable temperature study was conducted on the $ZnEt_2$/**2A** ⇄ **1A**/**5A** system, the sample was allowed to equilibrate for 25 minutes at each temperature. The same preparation was used as described above, in this case a ratio of 0.4 **2A** : 7 $ZnEt_2$: 5 **1A**: 1 **5A** was initially obtained at room temperature, suggesting slightly less than 4 equivalents of **2A** were measured (relative to 15 $ZnEt_2$). The NMR tube was heated to 298, 308 and 318 K to determine the response to increasing temperature. The ratio adjusts to approximately 1.1 **2A** : 9 $ZnEt_2$: 5 **1A**: 1 **5A** at 308 K and to 1 **2A** : 13 $ZnEt_2$: 3 **1A**: ~0 **5A** at 318 K, although it must be stressed that impurities grow in upon heating which hinder accurate analysis. On cooling the sample back to room temperature, the **2A** signal

reduces and a trace of **5A** grows back in, however, decomposition products now appear as a significant fraction.

***In situ* NMR spectroscopy study of the synthesis of ZnO nanoparticles by hydrolysis route**

A Young's NMR tube was loaded with 14 mg (0.048 mmol) of di-octylphosphinic acid and a capillary containing a CD_2Cl_2 solution of PPh_3 was added as an internal standard. 0.5 ml of d_8-toluene was then added followed by 30 mg (0.243 mmol) of $ZnEt_2$. The resulting mixture was analysed by NMR spectroscopy. Under a flow of N_2 0.5 µL (0.028 mmol) aliquots of water were added sequentially (with NMR spectra recorded between samples, each approximately 30 minutes after the addition of water). Upon addition of 2 µL of water (50% hydrolysis) the mixture was left to stand overnight and NMR spectra recorded again after a 15 hour pause. Further additions of water were then conducted as before until 4 µL of water had been added (a further 0.5 µL was also added to ensure full hydrolysis had occurred). The total reaction can be described as:

$$5\ ZnEt_2 + HOOP(C_8H_{17})_2 + 4.5\ H_2O \longrightarrow Zn_5O_{4.5}(DOPA) + 10\ EtH$$

***In situ* UV spectroscopy study of the synthesis of ZnO nanoparticles by hydrolysis route**

Di-octylphosphinic acid (51.7 mg, 0.18 mmol)) was dissolved in 12 ml of toluene and to this $ZnEt_2$ (110 mg, 0.89 mmol) was also added. The solution was split into four 3 mL fractions and 1, 2, 3 and 4 µL of water were added to each fraction respectively giving 25%, 50%, 75% and complete hydrolysis (of which those at 50% and 75% displayed a yellow colour). The solutions were stirred for one hour and then diluted and sealed into UV cuvettes (using a glovebox for the partially hydrolysed samples) at [Zn] = $4x10^{-3}$ M. UV spectra were recorded over the 400-290 nm region. A control reaction revealed that a solution of **1C** (e.g. 0% hydrolysis) did not absorb in this UV region, nor did the 25% hydrolysed solution.

Synthesis and characterisation of 'yellow ZnO'

To 25 mL of toluene, $ZnEt_2$ (500 mg, 4.05 mmol) was added and then under a flow of N_2 0.75 equivalents of H_2O (54.6 µL, 3.03 mmol) was added quickly. Since water is not very miscible with toluene the reaction proceeds over several minutes (ethane gas is evolved and the flask was left open to a bubbler). The mixture was stirred for 30 minutes with periodic sonication to make sure all moisture is evenly incorporated. A yellow solution forms. The solvent was removed by vacuum to leave a glassy yellow powder. This powder does not immediately change colour under air, however over prolonged periods (>1 week) it becomes white. Once isolated as a solid it is insoluble.

Elemental analysis: 5.9% Carbon, 1.7% Hydrogen

X-ray Crystallography Details

X-ray crystallography data for **1A**, **2B**, **3A** and **5A** were collected on an Oxford Diffraction diffractometer using graphite monochromated Mo Kα radiation (λ = 0.71073 Å) and a low-temperature device [173(2) K];[6] X-ray crystallography data for **4A** was collected on an Agilent SuperNova diffractometer using graphite monochromated Cu Kα radiation (λ = 1.54180 Å) and a low-temperature device [173(2) K].[6] Data were collected using SuperNova, reduction and cell refinement was performed using CrysAlis.[7] The structure was solved by direct methods using Superflip [8] and refined full-matrix least squares on F^2 using CRYSTALS.[9] All non-hydrogen atoms were refined with anisotropic displacement parameters. All hydrogen atoms were placed in calculated positions and refined before applying the riding model. Crystallographic data have been deposited with the Cambridge Crystallographic Data Centre under CCDC 1432882-1432886. These data can be obtained free of charge from The Cambridge Crystallographic Data Centre via

www.ccdc.cam.ac.uk/data_request/cif. Full bond length and bond angle data may be found in the CIFs.

Special refinement details:

3A: Two solvent molecules of toluene were located in the Fourier map, one could be sensibly refined with the use of symmetry restraints, however, the other could not be adequately modelled due to extensive disorder and so was treated using the SQUEEZE algorithm.[10] Two of the phenyl groups showed minor disorder in the form of enlarged displacement ellipsoids, one was modelled over two positions and restrained to maintain sensible geometries, the other was best modelled by adding symmetry restraints to the original position. Hydrogen atoms upon the OH groups (H192, H203 & H212) could not be located in the Fourier map and were placed in calculated positions before allowing free refinement and then applying the riding model. We anticipate these hydroxide protons could be disordered over different sites but this model does not include this.

4A: Eight solvent molecules of THF were located in the structure. Inspection of the Fourier map allowed identification of the oxygen atom (with greater electron density) in each case. Symmetry restraints were applied to each THF molecule and all eight molecules were restrained to each other to exhibit similar connectivity. One molecule of THF was split into two components with the occupancy of each part refined.

5A: Shift limiting restraints were added to aid initial refinement but were removed once the structure was correctly located, further refinement was conducted. Symmetry restraints were added to two phenyl groups which showed mild disorder. A racemic mixture is apparently present in the structure (Flack parameter = 0.397(10)), the approximately spherical shape may allow both enantiomers to co-crystallise.

Supplementary References

1. Kisi, E.H. & Elcombe, M.M. u parameters for the wurtzite structure of ZnS and ZnO using powder neutron diffraction. *Acta Crystallogr. Sect. C* **45**, 1867-1870 (1989).
2. Meulenkamp, E.A. Synthesis and Growth of ZnO Nanoparticles. *J. Phys. Chem. B* **102**, 5566-5572 (1998).
3. Coppel, Y. et al. Full Characterization of Colloidal Solutions of Long-Alkyl-Chain-Amine-Stabilized ZnO Nanoparticles by NMR Spectroscopy: Surface State, Equilibria, and Affinity. *Chem.-Eur. J.* **18**, 5384-5393 (2012).
4. Ali, M. & Winterer, M. ZnO Nanocrystals: Surprisingly 'Alive'. *Chem. Mater.* **22**, 85-91 (2010).
5. Brown, N.J., Weiner, J., Hellgardt, K., Shaffer, M.S.P. & Williams, C.K. Phosphinate stabilised ZnO and Cu colloidal nanocatalysts for CO2 hydrogenation to methanol. *Chem. Commun.* **49**, 11074-11076 (2013).
6. Cosier, J. & Glazer, A.M. A Nitrogen-Gas-Stream Cryostat for general X-Ray-Diffraction Studies. *J. App. Cryst.* **19**, 105-107 (1986).
7. Oxford Diffraction Ltd, Abingdon, England, 2011
8. Palatinus, L. & Chapuis, G. SUPERFLIP - a computer program for the solution of crystal structures by charge flipping in arbitrary dimensions. *J. Appl. Crystallogr.* **40**, 786-790 (2007).
9. Betteridge, P.W., Carruthers, J.R., Cooper, R.I., Prout, K. & Watkin, D.J. CRYSTALS version 12: software for guided crystal structure analysis. *J. Appl. Crystallogr.* **36**, 1487 (2003).
10. Spek, A.L. Single-crystal structure validation with the program PLATON. *J. Appl. Crystallogr.* **36**, 7-13 (2003).
